# Dihydroxyquingdainone Induces Apoptosis in Leukaemia and Lymphoma Cells via the Mitochondrial Pathway in a Bcl-2- and Caspase-3-Dependent Manner and Overcomes Resistance to Cytostatic Drugs In Vitro

**DOI:** 10.3390/molecules27155038

**Published:** 2022-08-08

**Authors:** Jennifer Baas, Sebastian Bieringer, Corazon Frias, Jerico Frias, Carolina Soehnchen, Corinna Urmann, Steffi Ritter, Herbert Riepl, Aram Prokop

**Affiliations:** 1 Department of Pediatric Hematology/Oncology, Helios Clinic Schwerin, Wismarsche Straße 393-397, 19055 Schwerin, Germany; 2 Department of Pediatric Hematology/Oncology, Municipal Clinics of Cologne, Children’s Hospital of the City Cologne, Amsterdamer Straße 59, 50735 Cologne, Germany; 3 Organic-Analytical Chemistry, Weihenstephan-Triesdorf University of Applied Sciences, 94315 Straubing, Germany; 4 TUM Campus Straubing for Biotechnology and Sustainability, Technical University of Munich, 94315 Straubing, Germany; 5 Medical School Hamburg (MSH), University of Applied Sciences and Medical University, Am Kaiserkai 1, 20457 Hamburg, Germany

**Keywords:** *Isatis tinctoria*, quingdainone, tryptanthrin, Nalm-6, BJAB, intrinsic apoptosis pathway

## Abstract

*Isatis tinctoria* and its indigo dyes have already provided highly active anti-leukaemic lead compounds, with the focus mainly being on indirubin, whereas indigo itself is inactive. There are many more indigoids to find in this plant extract, for example, quingdainone, an indigoid derived from tryptanthrin. We present here a new synthesis of hitherto neglected substituted quingdainones, which is very necessary due to their poor solubility behaviour, and a structure-dependent anti-leukaemic activity study of a number of compounds. Substituted α-phenylaminoacrylic acid was synthesised by hydrogen sulfide extrusion from an analogue mercaptoacetic acid, available from the condensation of rhodanin and a substituted tryptanthrin. It is shown that just improving water solubility does not increase anti-leukaemic activity, since a quingdainone carboxylic acid is inactive compared to dihydroxyquingdainone. The most effective compound, dihydroxyquingdainone with an AC_50_ of 7.5 µmole, is further characterised, revealing its ability to overcome multidrug resistance in leukaemia cells (Nalm-6/BeKa) with p-glycoprotein expression.

## 1. Introduction

**Figure 1:**
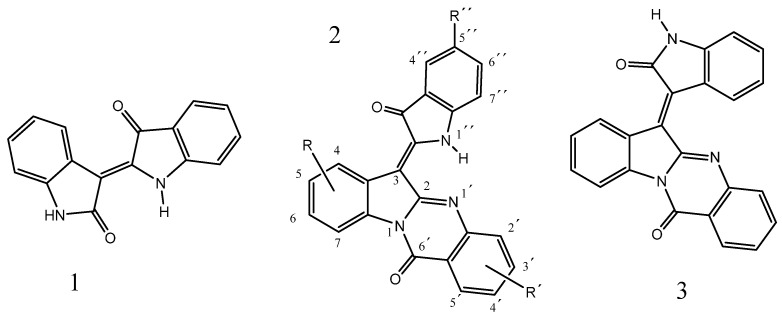
Structures of indirubin **1**, candidine/quingdainone **2,** i.e., (Z)-6-(3-oxoindolin-2-ylidene)indolo[2,1-b]quinazolin-12(6H)-one, and its isomer (E)-6-(2-oxoindolin-3-ylidene)indolo[2,1-b] quinazolin-12(6H)-one **3,** originating from isoindigo.

Extracts from the natural dye and medicinal plants *Isatis tinctoria*, *Strobilanthes cusia* and other plants yielding the indigo dye have long been known as anti-inflammatory agents in traditional Chinese medicine (TCM). They can be applied as anti-leukaemic agents, as proven by Chinese small-scale clinical trials in the nineteen-eighties, whereas in Europe, any phytopharmaceutical use of woad has been forgotten, despite its centuries-long use here. The anti-leukaemic activity was traced to indirubin **1**, an isomer of bisindole-heterocyclic structure. Some of its synthetic derivatives were recognised as potent cell cycle inhibitors [1] with efficacy far beyond that of indirubin itself. It has been found that the indirubin-5-sulfonate **1a** fits perfectly in the ATP niche of CDK-1 and thereby blocks its activity [2]. All these compounds are formed from “indoxyl” precursors such as isatane and various ketones present in the plants during post-harvest treatment in the moist state or by extraction with aqueous fluids. In this manner, the presence of indigo brown [3] (Figure 1) can be explained, also known as candidine or quingdainone **2**, another dark dye in fermentation solutions from *Candida lipolytica* [4,5], and also from an orchid, *Phaius mishmensis* [6]. It is formed from an indoxyl precursor with indoloquinazolinone (tryptanthrin). It can be distinguished by its UV/VIS-spectrum (CHCl_3_, (log ε): 244 (4.34), 250 (4.36), 282 (4.21), 538 (3.95), 573 (4.11)) from other purple/red indigoid heterocycles, for example, from **3**, (E)-6-(2-oxoindolin-3-ylidene)indolo[2,1-b]quinazolin-12(6H)-one, a crossbreed of 2-oxindole and tryptanthrin (which can also be seen as a product of isoindigo).

The bioactivity of **2** has been explored insufficiently and has its origin in the very poor solubility of this compound. Quingdainone has been found to possess anti-cancer activity [7]. On the other hand, one wonders whether the tumor-inhibitory activity with unsubstituted indigoids in general could be observed at all, since the solubility of this compound is nearly zero in water. We therefore synthesised quingdainones with improved solubilities and screened these compounds for anti-leukaemic properties.

## 2. Results

### 2.1. Chemistry

**Scheme 1:**
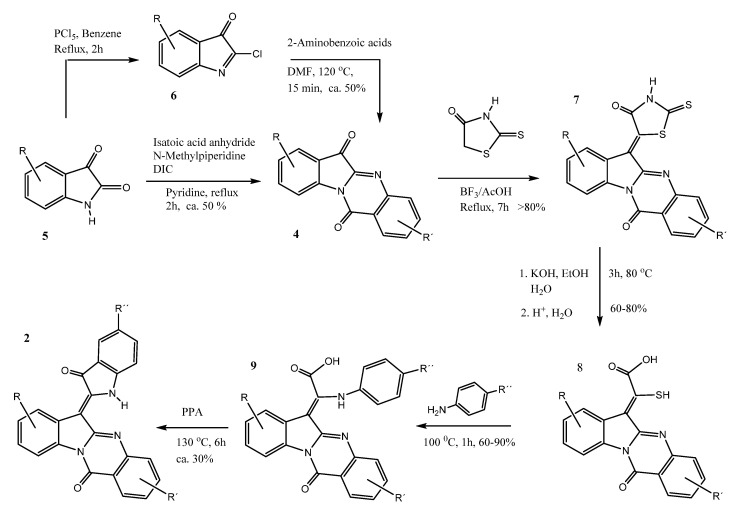
Overview of all reactions of the quingdainone synthesis. The double bond (3-2’’) is portrayed with the correct Z-configuration. Precursors are also depicted with Z-configuration; however, E-configuration can be present as well.

The accessibility of indoxyl derivates has always been a problem in the preparation of indigoids. The method reported within this paper provides a way to introduce different substituents based on an α-phenylaminoacrylic acid methodology. The indoxyl part is built up by using ylidenoxindoles in our case, from tryptanthrins **4**, by means of the sequential introduction of anilines followed by ring-closing. This enables one to have a wide variety of different substituents. The presented syntheses have moderate to good yields and need no or only one column purification at the end. It was planned to convert obtained tryptanthrins **4** in a first step to a condensation product **7** with 2-thioxo-4-thiazolidinone (rhodanin) (see Scheme 1), followed by hydrolytic ring opening to a mercaptoacetic acid **8**. The next sequence was the substitution of the mercapto group with various anilines to yield cyclisable molecules **9** to the final quingdainones **2a–f**. The first step already proved to be comparably difficult. This condensation could possibly be completed in a few minutes with isatins **5**, as even boiling for a long time is not sufficient for a reaction with the 6-keto group in tryptanthrins. Only a solvent with a large amount of Lewis acid (BF_3_) in acetic acid promoted the condensation sufficiently to yield **7**. Substituents at position 4 of isatins prolong the condensation, and a substituent at position 7 in our case of tryptanthrins renders the reaction impossible.

**Figure 2:**
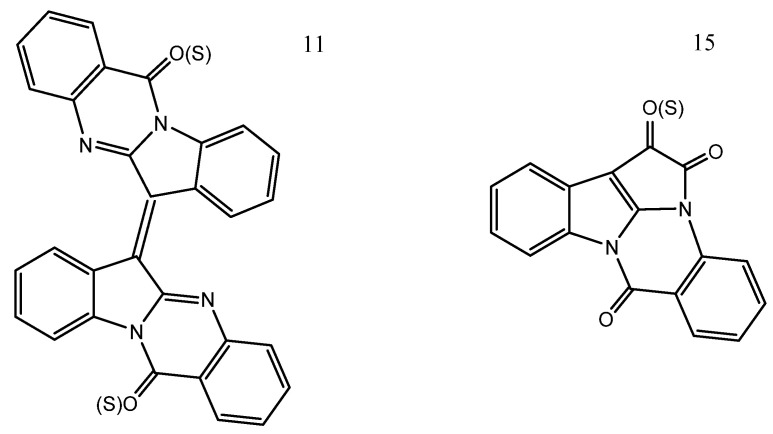
Proposed structures of side products from the isatin/indirubins synthetic procedure applied to tryptanthrin.

Furthermore, the cleavage of the rhodanin-adduct **7** with KOH/H_2_O took a different route, since it yielded more products. Intense violet flakes could be obtained from the hydrolysis solution in amounts not to be neglected. This material can be shown by DC to consist of two compounds. Tryptanthrin **4** was reported to be reduced by agents such as hypophosphorous acid to yield dihydroditryptanthrin **10** [8], which is oxidised to “ditryptanthrin” (**11**, (6E)-6-(12-oxoindolo[2,1-b]quinazolin-6-ylidene)indolo[2,1-b] quinazolin-12-one) (Figure 2). Since there are enough reactive reducing SH molecules present during the hydrolysis reaction of the thioxothiazolidinones, we speculated on what the side products might be. DC supports the identity of the minor compound in the “violet flakes” being **11**, while it has very low solubility like **11**. Of interest is the nature of the second intense violet product obtained from the “flakes”. Since it was insoluble in basic aqueous media, it is suspected not to be an ionic molecule, while there is a difference in its solubility in methanol or acetone. Bergman also reports some stable thioketones of tryptanthrin and ditryptanthrin, prepared by oxygen–sulphur exchange [9]. Regarding the idea that such an exchange might have been feasible with thioxothiazolidinone on tryptanthrin, its absolute insolubility in everything disproves such an idea. On the other hand, a simple ring-closing reaction of the thioacetic acids **8** could possibly yield a thioketone **15** of a dark colour.

Despite these difficulties, substantial amounts of mercaptoacetic acids **8** after acidic work-up were obtained. Some of them could better be characterised as allylthio derivatives, which crystallise far better. We projected our tryptanthrin-derived mercaptoacetic acids to react analogously with anilines in a DME solution catalysed with trifluoroacetic acid, as reported earlier [10], but this method did not work here. Gränacher et al. already described that mercaptoacetic acids from isatins react with liquid anilines under the evolution of H_2_S [11]. Accordingly, melting **8** at 100 °C for 1 h with different para-substituted anilines for the sake of the simplification of the NMR spectra was attempted to yield **9**. The evolution of H_2_S and formation of a yellow colour indicated a successful reaction. Subsequently, excess aniline was removed by sublimation. After an acid/base aqueous work-up procedure, the remaining contamination was a small amount of the para substituted aniline, which was finally removed under high vacuum (10^−3^ mbar) at 130 °C. When a higher temperature than this was used, a second set of small signals next to the ones from the original compound were observed in ^1^H-NMR. This can most likely be interpreted as isomerisation of the double bond of **9**. The last step, a cyclisation under *Nazarov* conditions in polyphosphoric acid (PPA), yielded quingdainones **2a–g** without further complications (see Table 1).

The question as to whether the quingdainone is an ***E*** or ***Z*** isomer has been addressed [12]. Unambiguously, it is of ***Z***-geometry, as shown by ^1^H-NMR through space coupling of the 1’’ NH proton to aromatic proton 2’ and not to 4. No other isomer has been found. It has the same configuration lock that occurs in indigo itself by the hydrogen bridge of the indoxyl NH to O of the oxindole moiety. Since substitution sites of the synthesised compounds **2a–g** are far from this hydrogen bridge area, it is assumed that all the compounds are Z isomers.

Water solubility was installed with **2f,g**. As acidic molecules, **2f,g** are soluble in a dilute base from pH > 7. When precursor **9g** (R,R’’ = OMe,) was cyclised in PPA, a product **2g** with a surprisingly good water solubility was obtained. Usually, quingdainones separate out of the water solution of PPA during work-up. In this case, an adsorbent had to be used to extract **2g**. ^1^H-NMR of this compound showed none of the expected methoxy signals, and a mass from HRMS interpreted as R,R’’ = OH proved the notion that the methoxy groups have been demethylated. Different examples from the literature also report cleavage of OMe groups and esters in PPA at higher temperatures [13,14].

### 2.2. Quingdainones Cause Apoptosis in Nalm-6 Cells

In order to examine the biological activity of new quingdainone derivates, we first measured DNA fragmentation as evidence for the late apoptotic phase [15] in leukaemia cells (Nalm-6) after 72 h of treatment. Only a few compounds were able to induce apoptosis in the measured concentrations up to 50 µM (see Table 2). Dihydroxyquingdainone (**2g**) turned out to be the most promising compound, so we further characterised its effects in malignant cells; it has an AC_50_ of 7.5 μM, which is the concentration of compounds able to induce apoptosis in 50 percent of the Nalm-6 cells after 72 h of incubation [16,17,18,19,20]. The non-substituted original quingdainone does not induce apoptosis in concentrations up to 100 µM.

**Figure 3:**
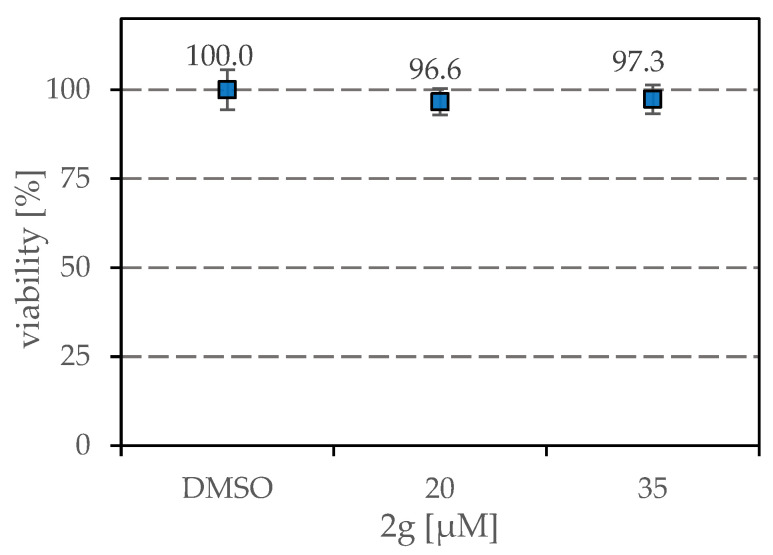
Exclusion of **2g**-induced necrosis in Nalm-6 cells. Nalm-6 cells were treated with 20 µM and 35 µM **2g** for 1 h. The determination of viability was performed via the LDH release assay. Three replicates were carried out per concentration. The mean values ± SD are shown; no significance was found (*p* < 0.05 vs. DMSO, *t*-test).

With a view to clinical application, necrosis could be excluded as a non-specific form of cell death. For this purpose, the enzyme lactate dehydrogenase released by cell lysis was quantified colorimetrically after treating the cells with **2g** for 1 h. It could be shown that the viability of the cells was not significantly reduced by relevant concentrations of **2g** (see Figure 3).

### 2.3. Dihydroxyquingdainone ***2g*** Shows Anti-Proliferative Effects in Nalm-6 Cells

**Figure 4:**
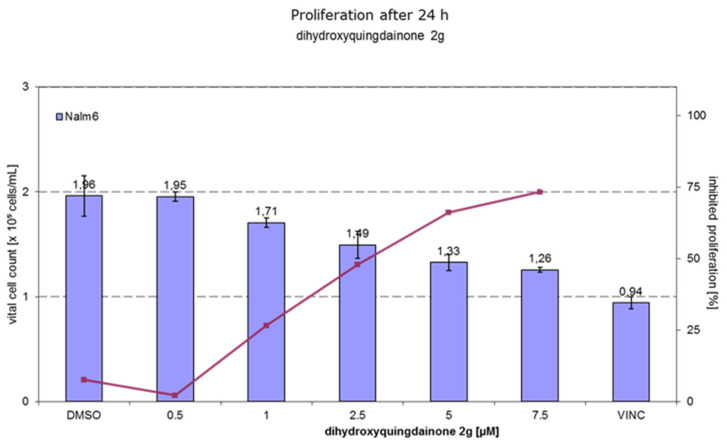
Anti-proliferative effects of **2g** in 1 × 10^5^ Nalm-6 cells/mL after treatment for 24 h. The number of Nalm-6 cells was determined by CASY^®^ Cell Counter after 24 h of incubation with different concentrations of **2g**. Three replicates were carried out per concentration. The mean values ± SD are shown (*p* < 0.05 vs. DMSO, *t*-test). The DMSO control without **2g** shows a cell proliferation from 1 × 10^5^ Nalm-6 cells/mL up to 1.96 × 10^5^ Nalm-6 cells/mL. Dihydroxyquingdainone **2g** inhibits the proliferation of leukaemia cells in a dose-dependent manner. Vincristine (VINC) is the positive control (no cell proliferation after 24 h—100% inhibition of proliferation after treatment with 22.7 nM vincristine for 24 h).

For the tumour therapy, the inhibition of proliferation of cells is important to stop the spread of malignant cells. To prove this, Nalm-6 cells were treated with different concentrations of **2g** for 24 h before the cell count was determined via the CASY Cell Counter and Analyzer System. Figure 4 shows the reduction in cell count and thus an inhibition of proliferation in a dose-dependent manner.

### 2.4. Dihydroxyquingdainone ***2g*** Involves the Intrinsic Apoptosis Pathway

Two major pathways have been found to induce apoptosis: the extrinsic and intrinsic pathway [21,22]. The latter is mitochondrially mediated and can be triggered via, i.a., ROS or DNA damage. The permeabilization of the mitochondrial outer membrane (MOMP) results in the release of pro-apoptotic proteins, which in further steps activate procaspase 9. As a result, effector caspase-3 is processed, which then initiates cellular changes of apoptosis [23,24].

**Figure 5:**
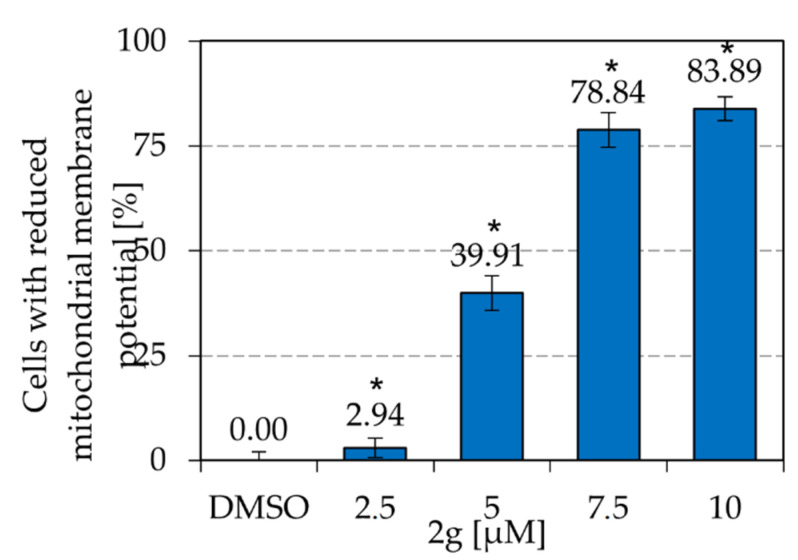
Reduction in mitochondrial membrane potential induced by **2g**. Nalm-6 cells were incubated with different concentrations of **2g** for 48 h. The percentage of cells with reduced mitochondrial membrane potential was determined using JC-1 staining and subsequent flow cytometric measurement. Three replicates were carried out per concentration. The mean values ± SD are shown (* *p* < 0.05 vs. DMSO, *t*-test).

To investigate the apoptosis induction via the intrinsic pathway, mitochondrial membrane potential, which is changed as a result of MOMP, could be examined. Therefore, Nalm-6 cells were incubated with **2g** for 48 h and subsequently stained with JC-1. The results show a significant increase in the proportion of cells with reduced mitochondrial membrane potential (see Figure 5), which is a direct indication of the involvement of the intrinsic pathway.

**Figure 6:**
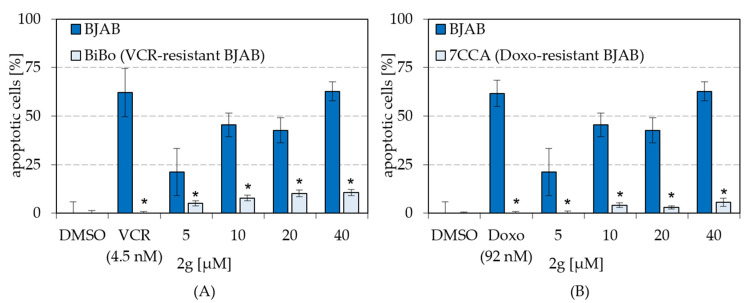
The **2g**-induced apoptosis depends on Bcl-2 and caspase-3. BJAB cells as well as their corresponding resistant cell lines were treated with **2g** for 72 h. As the solvent, control cells were treated with the same amount of DMSO as used for **2g**. The cytostatic drugs were used to prove the resistance of the cells ((**A**): VCR—vincristine, (**B**): Doxo—doxorubicin). After treatment, the induction of apoptosis was measured via flow cytometric analysis of DNA fragmentation. Three replicates were carried out per concentration. The mean values ± SD are shown (* *p* < 0.05 vs. corresponding concentration in BJAB, *t* test).

**Figure 7:**
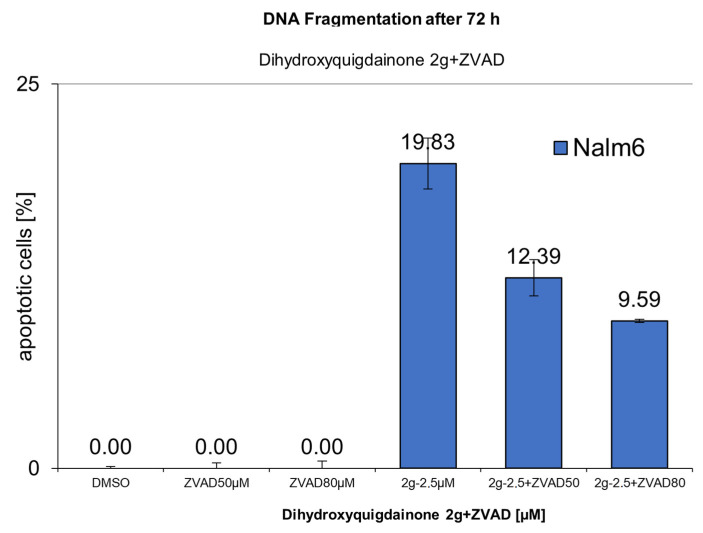
The apoptosis induction of **2g** is caspase-dependent. Nalm-6 cells were treated with two concentrations of the pan-caspase inhibitor Z-VAD, with **2g** and with **2g** together with two concentrations of the pan-caspase inhibitor Z-VAD for 72 h. As the solvent, control cells were treated with the same amount of DMSO as used for **2g**. After treatment, the induction of apoptosis was measured via flow cytometric analysis of DNA fragmentation. Three replicates were carried out per concentration. The mean values ± SD are shown (*p* < 0.05 vs. corresponding concentration in Nalm-6, *t*-test). The pan-caspase inhibitor Z-VAD is able to inhibit the **2g**-induced apoptosis significantly. Z-VAD alone do not induce apoptosis. These data demonstrate that dihydroxyquingdainone **2g**-induced apoptosis is dependent on the activity of caspases in tumour cells.

Furthermore, the induction of apoptosis by **2g** depends on the level of expression of Bcl-2. BiBo cells (vincristine-resistant BJAB cells) show an overexpression of this protein. We compared the apoptosis induction via **2g** in primary BJAB and BiBo cells. It was shown that **2g** induces significantly less apoptosis in BiBo cells than in BJAB cells, which is why a dependence on Bcl-2 can be assumed (Figure 6A). This is another hint for the involvement of the intrinsic pathway. In the same way, a dependence on capase-3 was proven. The pan-caspase inhibitor Z-VAD is able to inhibit the **2g**-induced apoptosis significantly. Z-VAD alone do not induce apoptosis (see Figure 7). These data demonstrate that dihydroxyquingdainone **2g**-induced apoptosis is dependent on the activity of caspases in tumour cells. The number of induced apoptotic cells by 2g in BJAB cells was significantly higher than in 7CCA cells (doxorubicin-resistant BJAB cells). Moreover, 7CCA cells show an underexpression of caspase-3, so **2g** induces apoptosis depending on the level of caspase-3 (Figure 6B).

### 2.5. Dihydroxyquingdainone ***2g*** Overcomes Resistances to Cytostatic Drugs

The resistance of malignant cells to conventional cytostatic drugs is a major problem in the therapy of tumours and lowers the chances of achieving a cure [25,26]. To illustrate this, Nalm-6 cells were made resistant to various cytostatic drugs. To test whether **2g** is able to overcome resistance, these different cell lines were treated with the quingdainone derivate **2g**.

BeKa cells, which are vincristine (VCR)-resistant Nalm-6 cells, show an expression of the p glycoprotein compared to the initial cells. Substances can be actively secreted from the cell via this membrane protein, whereby they lose their effect [27,28]. This mechanism often results in multidrug resistance (MDR) [29], which has also been demonstrated for BeKa cells. BeKa cells show a co-resistance to anthracyclines (idarubicin, daunorubicin, doxorubicin, epirubicin), mitoxantrone, fludarabine, vinca alkaloids (vincristine, vindesine, vinorelbine, vinblastine) and etoposide in vitro [30].

**Figure 8:**
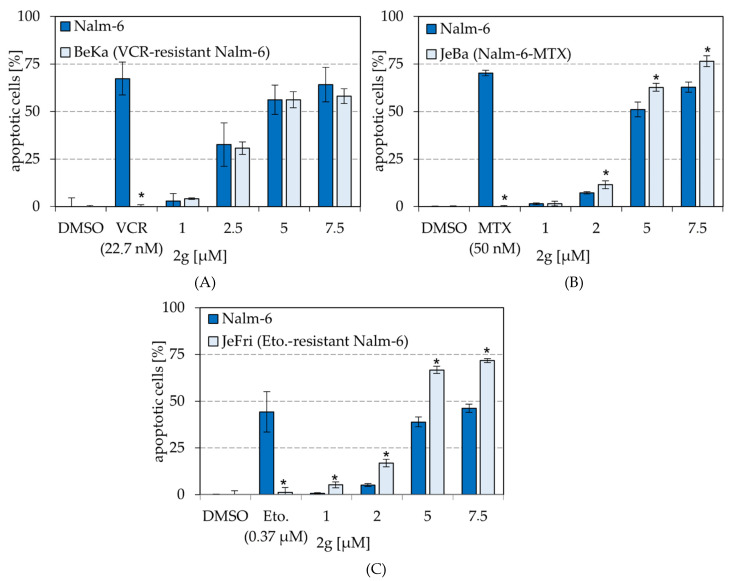
Resistance overcoming by **2g**. Nalm-6 cells as well as their corresponding resistant cell lines were treated with **2g** for 72 h. DMSO was used as the solvent. The cytostatic drugs were used to prove the resistance of the cells ((**A**): VCR—vincristine, (**B**): MTX—methotrexate, (**C**): Eto.—etoposide). After treatment, the induction of apoptosis was measured via flow cytometric analysis of DNA fragmentation. Three replicates were carried out per concentration. The mean values ± SD are shown (* *p* < 0.05 vs. corresponding concentration in Nalm-6, *t*-test).

Nalm-6 and BeKa cells were treated with **2g** for 72 h before DNA fragmentation was performed. The comparison of the percentage of apoptotic cells in these **2g**-treated cell lines showed no significant differences (see Figure 8A). Thus, **2g** is not a substrate of the p-glycoprotein and overcomes multidrug resistance in leukaemia cells. Similar results have been found for JeBa and JeFri cells, which are Nalm-6 cells resistant to methotrexate (MTX) and etoposide (Eto.), respectively. The mechanisms of resistance have not been elucidated yet. Dihydroxyquingdainone **2g** overcomes both resistances and shows even higher effects in the resistant cells compared to Nalm-6 cells (see Figure 8B,C).

### 2.6. Dihydroxyquingdainone ***2g*** Causes Significantly Less Apoptosis in Healthy Human Leukocytes Than in Nalm-6

**Figure 9:**
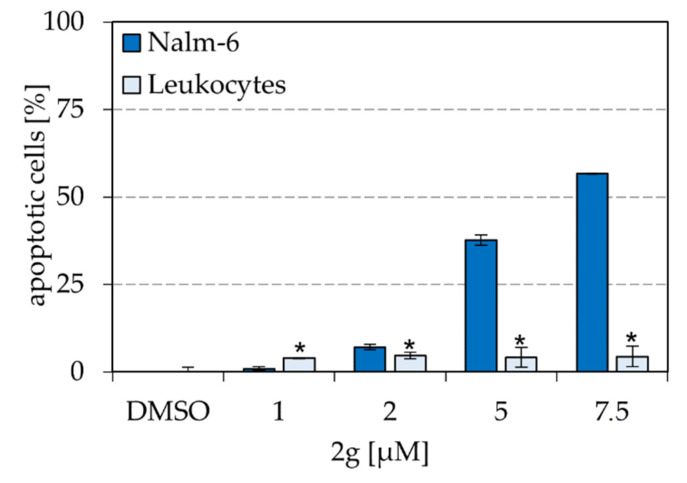
Selectivity of **2g**. Nalm-6 cells and healthy human leucocytes (ex vivo) were treated with different concentrations of **2g** for 72 h. As the solvent, control cells were treated with the same amount of DMSO as used for **2g**. After treatment, the induction of apoptosis was measured via flow cytometric analysis of DNA fragmentation. Three replicates were carried out per concentration. The mean values ± SD are shown (* *p* < 0.05 vs. corresponding concentration in Nalm-6, *t*-test).

Selectivity of cytostatic drugs is very important to ensure that only malignant cells and no healthy cells are affected. Most of the conventional agents cause side effects because of their limited selectivity against cancer cells [31]. A first test to determine the selectivity of a new potential agent is to test the effect on healthy leukocytes (ex vivo). For **2g**, a significantly reduced apoptosis in leukocytes compared to malignant Nalm-6 cells was shown (Figure 9).

## 3. Discussion

The interest in natural compounds for use as anti-cancer therapeutic agents is very high due to the wide range of biological activities found in different kingdoms of life [32]. Several of today’s important cytostatic drugs are based on natural substances. Vinca alkaloids such as vincristine or vinblastine as well as taxanes (paclitaxel, docetaxel) were initially isolated from plants [33,34]; nevertheless, the occurrence of resistance demands ongoing research. For centuries, indigo plants have been used in traditional medicine. Some ingredients of Qing Dai, an indigo-containing preparation still used today which consists of extracts from different herbs in traditional Chinese medicine, show anti-tumour effects [35]. The responsible molecules also include quingdainone **2** (R=R’=R’’= H) [7]. Derivatives were investigated here in comparison with the original, since it was speculated that a more water-soluble derivative might be more suitable for the elucidation of its activity. Adding neutral substituents (**2a**–**e**) to the aromatic cores does not improve solubility in aqueous media and does not improve it so much in organic solvents, so the lack of cytotoxicity seems to reflect this. However, just improving water solubility does not improve cytotoxicity. Rather, it was abolished, since in the carboxylic acid **2f**, the respective AC_50_ is above 50 mmol or more like in **2a**–**e**. The most effective compound dihydroxyquingdainone **2g** showed in vitro cytotoxic effects in leukaemia cells and was able to overcome various cytostatic resistances, making it a potential new agent for the therapy of malignant diseases. Apoptosis was induced with a half maximal concentration (AC_50_) of <7.5 µM (Table 2) after 72 h of incubation.

Another important property for treating cancer is to inhibit the proliferation of malignant cells. This could be proven for **2g** in Nalm-6 cells (Figure 4). Fortunately, necrosis was excluded as a non-specific form of cell death for **2g**, which would otherwise have been an exclusion criterion for further investigation (Figure 3).

Another highly important property of **2g** is the selective induction of apoptosis, which means that non-proliferating healthy human cells such as leukocytes are hardly affected (Figure 9). It would also be interesting to find out how healthy human proliferating cells react, which could be achieved in further experiments. In order to assess the efficacy of **2g**, it is also very important to investigate the mechanism of action. Although further studies would be useful, it has already been shown that **2g** induces apoptosis via the intrinsic signalling pathway (Figure 5). The proapoptotic protein Bcl-2 and the effector caspase-3 are important for the mechanism of apoptosis via **2g** (Figure 6A,B).

Dihydroxyquingdainone **2g** was also convincing because it overcame various cytostatic resistances. In BeKa cells (daunorubicin-resistant Nalm-6 cells), **2g** induced apoptosis as high as in original Nalm-6 cells (Figure 8A). Knowing the mechanism of resistance in these cells helps us to understand how **2g** works. Overcoming this resistance based on the overexpression of p-glycoprotein as a multidrug resistance mechanism shows the importance of further investigation. Furthermore, **2g** is able to overcome etoposide and methotrexate resistance in leukaemia cells (Figure 8B,C). Amounts of 22.7 nM vincristine, 50 nM methotrexate and 0.37 μM etoposide are equivalent to the effect of about 7.5 μM **2g**, which sets a base for further investigation. Closely related to quingdainone is indirubin. Of its derivatives, indirubin-5-sulfonate and indirubin-3’-oxime were recognised as potent anti-leukaemic agents. Due to the large aromatic residue joint to the indole nucleus down side from the double bond, it is hard to see a connection between the activity of quingdainones and indirubin. Indirubins are cell cycle inhibitors [1] by inhibition of CDKs: indirubin-5-sulfonate 3’-oxime has an IC_50_ of 7 nM at CDK-1. Moreover, halogen substituents improve the activity of indirubin in pos. 3 [36]. In our case, the extrinsic and intrinsic apoptosis induction pathways [21,22] play a major role, together with change of the mitochondrial membrane potential. Caspase-3 is processed actively, which then initiates cellular changes of apoptosis [23,24]. We expect a quite different dependence from substituents.

## 4. Materials and Methods

### 4.1. Synthesis of Quingdainone Derivatives

#### 4.1.1. Synthetic Procedures

##### General Considerations

All the commercially available chemicals were used without further purification. NMR spectra were recorded on a JEOL ECS-400 (400 MHz, Japan). Chemical shifts are reported in ppm and refer to residual protons in the deuterated solvent as the internal standard. IR spectra were recorded on a Nicolet 380 FT-IR (Thermo Scientific, England). UV/VIS-spectra were recorded on a Specord 210 (Analytik Jena, Jena, Germany). Mass spectrometry was performed on an IT-TOF from Shimadzu, Japan with direct injection of the samples. For the original NMR, IR and mass spectrometry of compounds, please see the Supplementary Materials. Flash purification was performed with a Puriflash 4250-250 from Interchim, France.

Tryptanthrins **4**, which are initially needed, were prepared with isatin **5** and isatoic anhydride according to Bergman et al. [37], which is extendable to all commercially available isatins. For tryptanthrin derivatives with substituents at positions 1–4 (R’ 2’-5’ in quingdainones), another method was used due to the unavailability of various isatoic acids. 2-Chloroisatins 6 were prepared according to Grimshaw et al. [38]. These 2-chloroisatins reacted with 2-aminobenzoic acids at 130 °C in DMF to form tryptanthrins without any further additives [39]. Unsubstituted quingdainone was obtained according to [12].

##### General Procedure for the Synthesis of Indolo[2,1-b]quinazoline-6,12-diones (Tryptanthrins)

In a 100 mL round bottom flask, 0.2 mL N-methylpiperidine, 5 mL N,N’-diisopropylcarbodiimide and 35 mL pyridine were heated to 65 °C. To this solution was added isatin (0.05 mol, 1 eq.) and isatoic anhydride (8.15 g, 0.05 mol, 1 eq.) under stirring. The mixture was heated to 115 °C under reflux. After approximately one hour, precipitation of the product occurred. The reaction was completed by further heating under reflux for 30 min. The product was isolated by vacuum filtration and washing with methanol. In addition to the works of Bergman [3,37], data about the synthesis of various tryptanthrins can be found in [39,40].

##### Indolo[2,1-b]quinazoline-6,12-dione (**4a**)

In total, 7.35 g of isatin was converted according to the general procedure. Yield: 6.09 g (25 mmol, 50%), intense yellow needles. IR (KBr): 3062, 1721, 1645, 1435, 1256, 804 and 747 cm^−1^. ^1^H NMR (CDCl_3_): δ = 8.61 (d, *J* = 3.4 Hz, ^1^H), 8.44 (d, *J* = 1.17 Hz, 1H), 8.06(d, *J* = 8.01 Hz,1H), 7.94–7.80 (m, 3H), 7.69 (t, *J* = 7.08 Hz, 1H) and 7.28 (t, *J* = 7.44 Hz, 1H). ^13^C NMR (CDCl_3_): δ = 117.5, 120.6, 125.4, 126.3, 127.1, 129.7, 130.0, 133.2, 134.6, 145.3, 146.6, 160.4 and 183.8.

##### 8-Chloroindolo[2,1-b]quinazoline-6,12-dione (**4b**)

In total, 9.35 g of 5-chloroisatin was converted according to the general procedure. Yield: 6.65 g (24 mmol, 45%), intense yellow powder. IR (KBr): 3128w, 3071m, 1731s, 1673s, 1596m, 1461m, 1342m, 1301m, 1126m, 1043w, 878w, 772m and 556w cm^−1^. ^1^H NMR (DMSO-d_6_): δ = 8.44 (d, *J* = 8.7 Hz, 1H), 8.30 (d, *J* = 7.0 Hz, 1H), 7.98–7.91 (m, 3H), 7.90–7.86 (dd, *J* = 8.4, 2.2 Hz, 1H) and 7.72 (dt, *J* = 8.3, 4.2 Hz, 1H). ^13^C NMR (DMSO-d_6_): δ = 181.86, 158.17, 146.92, 145.50, 144.97, 137.36, 135.88, 131.74, 130.58, 130.53, 127.53, 124.83, 124.54, 123.66 and 119.15.

##### 8-Bromoindolo[2,1-b]quinazoline-6,12-dione (**4c**)

In total, 11.30 g of 5-bromoisatin was converted according to the general procedure. Yield: 7.13 g (22 mmol, 44%), intense yellow powder. IR (KBr): 3126w, 3069m, 1731s, 1671s, 1592s, 1460s, 1339s, 1300s, 1127m, 1042m, 841m, 772s, 688m, 616w and 475m cm^−1^. ^1^H NMR (DMSO-d_6_): δ = 8.38 (d, *J* = 8.4 Hz, 1H), 8.29 (d, *J* = 7.8 Hz, 1H), 8.06–7.98 (m, 2H), 7.92 (d, *J* = 3.7 Hz, 2H) and 7.72 (dt, *J* = 8.2, 4.2 Hz, 1H). ^13^C NMR (DMSO-d_6_): δ = 181.74, 158.17, 146.93, 145.33, 140.23, 135.89, 130.58, 130.52, 127.64, 127.53, 124.80, 123.66, 119.66 and 119.47.

##### 8-Methylindolo[2,1-b]quinazoline-6,12-dione (**4d**)

In total, 8.06 g of 5-methylisatin was converted according to the general procedure. Yield: 6.65 g (25 mmol, 50%), intense yellow powder. IR (KBr): 3063w, 3031w, 1726s, 1687s, 1594m, 1484s, 1459m, 1484m, 1459m, 1341s, 1309s, 1297m, 1228m, 1139m, 1042m, 887w, 775s, 688m and 476w cm^−1^. ^1^H NMR (DMSO-d_6_): δ = 8.31 (d, *J* = 8.2 Hz, 1H), 8.28 (dt, *J* = 7.9, 1.1 Hz, 1H), 7.91 (d, *J* = 1.0 Hz, 1H), 7.90 (t, *J* = 1.1 Hz, 1H), 7.73–7.67 (m, 1H), 7.67–7.61 (m, 2H) and 2.37 (s, 3H). ^13^C NMR (DMSO-d_6_): δ = 183.06, 158.06, 147.02, 145.76, 144.51, 138.65, 137.20, 135.59, 130.42, 130.33, 127.41, 125.30, 123.89, 122.84, 117.33 and 20.96.

##### 8-Bromo-6,12-dioxo-6,12-dihydroindolo[2,1-b]quinazoline-3-carboxylic Acid (**4e**)

5-bromoisatin (2.26 g, 10 mmol, 1 eq.) and PCl_5_ (2.5 g, 12 mmol, 1.2 eq.) were dissolved in 20 mL of dry benzene and heated to reflux for 2 h. The mixture was cooled to ca. 30 °C and the solvent was removed in vacuo. The remaining solid was dissolved in 17 mL of dry DMF followed by the addition of 2-aminoterephthalic acid (1.81 g, 10 mmol, 1 eq.). The solution was heated to 120 °C with an attached reflux condenser for 15 min. The product was isolated by vacuum filtration and washed with benzene and acetone. Yield (over 2 steps): 1.98 g (5.34 mmol, 53%), green-yellow powder. IR (KBr): 3085w, 3061w, 3028w, 1702s, 1679s, 1588s, 1547m, 1460s, 1428m, 1344m, 1301s, 1257m, 1215m, 1183m, 1117w, 1039w, 857m, 846m, 791m, 746m, 714w, 624w and 492m cm^−1^. ^1^H NMR (DMSO-d_6_): δ = 8.42–8.29 (m, 2H), 8.10 (dd, *J* = 8.7, 2.3 Hz, 1H), 8.07–7.98 (m, 2H) and 7.87 (s, 1H). ^13^C NMR (DMSO-d_6_): δ = 180.92, 156.49, 145.49, 145.40, 145.10, 144.52, 139.79, 138.20, 132.04, 129.17, 127.21, 124.74, 124.25, 123.02, 119.42 and 118.97.

##### 8-methoxyindolo[2,1-b]quinazoline-6,12-dione (**4f**)

In total, 8.86 g of 5-methoxylisatin was converted according to the general procedure. Yield: 7.67 g (28 mmol, 56%), intense orange needles. ^1^H NMR (DMSO-d_6_): δ = 8.36 (d, *J* = 8.7 Hz, 1H), 8.29 (d, *J* = 7.9 Hz, 1H), 7.92 (d, *J* = 3.7 Hz, 2H), 7.73 (dq, *J* = 8.2, 4.4 Hz, 1H), 7.45–7.36 (m, 2H) and 3.86 (s, 3H). ^13^C NMR (DMSO-d_6_): δ = 182.35, 157.98, 157.28, 146.42, 145.37, 139.91, 134.96, 129.88, 129.81, 126.79, 123.73, 123.40, 118.23, 108.47, 99.46 and 56.03.

##### General Procedure for the Synthesis of 5-(12-Oxoindolo[2,1-b]quinazolin-6(12H)-ylidene)-2-thioxothiazolidin-4-ones

In a 100 mL round bottomed flask, one of the synthesised tryptanthrins (9.91 mmol, 1 eq.) and 2-thioxothiazolidin-4-one (1.46 g, 10.96 mmol, 1.1 eq.) were suspended in 30 mL of acetic acid. Subsequently, 10 mL of BF_3_∙HOAc was added and the mixture was heated to reflux for 7 h. After cooling to RT, the product was isolated by vacuum filtration and washing with 60 mL of AcOH and 40 mL of H_2_O.

##### 5-(12-Oxoindolo[2,1-b]quinazolin-6(12H)-ylidene)-2-thioxothiazolidin-4-one (**7a**)

In total, 2.48 g of tryptanthrin was converted according to the general procedure. Yield: 3.7 g (10 mmol, quant.), dark red powder. IR (KBr): 3258m(br), 3121w, 3097w, 1720m, 1654s, 1553s, 1435m, 1345m, 1297w, 1218m, 1161w, 1065s, 1030m, 842w, 756m, 681w, 667w, 595w and 543w cm^−1^. ^1^H NMR (DMSO-d_6_): δ = 9.27 (d, *J* = 8.1 Hz, 1H), 8.57 (d, *J* = 7.9 Hz, 1H), 8.32 (dd, *J* = 8.0, 1.3 Hz, 1H), 8.03–7.90 (m, 2H), 7.70 (q, *J* = 7.8 Hz, 2H) and 7.50 (t, *J* = 7.8 Hz, 1H).

##### 5-(8-Chloro-12-oxoindolo[2,1-b]quinazolin-6(12H)-ylidene)-2-thioxothiazolidin-4-one (**7b**)

In total, 2.8 g of **4b** was converted according to the general procedure. Yield: 3.72 g (9.37 mmol, 95%), red solid. IR (KBr): 3066 m(br), 2859m, 1713s, 1663s, 1619s, 1595s, 1446s, 1379m, 1322s, 1285w, 1235s, 1154w, 1098m, 962s, 898w, 860w, 767s, 730m, 684m, 667s, and 581s cm^−1^. ^1^H NMR (DMSO-d_6_): δ = 9.30 (d, *J* = 2.2 Hz, 1H), 8.54 (d, *J* = 8.7 Hz, 1H), 8.31 (dd, *J* = 7.7, 1.1 Hz, 1H), 8.03–7.87 (m, 2H), 7.76 (dd, *J* = 8.6, 2.3 Hz, 1H) and 7.68 (t, *J* = 7.6 Hz, 1H).

##### 5-(8-Bromo-12-oxoindolo[2,1-b]quinazolin-6(12H)-ylidene)-2-thioxothiazolidin-4-one (**7c**)

In total, 3.42 g of **4c** was converted according to the general procedure. Yield: 4.08 g (9.22 mmol, 93%), red powder. IR (KBr): 3094m(br), 2861m, 1715s, 1665s, 1621s, 1452s, 1347s, 1323m, 1295w, 1274w, 1237m, 1177m, 1154w, 1095w, 1075w, 1010w, 963w, 898w, 823w, 791w, 770m, 720w, 686m, 667w, 590m and 542m cm^−1^. ^1^H-NMR (DMSO-d_6_): δ = 9.47 (d, *J* = 2.0 Hz, 1H), 8.50 (d, *J* = 8.7 Hz, 1H), 8.32 (dd, *J* = 8.1, 1.2 Hz, 1H), 8.01–7.93 (m, 2H), 7.90 (dd, *J* = 8.6, 2.0 Hz, 1H) and 7.70 (td, *J* = 7.4, 1.4 Hz, 1H).

##### 5-(8-Methyl-12-oxoindolo[2,1-b]quinazolin-6(12H)-ylidene)-2-thioxothiazolidin-4-one (**7d**)

In total, 2.62 g of **4d** was converted according to the general procedure. Yield: 4.05 g (10.73 mmol, quant.), dark red powder. IR (KBr): 3107m(br), 2880m, 1723s, 1693s, 1659s, 1607w, 1560m, 1476s, 1452s, 1376w, 1331s, 1312m, 1270w, 1245w, 1219s, 1162w, 1074s, 1032m, 1032m, 994m, 889w, 833w, 801w, 767m, 704w, 681m, 601m, 546m and 524w cm^−1^. ^1^H NMR (DMSO-d_6_): δ = 9.08 (s, 1H), 8.42 (d, *J* = 8.2 Hz, 1H), 8.30 (d, *J* = 7.6 Hz, 1H), 8.01–7.88 (m, 2H), 7.69 (d, *J* = 8.2 Hz, 1H), 7.51 (d, *J* = 8.3 Hz, 1H) and 2.45 (s, 3H).

##### 8-Bromo-12-oxo-6-(4-oxo-2-thioxothiazolidin-5-ylidene)-6,12-dihydroindolo[2,1-b]quinazoline-3-carboxylic Acid (**7e**)

In total, 3.42 g of **4e** was converted according to the general procedure. Another 20 mL of AcOH was added because the suspension was hard to stir. Yield: 3.76 g (7.73 mmol, 84%), dark red powder.

IR (KBr): 3178m(br), 3124m, 3063m, 2861w(br), 1704s, 1682s, 1611m, 1594m, 1449m, 1430m, 1351m, 1322m, 1299m, 1255m, 1201m, 1178s, 1118w, 1103w, 1089w, 1077w, 1016w, 977w, 952w, 934w, 912w, 855w, 829m, 760m, 702w, 684w, 665w, 577w, 554w and 542w cm^−1^. ^1^H NMR (DMSO-d_6_): δ = 9.30 (d, *J* = 2.2 Hz, 1H), 8.41–8.37 (m, 1H), 8.34–8.31 (m, 1H), 8.27 (d, *J* = 8.2 Hz, 1H), 8.21 (d, *J* = 1.6 Hz, 1H), 8.18–8.14 (m, 0.7H), 8.07–8.00 (m, 2.5H) and 7.79 (dd, *J* = 8.7, 2.2 Hz, 1H).

##### 5-(8-Methoxy-12-oxoindolo[2,1-b]quinazolin-6(12H)-ylidene)-2-thioxothiazolidin-4-one (**7f**)

In total, 2.02 g of **4f** was converted according to the general procedure. Yield: 2.29 g (5.82 mmol, 80%), black powder (2:1 (Z)/(E)-mixture). ^1^H NMR (DMSO-d_6_): δ = 8.84 (d, *J* = 2.7 Hz, 1H, major isomer), 8.38 (d, *J* = 8.7 Hz, 3H, minor isomer), 8.31 (d, *J* = 7.3 Hz, 1H, minor isomer), 8.25 (d, *J* = 7.8 Hz, 1H, major isomer), 7.99–7.82 (m, 3H, major isomer), 7.73 (dt, *J* = 8.2, 4.4 Hz, 1H, minor isomer), 7.69–7.61 (m, 1H, major isomer), 7.45–7.38 (m, 2H, minor isomer), 7.22 (dd, *J* = 8.9, 3.0 Hz, 1H, major isomer), 3.87 (s, 3H, minor isomer) and 3.85 (s, 3H, major isomer).

##### General Procedure for the Synthesis of 2-Mercapto-2-(12-oxoindolo[2,1-b]quinazolin-6(12H)-ylidene)acetic Acids

In a 100 mL round-bottomed flask, KOH (3 g, 53.47 mmol) was dissolved in 42 mL of H_2_O. In total, 1.5 g of substances **7a–f** was added, and the suspension was heated to 60 °C. At this temperature, 17 mL of ethanol was added via the reflux condenser. The mixture was heated further to 80 °C under reflux for 3 h. After cooling to RT, the resulting suspension was filtered. An intense violet substance formed, which was collected in the filter. The filtrate was acidified dropwise at 0 °C with 17% aqueous HCl. At ca. pH 6–7, the colour changed from dark green to light brown and a brown solid precipitate formed. This brown material was removed. The suspension was filtered again, and the filtrate was further acidified dropwise at 0 °C until pH 1. The precipitated product was isolated by vacuum filtration and washed with water.

##### 2-Mercapto-2-(12-oxoindolo[2,1-b]quinazolin-6(12H)-ylidene)acetic Acid (**8a**)

The **7a** (1.5 g, 4.13 mmol) was converted according to the general procedure. Yield: 1.16 g (3.6 mmol, 88%), light brown solid. The obtained substance was not analytically pure; therefore, it was allylated to a thioether.

##### 2-(Allylthio)-2-(12-oxoindolo[2,1-b]quinazolin-6(12H)-ylidene)acetic Acid (**8a’**)

The **8a** (620 mg, 1.92 mmol, 1 eq.) was dissolved in 5 mL EtOH. Allyl bromide (1 mL, 11.57 mmol, 6.03 eq.) and a solution of KOH (225 mg, 4 mmol, 2.1 eq.) in 5 mL of MeOH was added to this mixture. After stirring for 1 h, a yellow solid was isolated by filtration and washing with EtOH. Yield: 450 mg (1.24 mmol, 65%), dark yellow powder. IR (KBr): 3398m(br), 3066w, 2977w, 2917w, 1672m, 1606s, 1554s, 1468m, 1370m, 1328w, 1269m, 1228w, 1115w, 1080w, 1029w, 924w, 877w, 768m, 743w, 691w, 681w, 614w and 561w cm^−1^. ^1^H NMR (DMSO-d_6_): δ = 8.68–8.47 (m, 1H), 8.23 (td, *J* = 8.8, 1.5 Hz, 1H), 8.12–7.91 (m, 1H), 7.82–7.75 (m, 1H), 7.65 (dd, *J* = 34.5, 7.7 Hz, 1H), 7.48–7.41 (m, 1H), 7.38 (dd, *J* = 5.9, 3.2 Hz, 1H), 7.34–7.22 (m, 1H), 6.12–5.98 (m, 1H), 5.38–5.25 (m, 1H), 5.19–5.08 (m, 1H), 3.82 (d, *J* = 7.2 Hz, 1H) and 3.70 (d, *J* = 7.3 Hz, 1H).

##### 2-(8-Chloro-12-oxoindolo[2,1-b]quinazolin-6(12H)-ylidene)-2-mercaptoacetic Acid (**8b**)

The **7b** (1.5 g, 3.77 mmol) was converted according to the general procedure. Yield: 850 mg (2.38 mmol, 45%), dark orange solid. IR (KBr): 3399m(br), 3077m, 2928w, 2553w(br), 1685s, 1624s, 1582s, 1512s, 1454w, 1310w, 1241m, 1077m, 935w, 798w and 747w cm^−1^. ^1^H NMR (DMSO-d_6_): δ = 12.85 (s, 1H), 12.16 (s, 1H), 7.98 (dd, *J* = 7.8, 1.5 Hz, 1H), 7.78 (d, J = 7.8 Hz, 1H), 7.71 (t, *J* = 7.7 Hz, 1H), 7.41 (t, *J* = 7.5 Hz, 1H), 7.33 (s, 1H), 7.20 (d, *J* = 8.2 Hz, 1H) and 7.17 (dd, *J* = 8.4, 1.8 Hz, 1H). ^13^C NMR (DMSO-d_6_): δ = 193.11, 169.88, 167.18, 154.29, 136.97, 134.43, 133.41, 131.96, 127.34, 126.70, 125.05, 124.76, 124.57, 124.14, 118.37, 113.54 and 111.02. HRMS-TOF (*m*/*z*): 354.9946 ([M–H]^−^, calc.: 354.9950, diff.: 1.13 ppm).

##### 2-(8-Bromo-12-oxoindolo[2,1-b]quinazolin-6(12H)-ylidene)-2-mercaptoacetic Acid (**8c**)

The **7c** (1.5 g, 3.39 mmol) was converted according to the general procedure. Yield: 906 mg (2.26 mmol, 66%), dark orange solid. IR (KBr): 3430m(br), 3104w, 3069w, 2933w, 1701m, 1629s, 1565s, 1505s, 1337w, 1302m, 1236w, 1217w, 1090m, 935w, 878w, 752m, 682w, 643w and 599w cm^−1^. ^1^H NMR (DMSO-d_6_): δ = 12.20 (s, 1H), 8.02 (d, *J* = 7.8 Hz, 1H), 7.81 (d, *J* = 8.0 Hz, 1H), 7.75 (t, *J* = 7.8 Hz, 1H), 7.51 (s, 1H), 7.45 (t, *J* = 7.5 Hz, 1H), 7.33 (d, *J* = 7.7 Hz, 1H) and 7.18 (d, *J* = 8.3 Hz, 1H).

##### 2-Mercapto-2-(8-methyl-12-oxoindolo[2,1-b]quinazolin-6(12H)-ylidene)acetic Acid (**8d**)

The **7d** (1.5 g, 3.97 mmol) was converted according to the general procedure. Yield: 984 mg (2.93 mmol, 74%), dark red solid. The obtained substance was not pure enough for analytics, and therefore it was allylated to a thioether.

##### 2-(Allylthio)-2-(8-methyl-12-oxoindolo[2,1-b]quinazolin-6(12H)-ylidene)acetic Acid (**8d’**)

The **8d** (620 mg, 1.84 mmol, 1 eq.) was dissolved in 5 mL EtOH. To this mixture was added allyl bromide (1 mL, 11.57 mmol, 6.29 eq.) and a solution of KOH (225 mg, 4 mmol, 2.17 eq.) in 5 mL MeOH was added to this mixture. After stirring for 1 h, a yellow solid was isolated by filtration and washing with EtOH. Yield: 335 mg (0.89 mmol, 48%), dark yellow powder. 

IR (KBr): 3423m(br), 3075w, 2974w, 2917w, 2860w, 1673s, 1606s, 1552s, 1507m, 1468s, 1354s, 1318m, 1270m, 1229m, 1203m, 1115w, 1085m, 1015w, 952w, 923w, 891w, 815m, 766m, 704m, 692m, 611m, 589w and 529w cm^−1^. ^1^H NMR (DMSO-d_6_): δ = 8.43 (dd, *J* = 43.4, 8.2 Hz, 1H), 8.22 (t, *J* = 8.8 Hz, 1H), 7.83 (s, 1H), 7.81–7.73 (m, 1H), 7.63 (dd, *J* = 41.4, 8.3 Hz, 1H), 7.43 (q, *J* = 7.8 Hz, 1H), 7.16 (dd, *J* = 27.6, 8.7 Hz, 1H), 6.05 (ddt, *J* = 17.3, 9.8, 7.4 Hz, 1H), 5.37–5.24 (m, 1H), 5.17–5.08 (m, 1H), 3.82 (d, *J* = 7.3 Hz, 1H), 3.70 (d, *J* = 7.4 Hz, 1H) and 2.34 (s, 3H).

##### 8-Bromo-6-(carboxy(mercapto)methylene)-12-oxo-6,12-dihydroindolo[2,1-b]quinazoline-3-carboxylic Acid (**8e**)

The **7e** (1.5 g, 3.08 mmol) was converted according to the general procedure. Yield: 995 mg (2.24 mmol, 73%), dark brown solid. The obtained substance was not pure enough for analytics, and therefore it was allylated to a thioether.

##### 6-((Allylthio)(carboxy)methylene)-8-bromo-12-oxo-6,12-dihydroindolo[2,1-b]quinazoline-3-carboxylic Acid (**8e’**)

The **8e** (109 mg, 0.25 mmol, 1 eq.) was dissolved in 1 mL of EtOH. To this mixture was added allyl bromide (200 µL, 2.31 mmol, 9.26 eq.) and a solution of KOH (45 mg, 0.8 mmol, 3.2 eq.) in 1 mL of MeOH. After stirring for 1 h, a yellow solid was isolated by filtration and washing with EtOH. Yield: 43 mg (0.09 mmol, 37%), dark yellow powder. IR (KBr): 3420s(br), 2923w, 1675m, 1618s, 1561s, 1539s, 1501m, 1384s, 1346s, 1257w, 1222w, 1190w, 1138w, 1089w, 1067w, 1019w, 919w, 804w, 771m, 759m, 735w, 701w, 688w, 663w, 652w, 645w, 639w, 607w, 582w, 535w and 512w cm^−1^. ^1^H NMR (DMSO-d_6_): δ = 8.54 (d, *J* = 8.4 Hz, 1H), 8.11–8.03 (m, 2H), 7.83 (d, *J* = 6.8 Hz, 1H), 7.55 (dd, *J* = 8.5, 2.1 Hz, 1H), 6.06 (ddt, *J* = 17.0, 9.7, 7.2 Hz, 1H), 5.41–5.26 (m, 1H), 5.14 (dd, *J* = 9.9, 1.9 Hz, 1H) and 3.77 (dd, *J* = 55.4, 7.3 Hz, 2H).

##### 2-Mercapto-2-(8-methoxy-12-oxoindolo[2,1-b]quinazolin-6(12H)-ylidene)acetic Acid (**8f**)

The **7f** (1.54 g, 3.91 mmol) was converted according to the general procedure. The product was used without any further purification for the following step. Yield: 692 mg (1.96 mmol, 50%), orange powder.

##### General Procedure for the Synthesis of 2-(12-Oxoindolo[2,1-b]quinazolin-6(12H)-ylidene)-2-(phenylamino)acetic Acids

In total, 400 mg of substances **8b–f** was added to 2 g of a para-substituted-aniline in a 100 mL Erlenmeyer flask with a ground glass joint. This mixture was heated to 100 °C for 1 h under stirring. The aniline, which deposits on the edge of the vessel, was brought back to the medium with a spatula every 15 min. After cooling to 80 °C, the excess aniline was removed by sublimation at 80 °C and 7 mbar for 1 h. The remaining solid was stirred with 10 mL of 10% NaOH solution. After 1 h, the suspension was filtrated and the residue was allowed to dry overnight. The resulting solid was again sublimated at 140 °C at 10^−3^ mbar to remove the rest of the aniline, while the product remained at the bottom of the flask.

##### 2-(8-Chloro-12-oxoindolo[2,1-b]quinazolin-6(12H)-ylidene)-2-(p-tolylamino)acetic Acid (**9b**)

The **8b** (400 mg, 1.12 mmol, 1 eq.) was converted with p-toluidine (2 g, 18.67 mmol, 16.65 eq.) according to the general procedure. Yield: 377 mg (0.88 mmol, 78%), yellow powder. IR (KBr): 3623w, 3411m(br), 3205w, 3022w, 2917w, 2857w, 1619s, 1603s, 1556s, 1518m, 1492m, 1467s, 1403s, 1359w, 1312w, 1267m, 1226m, 1147w, 1134w, 1081w, 1014w, 881m, 846w, 805m, 760m, 688m, 643w, 564w, 495w, 457w and 429w cm^−1^. ^1^H NMR (DMSO-d_6_): δ = 8.54 (d, *J* = 8.5 Hz, 1H), 8.25 (d, *J* = 8.0 Hz, 1H), 7.83 (d, *J* = 2.1 Hz, 1H), 7.77 (d, *J* = 3.6 Hz, 2H), 7.50 (d, *J* = 8.3 Hz, 2H), 7.36 (dt, *J* = 8.1, 4.0 Hz, 1H), 7.26–7.14 (m, 3H) and 2.29 (s, 3H).

##### 2-(8-Bromo-12-oxoindolo[2,1-b]quinazolin-6(12H)-ylidene)-2-(p-tolylamino)acetic Acid (**9c**)

The **8c** (400 mg, 1 mmol, 1 eq.) was converted with p-toluidine (2 g, 18.67 mmol, 18.67 eq.) according to the general procedure. Yield: 312 mg (0.66 mmol, 66%). IR (KBr): 3417m(br), 3060w, 3030w, 2917w, 1622s, 1569s, 1486s, 1470s, 1413s, 1397s, 1356m, 1318m, 1268s, 1231m, 1169w, 1094m, 1060w, 1038w, 1022w, 1010w, 908w, 896w, 850w, 816m, 766m, 715w, 690m, 665w, 612w, 592w, 561w and 501w cm^−1^. ^1^H NMR (DMSO-d_6_): δ = 8.53 (d, *J* = 8.5 Hz, 1H, 3), 8.29 (d, *J* = 7.9 Hz, 1H, 38), 8.02 (d, *J* = 2.0 Hz, 1H), 7.81 (dd, *J* = 4.3, 1.0 Hz, 2H), 7.55 (d, *J* = 8.4 Hz, 2H), 7.46–7.35 (m, 2H), 7.24 (d, *J* = 8.1 Hz, 2H) and 2.34 (s, 3H). ^13^C NMR (DMSO-d_6_): δ = 163.27, 159.13, 157.80, 152.90, 147.67, 136.51, 134.10, 134.01, 131.53, 129.76, 129.17, 126.42, 125.63, 124.56, 123.52, 120.97, 120.09, 117.98, 117.30, 117.00, 88.95 and 20.50. HRMS-TOF (*m*/*z*): 472.0298 ([M–H]^−^, calc.: 472.0302, diff.: 0.85 ppm).

##### 2-(8-Methyl-12-oxoindolo[2,1-b]quinazolin-6(12H)-ylidene)-2-(p-tolylamino)acetic Acid (**9d**)

The **8d** (400 mg, 1.19 mmol, 1 eq.) was converted with p-toluidine (2 g, 18.67 mmol, 15.69 eq.) according to the general procedure. Yield: 300 mg (0.75 mmol, 63%). IR (KBr): 3428w(br), 3023w, 2919w, 2860w, 1607s, 1587s, 1470s, 1402s, 1317w, 1269m, 1231m, 1148w, 1097w, 1115w, 1022w, 896w, 852w, 814w, 793w, 762w, 711w, 689w, 623w, 589w and 497w cm^−1^. ^1^H NMR (DMSO-d_6_): δ = 8.47 (d, *J* = 8.2 Hz, 1H), 8.32–8.23 (m, 1H), 7.79 (d, *J* = 1.3 Hz, 1H), 7.78 (d, *J* = 1.0 Hz, 1H), 7.74–7.67 (m, 1H), 7.54 (d, *J* = 8.4 Hz, 2H), 7.44–7.33 (m, 1H), 7.21 (d, *J* = 8.2 Hz, 2H), 7.04 (dd, *J* = 8.3, 1.7 Hz, 1H), 2.40 (s, 3H) and 2.32 (s, 3H). ^13^C NMR (DMSO-d_6_): δ = 163.68, 159.03, 157.25, 153.28, 147.63, 136.86, 133.83, 133.47, 130.69, 129.69, 126.97, 126.36, 125.55, 123.26, 123.11, 119.86, 119.20, 117.34, 115.04, 89.72, 21.61 and 20.48. HRMS-TOF (*m*/*z*): 408.1334 ([M–H]^−^, calc.: 408.1354, diff.: 4.9 ppm).

##### 2-((4-Chlorophenyl)amino)-2-(8-methyl-12-oxoindolo[2,1-b]quinazolin-6(12H)-ylidene)acetic Acid (**9e**)

The **8d** (400 mg, 1.19 mmol, 1 eq.) was converted with 4-chloroaniline (2 g, 15.68 mmol, 13.18 eq.) according to the general procedure. Yield: 366 mg (0.85 mmol, 72%), dark yellow powder. IR (KBr): 3417m(br), 3060w, 3030w, 2917w, 1622s, 1569s, 1486s, 1470s, 1413s, 1397s, 1356m, 1318m, 1268s, 1231m, 1169w, 1094m, 1060w, 1038w, 1022w, 1010w, 908w, 896w, 850w, 816m, 766m, 715w, 690m, 665w, 612w, 592w, 561w and 501w cm^−1^. ^1^H NMR (DMSO-d_6_): δ = 8.47 (d, *J* = 8.2 Hz, 1H), 8.29 (dd, *J* = 7.6, 1.0 Hz, 1H), 7.87–7.76 (m, 2H), 7.73 (s, 1H), 7.68 (d, *J* = 8.7 Hz, 2H), 7.46 (d, *J* = 8.8 Hz, 2H), 7.41 (ddd, *J* = 8.2, 6.4, 2.1 Hz, 1H), 7.07 (dd, *J* = 8.3, 1.8 Hz, 1H) and 2.40 (s, 3H). ^13^C NMR (DMSO-d_6_): δ = 163.30, 158.75, 156.32, 153.19, 147.23, 138.17, 133.78, 133.68, 130.83, 128.94, 127.78, 126.50, 126.15, 125.50, 123.42, 123.38, 121.20, 119.22, 117.40, 114.88, 90.43 and 21.38. HRMS-TOF (m/z): 428.0808 ([M–H]^−^, calc.: 428.0807, diff.: 0.23 ppm).

##### 8-Bromo-6-(carboxy(p-tolylamino)methylene)-12-oxo-6,12-dihydroindolo[2,1-b]quinazoline-3-carboxylic Acid (**9f**)

The **8e** (400 mg, 0.9 mmol, 1 eq.) was converted with p-toluidine (2 g, 18.67 mmol, 20.74 eq.) according to the general procedure. Yield: 255 mg (0.24 mmol, 55%). IR (KBr): 3424s(br), 3022w, 2917w, 1619s, 1598s, 1574m, 1543m, 1438s, 1403s, 1283m, 1261m, 1230m, 1049w, 878w, 846, 805, 774, 691w, 666w, 619w and 593w cm^−1^. ^1^H NMR (DMSO-d_6_): δ = 12.86 (s, 1H), 8.53 (d, *J* = 8.5 Hz, 1H), 8.16 (d, *J* = 8.2 Hz, 1H), 8.09 (d, *J* = 1.3 Hz, 1H), 8.01 (d, *J* = 2.2 Hz, 1H), 7.84 (dd, *J* = 8.2, 1.4 Hz, 1H), 7.52 (d, *J* = 8.3 Hz, 2H), 7.37 (dd, *J* = 8.5, 2.0 Hz, 1H), 7.26 (d, *J* = 8.2 Hz, 2H) and 2.33 (s, 3H). ^13^C NMR (DMSO-d_6_): δ = 168.99, 163.43, 159.42, 157.80, 153.04, 147.56, 146.58, 136.84, 134.08, 131.83, 130.06, 129.43, 125.86, 125.48, 124.83, 124.70, 121.16, 120.12, 118.10, 117.48, 117.17, 89.14 and 20.72.

##### 2-(8-Methoxy-12-oxoindolo[2,1-b]quinazolin-6(12H)-ylidene)-2-((4-methoxyphenyl)amino)acetic Acid (**9g**)

The **8f** (333 mg, 0.95 mmol, 1 eq.) was converted with freshly sublimed p-anisidine (2 g, 16.24 mmol, 17.09 eq.) according to the general procedure. Yield: 330 mg (0.75 mmol, 79%). ^1^H NMR (DMSO-d_6_): δ = 8.47 (d, *J* = 8.7 Hz, 1H), 8.25 (d, *J* = 7.8 Hz, 1H), 7.76 (d, *J* = 3.2 Hz, 2H), 7.57 (d, *J* = 9.2 Hz, 2H), 7.47 (d, *J* = 2.7 Hz, 1H), 7.41–7.29 (m, 1H), 6.97 (d, *J* = 9.2 Hz, 2H), 6.80 (dd, *J* = 8.7, 2.7 Hz, 1H), 3.78 (s, 3H) and 3.78 (s, 3H). ^13^C NMR (DMSO-d_6_): δ = 163.51, 158.99, 157.99, 157.30, 156.61, 153.55, 147.80, 133.85, 132.62, 128.63, 127.03, 126.49, 125.72, 123.27, 121.74, 117.44, 116.06, 114.68, 107.79, 104.80 and 55.58.

##### General Procedure for the Synthesis of (Z)-6-(3-Oxoindolin-2-ylidene)indolo[2,1-b]quinazolin-12(6H)-ones (Quingdainones)

Substances **9b–f** were stirred in 15–30 g of polyphosphoric acid (PPA) at 115 °C for 6 h. The hot mixture was poured into an Erlenmeyer flask containing crushed ice. After stirring the resulting suspension for 1 h, the solid was isolated by filtration and washed with water.

##### (Z)-6-(5-Methyl-3-oxoindolin-2-ylidene)indolo[2,1-b]quinazolin-12(6H)-one (**2a**)

In total, 400 mg (1.24 mmol, 1 eq.) of **8a** was stirred with 1.5 g (14 mmol, 11.29 g eq.) of *p*-toluidine at 100 °C for 1 h. Excess p-toluidine was removed by sublimation with a Glasofen Kugelrohr apparatus (Büchi) at 90 °C and 7 mbar for 1 h. Without further work-up, the crude material was treated with 11 g of PPA at 120 °C for 2 h. The mixture was subsequently poured into crushed ice and stirred for 1 h. The resulting suspension was filtrated and washed with water. The filter paper was extracted with acetone in a Soxhlet apparatus. After 3 h, the receiving flask was changed and the first fraction was discarded. After 3 d, the remaining material was extracted with dimethylacetamide for 4 h. Crystallisation at −15 °C gave 15 mg (0.04 mmol, 3%) from the acetone extract and 25 mg (0.07 mmol, 6%) from the DMA extract. UV/VIS: λ_max_ (CDCl_3_) = 256 (log(*ε*) 1.52), 293 (1.36), 355 (0.88), 546 (1.01) and 587 (1.14) nm. IR (KBr): 3433m(br), 3104w, 2917w, 2850w, 1691s, 1625m, 1612s, 1549m, 1492m, 1467m, 1332s, 1293m, 1261m, 1211m, 1122w, 1055w, 878w, 824m, 770m, 681m, 621w and 596w cm^−1^. ^1^H NMR (CDCl3): δ = 11.69 (s, 1H), 9.29 (d, *J* = 2.2 Hz, 1H), 8.63 (d, *J* = 8.5 Hz, 1H), 8.48–8.41 (m, 1H), 7.82 (d, *J* = 9.0 Hz, 2H), 7.57 (s, 1H), 7.52 (d, *J* = 13.4 Hz, 1H), 7.45 (dd, *J* = 8.5, 2.1 Hz, 1H), 7.38 (d, *J* = 9.1 Hz, 1H), 7.02 (d, *J* = 8.2 Hz, 1H) and 2.39 (s, 3H).

##### (Z)-8-Chloro-6-(5-methyl-3-oxoindolin-2-ylidene)indolo[2,1-b]quinazolin-12(6H)-one (**2b**)

In total, 83 mg (0.20 mmol) of **9b** was converted with 17 g of PPA according to the general procedure. The product was purified by recrystallisation from acetone. Yield: 11 mg (0.027 mmol, 14%), dark purple powder. UV/VIS: λ_max_ (CHCl_3_) = 239 (log(*ε*) 1.36), 293 (1.16), 354 (0.67), 546 (0.84) and 588 (0.97) nm. IR (KBr): 3436m(br), 3113w, 2920w, 2853w, 1685s, 1628s, 1609s, 1588m, 1549s, 1486s 1464s, 1318s, 1296m, 1261s 1207s, 1122s, 1049s, 954w, 875m, 818m, 764m, 685m and 590w cm^−1^. ^1^H NMR (CDCl_3_): δ = 9.26 (s, 1H), 8.62 (d, *J* = 8.6 Hz, 1H), 8.43 (d, *J* = 8.1 Hz, 1H), 7.83 (t, *J* = 7.5 Hz, 2H), 7.53 (d, *J* = 11.4 Hz, 2H), 7.48–7.39 (m, 1H), 7.38 (d, *J* = 8.2 Hz, 1H), 7.01 (d, *J* = 8.2 Hz, 1H) and 2.39 (s, 3H). HRMS-TOF (*m*/*z*): C_24_ H_14_ N_3_ O_2_ Cl, 412.0832 ([M + H]^+^, calc.: 412.0847, diff.: 3.64 ppm).

##### (Z)-8-Bromo-6-(5-methyl-3-oxoindolin-2-ylidene)indolo[2,1-b]quinazolin-12(6H)-one (**2c**)

In total, 150 mg (0.32 mmol) of **9c** was converted with 11 g of PPA according to the general procedure. The filter paper was extracted in a Soxhlet apparatus with acetone. When the solvent around the thimble turned purple (30 min), the receiving flask was changed and the previously collected extract was discarded. The Soxhlet extraction was continued with acetone and chloroform for 6 h. Removal of the solvents in vacuo yielded a purple solid, which was further purified by recrystallization from acetone. Yield: 28 mg (0.06 mmol, 19%). IR (KBr): 3445m(br), 3110w, 3069w, 2920w, 2850w, 1695s, 1628m, 1609s, 1552m, 1489s, 1467m, 1315s, 1261m, 1204m, 1119m, 1049m, 875w, 815m, 767m, 676w, 628w and 587w cm^−1^. ^1^H NMR (DMSO-d_6_): δ = 11.79 (s, 1H), 9.41 (d, *J* = 2.0 Hz, 1H), 8.54 (d, *J* = 8.6 Hz, 1H), 8.33 (dd, *J* = 8.0, 1.6 Hz, 1H), 8.22 (d, *J* = 7.8 Hz, 1H), 7.97 (ddd, *J* = 8.6, 7.1, 1.7 Hz, 1H), 7.75 (dd, *J* = 8.6, 2.0 Hz, 1H), 7.63 (t, *J* = 7.0 Hz, 1H), 7.5–7.46 (m, 3H) and 2.36 (s, 3H).

##### (Z)-8-Methyl-6-(5-methyl-3-oxoindolin-2-ylidene)indolo[2,1-b]quinazolin-12(6H)-one (**2d**)

In total, 65 mg (0.15 mmol) of **9d** was converted with 16 g of PPA according to the general procedure. Yield: 57 mg (0.14 mmol, 88%), intense purple powder. IR (KBr): 3427w(br), 3081w, 3034w, 2920w, 1688s, 1628m, 1609s, 1552m, 1495s, 1463m, 1318s, 1289m, 1261m, 1192m, 1119m, 1052m, 808w, 770m, 688w, 666w and 596w cm^−1^. ^1^H NMR (DMSO-d_6_): δ = 11.74 (s, 1H), 9.04 (s, 1H), 8.46 (d, *J* = 8.0 Hz, 1H), 8.30 (dd, *J* = 7.9, 1.6 Hz, 1H), 8.20 (d, *J* = 8.2 Hz, 1H), 7.94 (ddd, *J* = 8.4, 7.4, 1.6 Hz, 1H), 7.61 (td, *J* = 7.6, 7.1, 1.0 Hz, 1H), 7.53–7.43 (m, 3H), 7.36 (dd, *J* = 7.8, 1.8 Hz, 1H), 2.45 (s, 3H) and 2.34 (s, 3H).

##### (Z)-6-(5-Chloro-3-oxoindolin-2-ylidene)-8-methylindolo[2,1-b]quinazolin-12(6H)-one (**2e**)

In total, 170 mg (0.40 mmol) of **9e** was converted with 17 g of PPA according to the general procedure. The filter paper was extracted in a Soxhlet apparatus with acetone. When the solvent around the thimble turned purple (30 min), the receiving flask was changed and the previously collected extract was discarded. The Soxhlet extraction was continued with acetone and chloroform for 6 h. Removal of the solvents in vacuo yielded a purple solid. Yield: 48 mg (0.12 mmol, 30%), dark purple powder. UV/VIS: λ_max_ (CHCl_3_) = 258 (log(*ε*) 1.66), 351 (1.12), 385 (0.98), 448 (0.74), 545 (1.11) and 588 (1.28) nm. IR (KBr): 3436m(br), 3091w, 2961w, 2913w, 1679s, 1625m, 1602s, 1552m, 1460s, 1346w, 1314m, 1267m, 1197m, 1109m, 1048m, 1020w, 947w, 881w, 820m, 767m, 710w, 684w, 662w and 589w cm^−1^. ^1^H NMR (DMSO-d_6_): δ = 11.71 (s, 1H), 8.89 (s, 1H), 8.34 (d, *J* = 8.1 Hz, 1H), 8.21 (d, *J* = 8.4 Hz, 1H), 8.11 (d, *J* = 8.0 Hz, 1H), 7.91–7.83 (m, 1H), 7.63 (dd, *J* = 8.5, 2.3 Hz, 1H), 7.60 (d, *J* = 2.0 Hz, 1H), 7.57–7.52 (m, 1H), 7.51–7.47 (m, 1H), 7.26 (d, *J* = 8.8 Hz, 1H) and 2.34 (s, 3H). ^13^C NMR (DMSO-d_6_): δ = 186.11, 158.32, 153.55, 149.95, 146.80, 137.62, 136.47, 135.73, 135.34, 134.47, 130.38, 128.10, 127.45, 126.60, 126.08, 125.25, 123.96, 123.92, 120.84, 120.39, 115.62, 115.51, 108.13 and 21.52.

##### (Z)-8-Bromo-6-(5-methyl-3-oxoindolin-2-ylidene)-12-oxo-6,12-dihydroindolo[2,1-b]quinazoline-3-carboxylic acid (**2f**)

In total, 125 mg (0.24 mmol) of **9f** was converted with 11 g of PPA according to the general procedure. Yield: 54 mg (0.11 mmol, 45%), dark violet powder. IR (KBr): 3433s(br), 3113w, 2920w, 1686s, 1609s, 1546s, 1483s, 1429m, 1312s, 1283m, 1258m, 1201m, 1172m, 1119m, 1084m, 1046m, 938w, 878w, 757m, 736w, 710w, 682w, 568w and 507w cm^−1^. ^1^H NMR (DMSO-d_6_): δ = 11.56 (s, 1H), 9.17 (d, *J* = 2.2 Hz, 1H), 8.65 (d, *J* = 1.6 Hz, 1H), 8.35 (d, *J* = 8.6 Hz, 1H), 8.24 (d, *J* = 8.2 Hz, 1H), 7.95 (dd, *J* = 8.2, 1.6 Hz, 1H), 7.56 (dd, *J* = 8.7, 2.2 Hz, 1H), 7.42–7.26 (m, 3H) and 2.27 (s, 3H). HRMS-TOF (*m*/*z*): 498.0154 ([M–H]^−^, calc.: 498.0095, diff.: 11.85 ppm).

##### (Z)-8-Hydroxy-6-(5-hydroxy-3-oxoindolin-2-ylidene)indolo[2,1-b]quinazolin-12(6H)-one (**2g**)

The **9g** (52 mg, 0.13 mmol) was converted with 18 g of PPA according to the general procedure. There was no solid obtained after filtering because everything was dissolved in the aqueous phosphoric acid solution. The product was extracted with Amberlite XAD 1880N for 24 h at RT. The absorption material was filtered, briefly washed with water and the product was eluted with MeOH. After removing the solvent, the crude product was further purified with flash column chromatography (Götec C18 10g, 100:0 water/MeOH to 0:100 water/MeOH). Yield: 1.2 mg (0.003 mmol, 2%). ^1^H NMR (methanol-d_4_): δ = 8.29 (d, *J* = 4.1 Hz, 1H), 7.75 (d, *J* = 8.7 Hz, 1H), 7.57 (d, *J* = 8.2 Hz, 1H), 7.29 (d, *J* = 8.2 Hz, 1H), 7.06 (t, *J* = 7.6 Hz, 1H), 6.82 (d, *J* = 3.7 Hz, 1H), 6.80–6.67 (m, 3H) and 6.47 (d, *J* = 8.7 Hz, 1H). HRMS-TOF (*m*/*z*): 396.0992 ([M–H]^−^, calc. 396.0979, diff. 19.1 ppm).

### 4.2. Used Cell Lines and Cell Cultivation

For our research, we used human B cell precursor leukaemia cells (Nalm-6) (Dr. Karlheinz Seeger, Charité Berlin), human Burkitt-like lymphoma cells (BJAB) (Prof. Dr. Peter T. Daniel, Charité Berlin) and human chronic myeloid leukaemia cells (K-562) (Dr. Karlheinz Seeger, Charité Berlin). In addition, various resistant cell lines were generated in our lab by treating cells with increasing concentrations of cytostatic drugs. This was carried out until the cells tolerated high concentrations without loss of cell viability. BeKa (vincristine-resistant Nalm-6 cells), JeBa (methotrexate-resistant Nalm-6 cells) and JeFri (etoposide-resistant Nalm-6 cells) were made resistant in the manner described above. For BeKa cells, an overexpression of p-glycoprotein was found as a mechanism of resistance [41]. Moreover, daunorubicin-resistant K-562 cells (NiWi-Dau), showing an underexpression of the proapoptotic protein Harakiri, were used. As BiBo cells (vincristine-resistant BJAB cells) show an overexpression of the anti-apoptotic protein Bcl-2, they were used to test the dependence of apoptosis on this protein. The dependence of apoptosis on effector caspase-3 was also determined by using 7CCA cells (doxorubicin-resistant BJAB cells), which underexpress this caspase [42]. The cell lines were incubated in RPMI 1640 (Gibco, Invitrogen, Karlsruhe, Germany) supplemented with 10% (*v*/*v*) heat-inactivated foetal bovine serum (FBS) (Merck KGaA, Darmstadt, Germany) and 1% (*v*/*v*) penicillin streptomycin (ThermoFisher Scientific Inc., Waltham, MA, USA) in 75 cm^2^ cell culture flasks (Corning Inc., Corning, NY, USA) at 37 °C with 5% CO_2_. All cell lines were passaged two times per week and diluted to a concentration of 0.5 × 10^5^ cells/mL. Twenty-four hours prior to an experiment, cells were diluted to a concentration of 3·× 10^5^ cells/mL for creating standardised growth conditions. For the experiments, cells were used in a concentration of 1 × 10^5^ cells/mL. In addition to untreated cells, a solvent control with dimethylsulfoxide (DMSO) (Serva Electrophoresis GmbH, Heidelberg, Germany) was carried out, to which the results of the cells treated with **2g** were referred.

### 4.3. Determination of Cell Concentration

Cell concentration and cell viability were measured using the CASY^®^ Cell Counter and Analyzer System (OMNI Life Science GmbH & Co. KG, Bremen, Germany). Settings were specifically defined for Nalm-6 cells. Cells were treated with **2g** in 6-well plates (Corning Inc., Corning, NY, USA). Cell count was detected after 24 h of incubation. Therefore, 100 µL cell suspension was resuspended in 10 mL CASY^®^ ton (ready to use isotonic saline solution) and measured by the cell counter. The cell count of cells treated with DMSO was defined as the maximum cell count.

### 4.4. Measurement of DNA Fragmentation

DNA fragmentation is a characteristic process in the late phase of apoptosis [15]. The DNA-intercalating dye propidium iodide (PI) was used to make fragmentation measurable by identifying cells with hypodiploid DNA content [43]. Measurements were carried out at the single-cell level using a flow cytometer. The experimental set-up was performed as described above (see Section 4.1). Nalm-6 cells were treated with different concentrations of **2g** in DMSO as solvent for 72 h. After incubation, cells were centrifuged (8000 rpm, 5 min, 4 °C) and fixed in 200 µL of 2% (*v*/*v*) formaldehyde (Carl Roth GmbH & Co. KG, Karlsruhe, Germany) on ice for 30 min. Subsequently, cells were centrifuged again (1500 rpm, 5 min, 4 °C) and incubated with 180 µL of 2:1 (*v*/*v*) ethanol/PBS (Merck KGaA, Darmstadt, Germany, ThermoFisher Scientific Inc., Waltham, USA) for another 15 min on ice. Pelleted by centrifugation, cells were resuspended in 50 µL of RNase A (10 mg/mL) (VWR International GmbH, Darmstadt, Germany) in 1× PBS and incubated for 30 min at 37 °C. The cells were centrifuged (1500 rpm, 5 min, 4 °C) and finally resuspended in 200 µL of 1× PBS containing 50 µg/µL PI. DNA fragmentation was quantified via flow cytometry (FACS Calibur, Becton Dickinson GmbH, Heidelberg, Germany) [44]. The data evaluation was performed by using the Cell Quest Software.

### 4.5. Exclusion of Necrosis via LDH Detection

Necrosis rapidly causes a loss of membrane integrity whereby, i.a., lactate dehydrogenase (LDH) is released, whereas apoptosis does not [45,46]. To exclude necrosis, the Nalm-6 cells were treated for only one hour with different concentrations of **2g** before the LDH assay (Roche Molecular Systems Inc., Rotkreuz, Switzerland) was performed as described in the manual. Cells were incubated in RPMI 1640 without FBS. As a positive control, cells were treated with 2% *v*/*v* Triton X-100 (Merck KGaA, Darmstadt, Germany). The results were defined as 100% cytotoxicity. To exclude possible background signals, the results of the DMSO control were subtracted from all values.

### 4.6. Measurement of the Mitochondrial Membrane Potential

The intrinsic pathway leads to the permeabilisation of the mitochondrial outer membrane (MOMP), which results in a breakdown of the transmembrane potential [23,47]. For quantifying cells with a reduced mitochondrial membrane potential, 5,5′,6,6′-tetrachloro-1,1′,3,3′-tetraethylbenzimidazoylcarbocyanine iodide (JC-1) dye was used and cells were measured by flow cytometry [48]. After treating the cells with different concentrations of **2g** for 48 h, cells were collected by centrifugation (3000 rpm, 5 min, 4 °C) and resuspended in phenol-free RPMI. Then, 6.25 µL of JC-1 (ThermoFisher Scientific Inc., Waltham, USA) was added to each sample, and all were incubated for 30 min (37 °C, 300 rpm). Subsequently, cells were centrifuged (4000 rpm, 4 °C, 5 min) and resuspended in 1× PBS. The percentage of cells with reduced mitochondrial membrane potential were quantified using flow cytometry and Cell Quest Software.

### 4.7. Isolation of Healthy Human Leukocytes

A total of 40 mL of blood was collected and diluted with 14 mL RPMI. An amount of 4 mL of Ficoll (saccharose-epichlorohydrin-Copolymer) was placed in 15 mL of Falcons and 5 mL of diluted blood was carefully added to each. The blood was separated by density gradient centrifugation to obtain the buffy coat containing leucocytes [49,50]. After centrifugation (18 min, 18 °C, 2000 rpm), leukocytes were collected by transferring the buffy coat with a Pasteur pipette into a 50 mL tube (VWR International GmbH, Darmstadt, Germany). Buffy coat was diluted 1:1 with RPMI and centrifuged for another 5 min (18 °C, 2000 rpm). The pellet was resuspended in 10 mL of RPMI. Subsequently, the cell count and viability were determined by using the CASY^®^ Cell Counter and Analyzer System. Cells were seeded with a density of 3×10^5^ cells/mL. The further treatment of the cells was carried out as described above.

### 4.8. Statistics

The results shown correspond to a triplicate determination. The standard deviations (SD) are shown as error bars. If the SD is very small, error bars may not be seen. Significance was calculated after normalization to DMSO using a two-tailed *t*-test with a significance level of 0.05. Graphs and statistics were created using Microsoft Office Excel.

## 5. Conclusions

All in all, dihydroxyquingdainone (**2g**) is a very interesting compound among all of the tested quingdainones, especially in terms of overcoming a wide variety of cytostatic resistances. Inhibition of proliferation and specific apoptosis induction in selected malignant cell lines (human B cell precursor leukaemia cells, human Burkitt-like lymphoma cells and human chronic myeloid leukaemia cells) could be shown. Furthermore, as a first indication of the mechanism of action, involvement of the intrinsic signalling pathway has already been proven. Dihydroxyquingdainone **2g** induced significantly less apoptosis in healthy human leukocytes than in malignant cell lines. In particular, the overcoming of resistances in acute lymphoblastic leukaemia cells is of great importance, as this represents a major advantage for the treatment of cytostatic-resistant tumour cells. The data are promising for further investigation of dihydroxyquingdainone **2g** for the treatment of malignancies, but also for the exploration of other quingdainones, as a strong structure-dependent anti-tumour effect was shown.

## Figures and Tables

**Table 1:** Synthesised quingdainone variants with their respective substituents.

Substance	R	R’	R’’
**2a**	H	H	5’’ -CH_3_
**2b**	5-Cl	H	5’’ -CH_3_
**2c**	5-CH_3_	H	5’’ -CH_3_
**2d**	5-Br	H	5’’ -CH_3_
**2e**	5-CH_3_	H	5’’ -Cl
**2f**	5-Br	3’-COOH	5’’ -CH_3_
**2g**	5-OH	H	5’’ -OH

**Table 2:** AC_50_ values of the synthesised quingdainone variants. They describe the concentration of compounds able to induce apoptosis in 50 percent of the Nalm-6 cells after 72 h of incubation. Compounds with no significant apoptosis induction up to 50 µM are marked with a slash. Quingdainone is not active at all in our cell lines.

Substance	AC_50_
**2a**	>50 µM
**2b**	>50 µM
**2c**	/
**2d**	/
**2e**	/
**2f**	/
**2g** **Quingdainone (R=R’=R’’= H)**	<7.5 µM /

## Data Availability

Not applicable.

## Supplementary Materials

The following supporting information can be downloaded at: https://www.mdpi.com/article/10.3390/molecules27155038/s1, Figure S1: Reduction of membrane potential with 2g/Vcr compared:JC-1 after 48 h. Figure S2: Original NMR, IR, Massspectrometry of compounds.

## Sample Availability

Samples of the compounds are available from the authors.

## Abbreviations

VCR, VINC—vincristine, MTX—methotrexate, Eto.—etoposide, Doxo—doxorubicine, PPA—polyphosphoric acid, AC_50_—the concentration of compounds able to induce apoptosis in 50 percent of cells after 72 h of incubation, LDH—lactate dehydrogenase.
