# Supplementary figures and images for: Dihydroxyquingdainone Induces Apoptosis in Leukaemia and Lymphoma Cells via the Mitochondrial Pathway in a Bcl-2- and Caspase-3-Dependent Manner and Overcomes Resistance to Cytostatic Drugs In Vitro
Source: Molecules. 2022 Aug 8;27(15):5038. doi: 10.3390/molecules27155038 (PMC9370279; doi:10.3390/molecules27155038)

4b

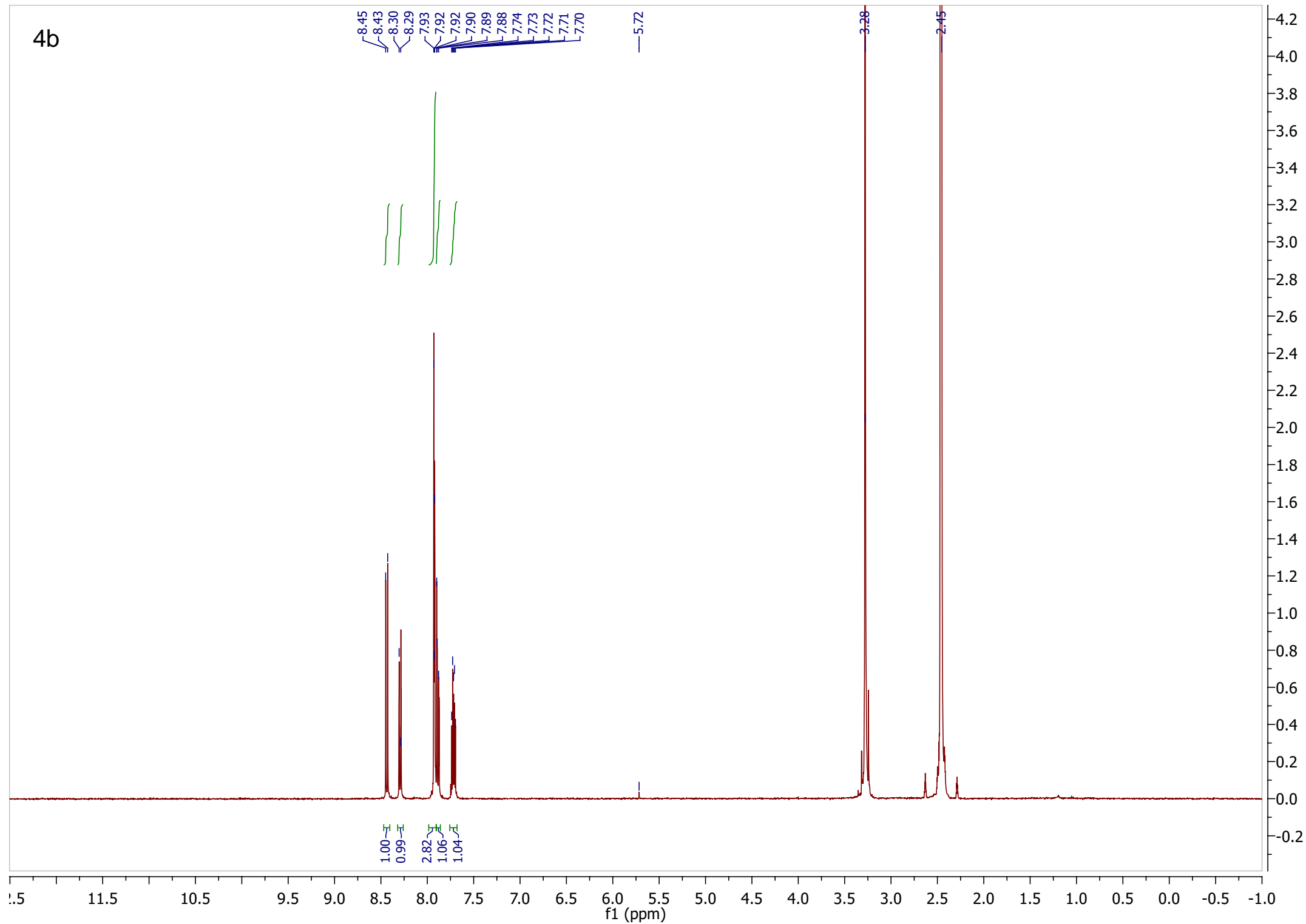

4b

181.86  
158.17  
146.92  
145.50  
144.97  
137.36  
135.88  
131.74  
130.58  
130.53  
127.53  
124.83  
124.54  
123.66  
119.15

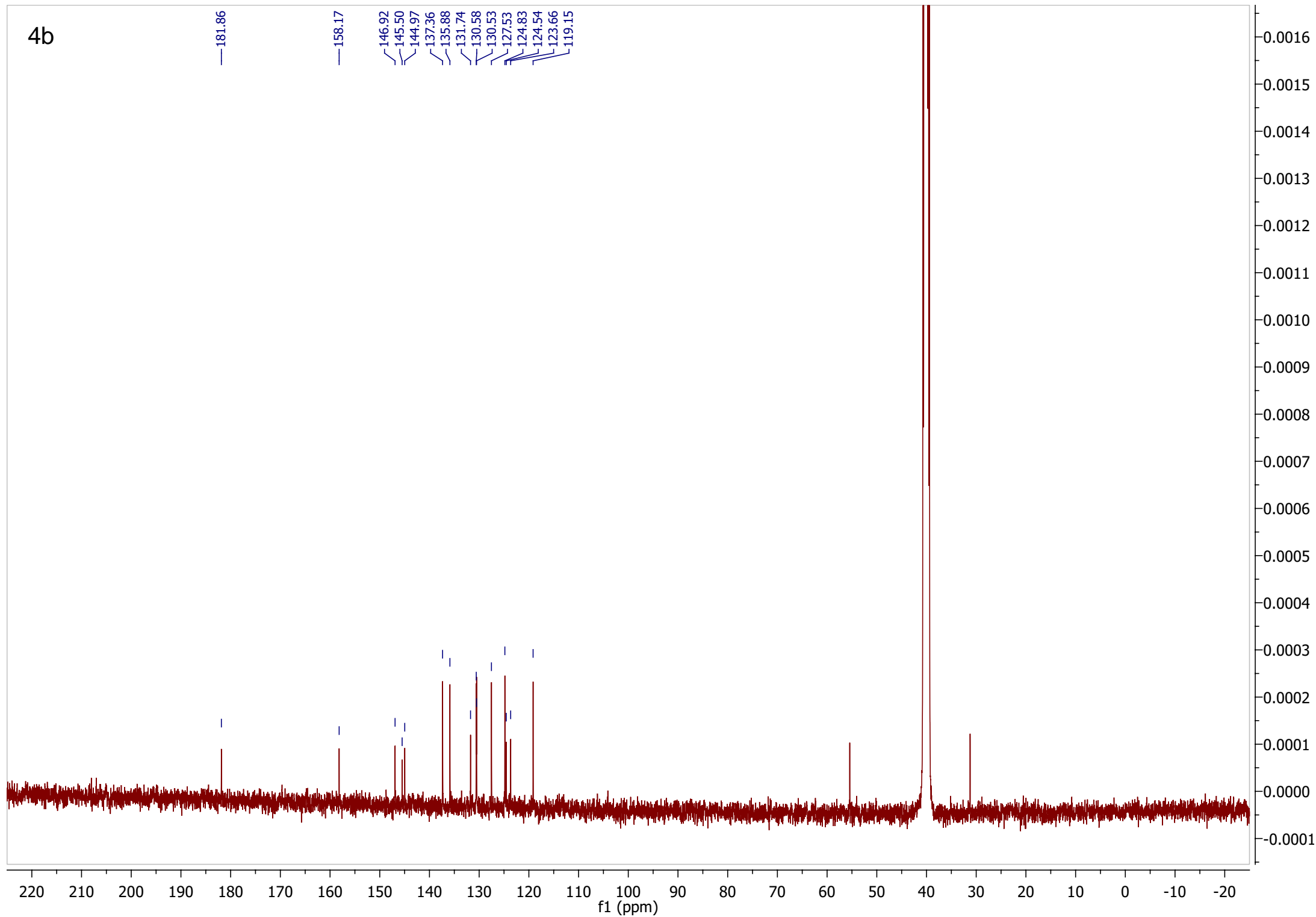

4c

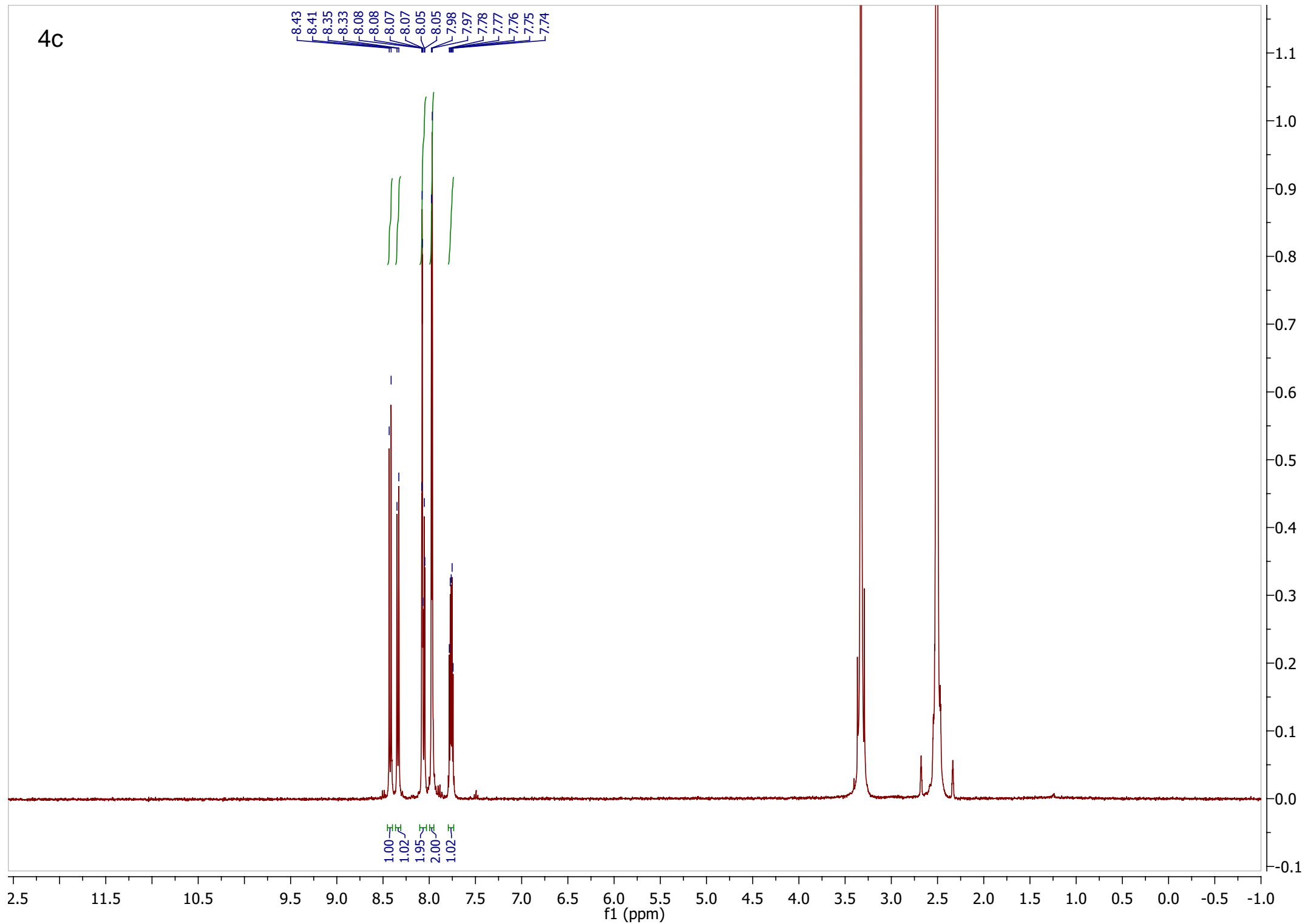

4c

—181.74  
—158.17  
146.93  
145.33  
140.23  
135.89  
130.58  
130.52  
127.64  
127.53  
124.80  
123.66  
119.66  
119.47

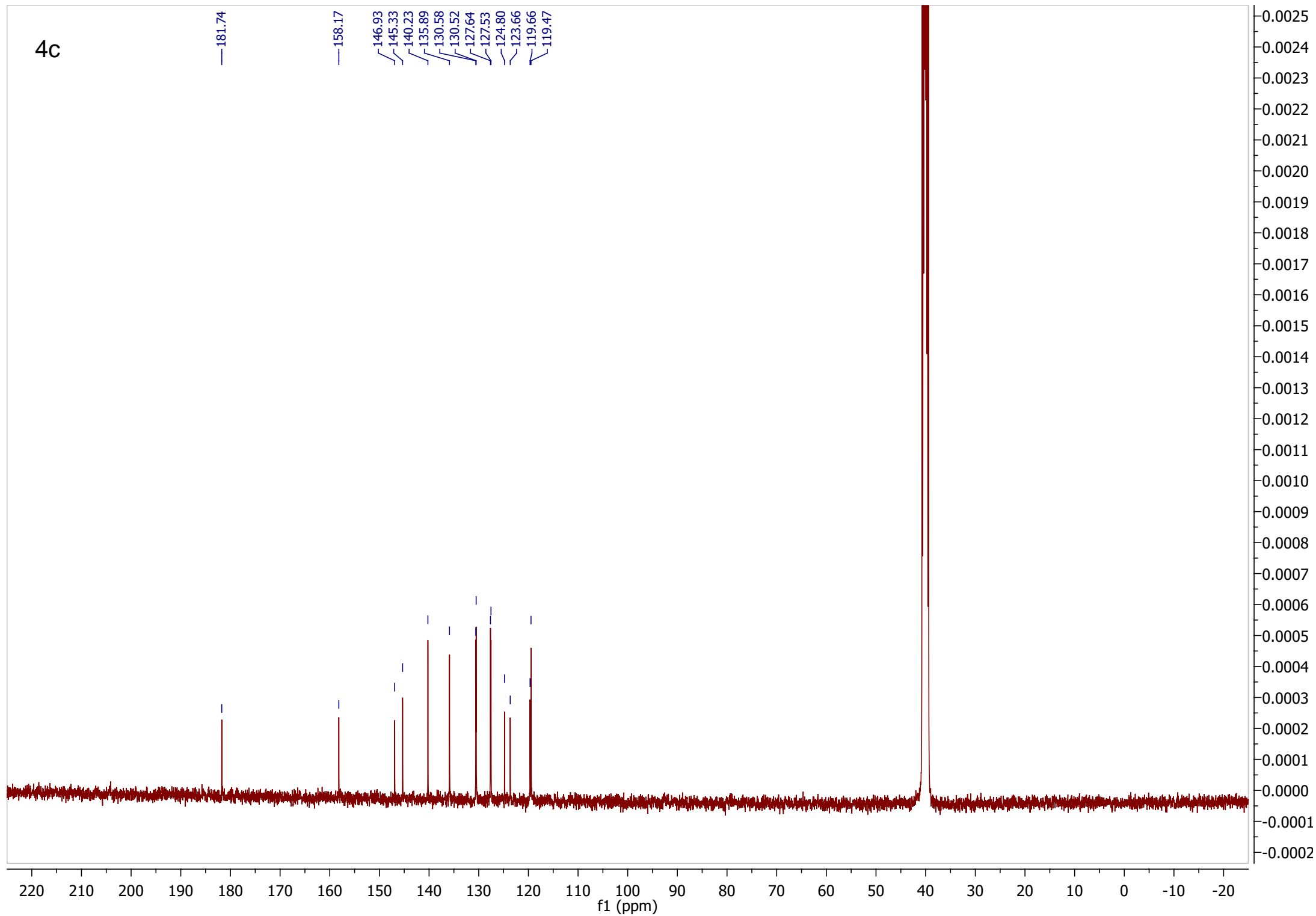

4d

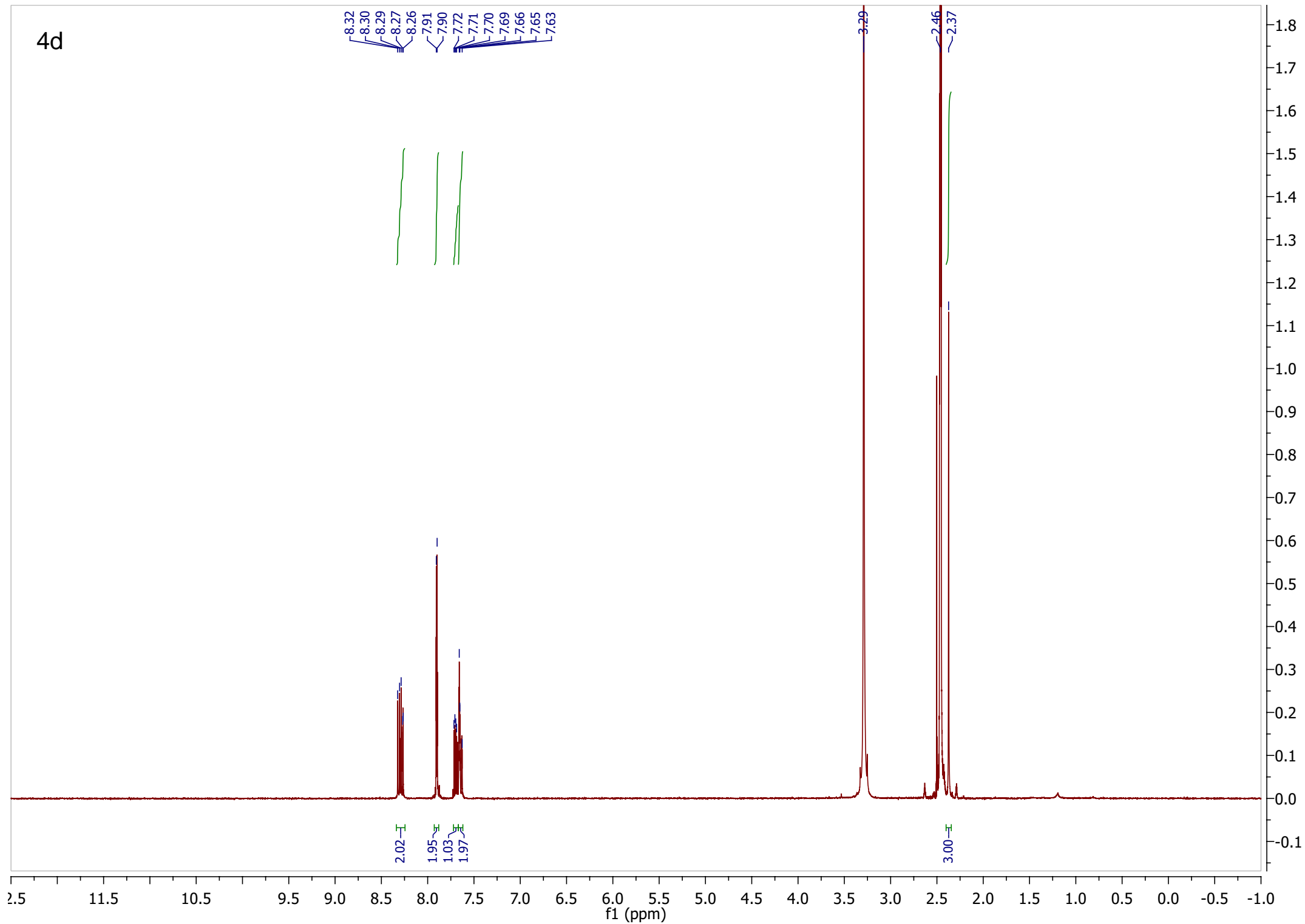

4d

—183.06  
—158.06  
147.02  
145.76  
—144.51  
138.65  
137.20  
135.59  
130.42  
130.33  
127.41  
125.30  
123.89  
122.84  
117.33

—20.96

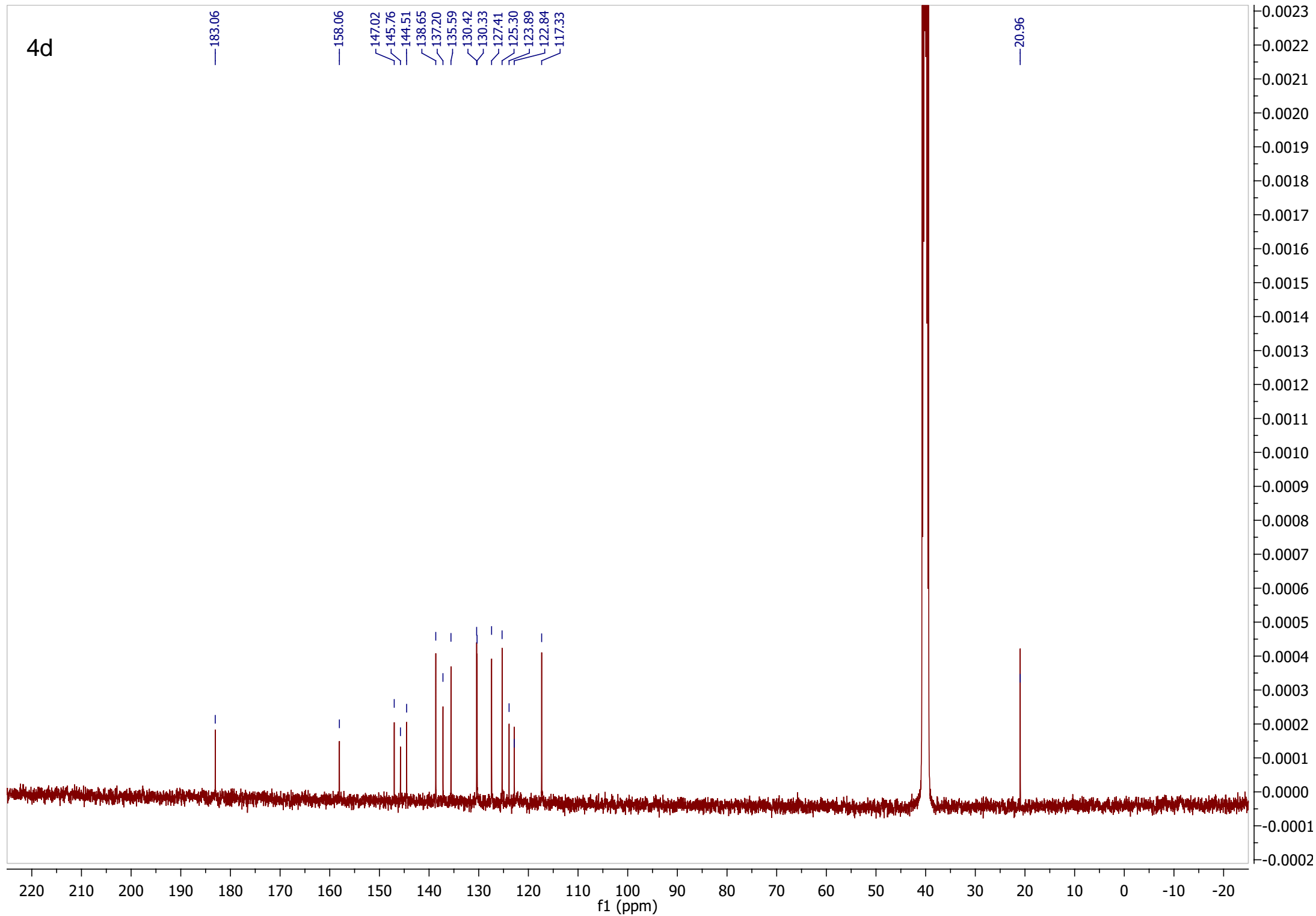

4e

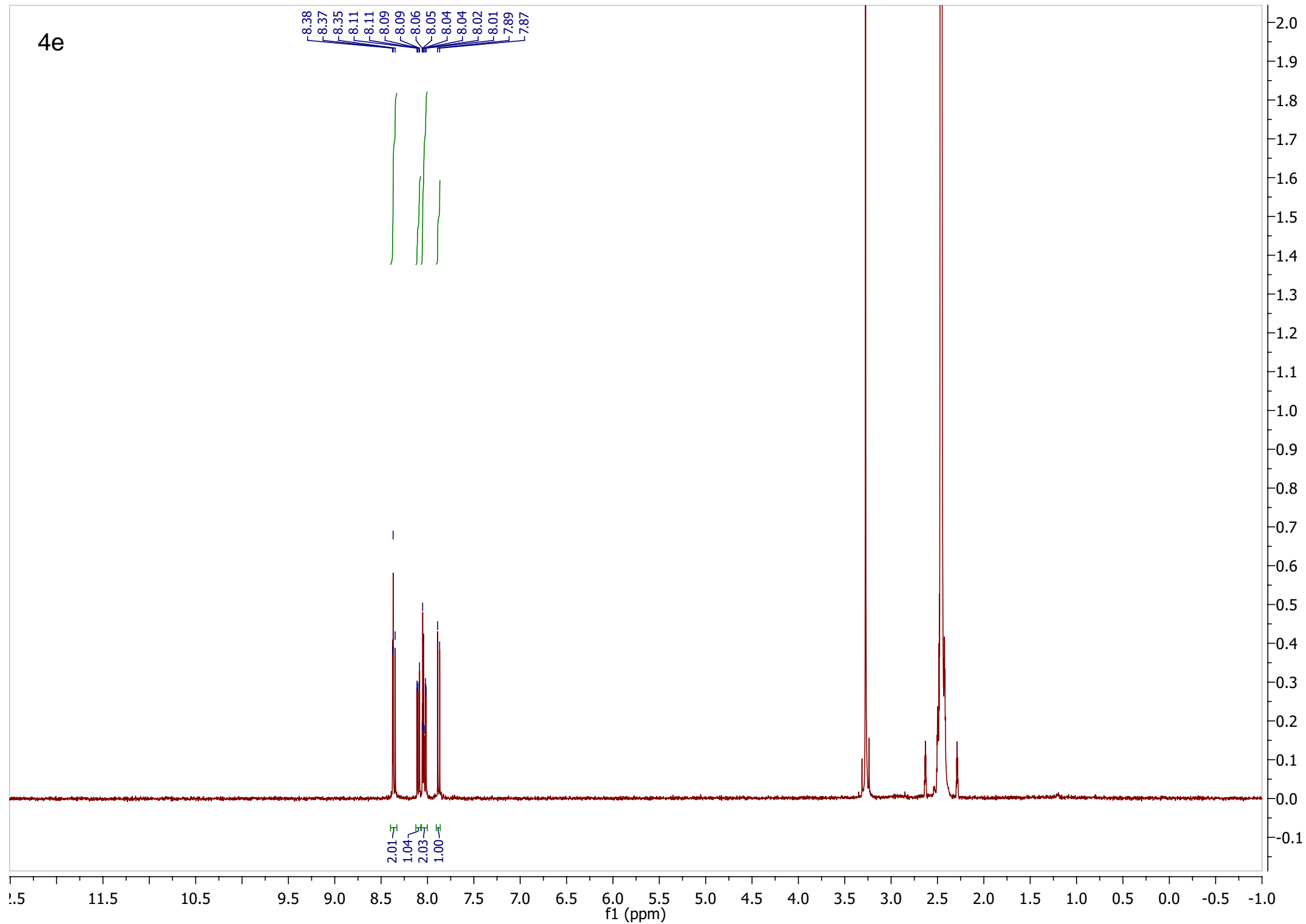

4e

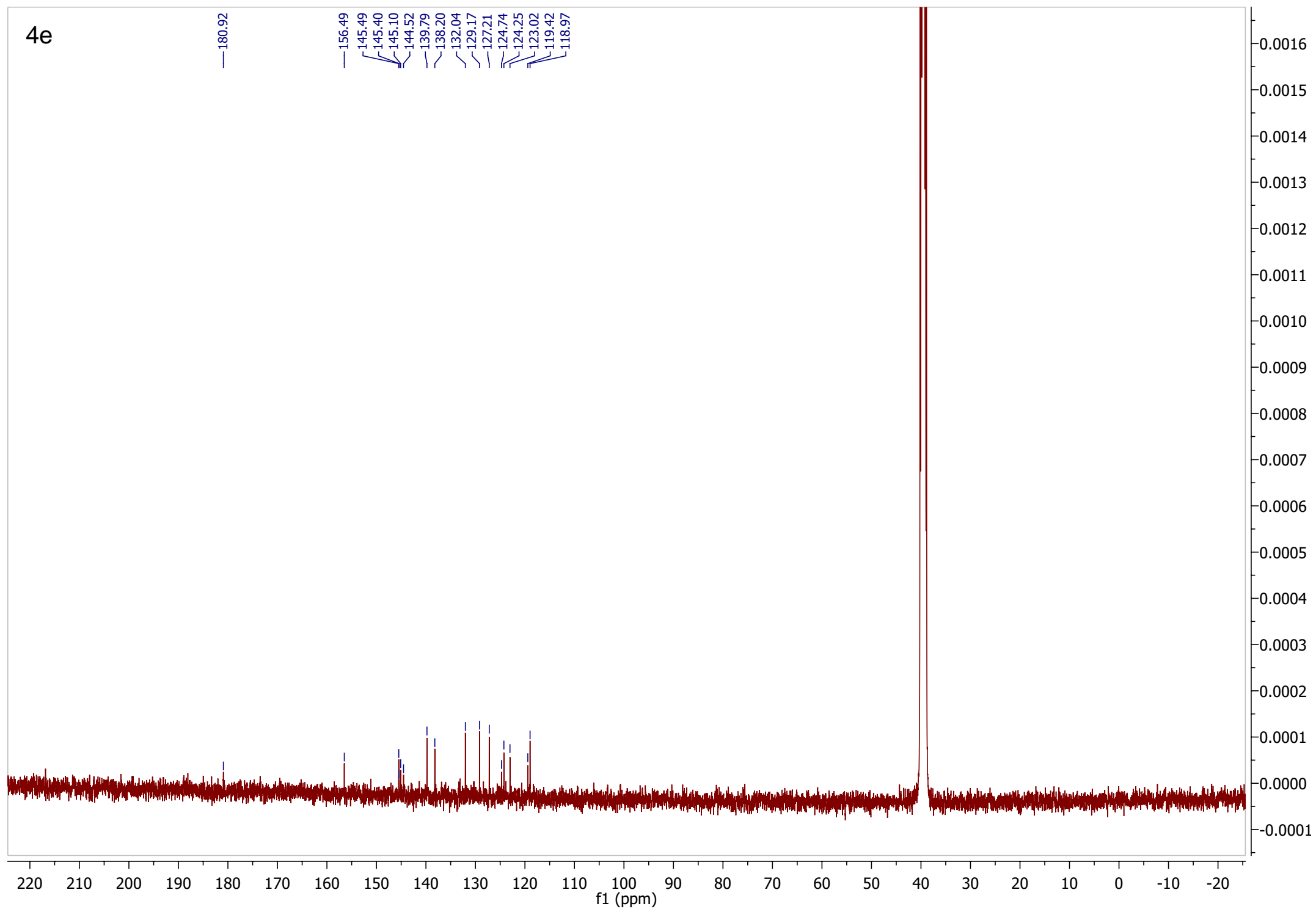

4f

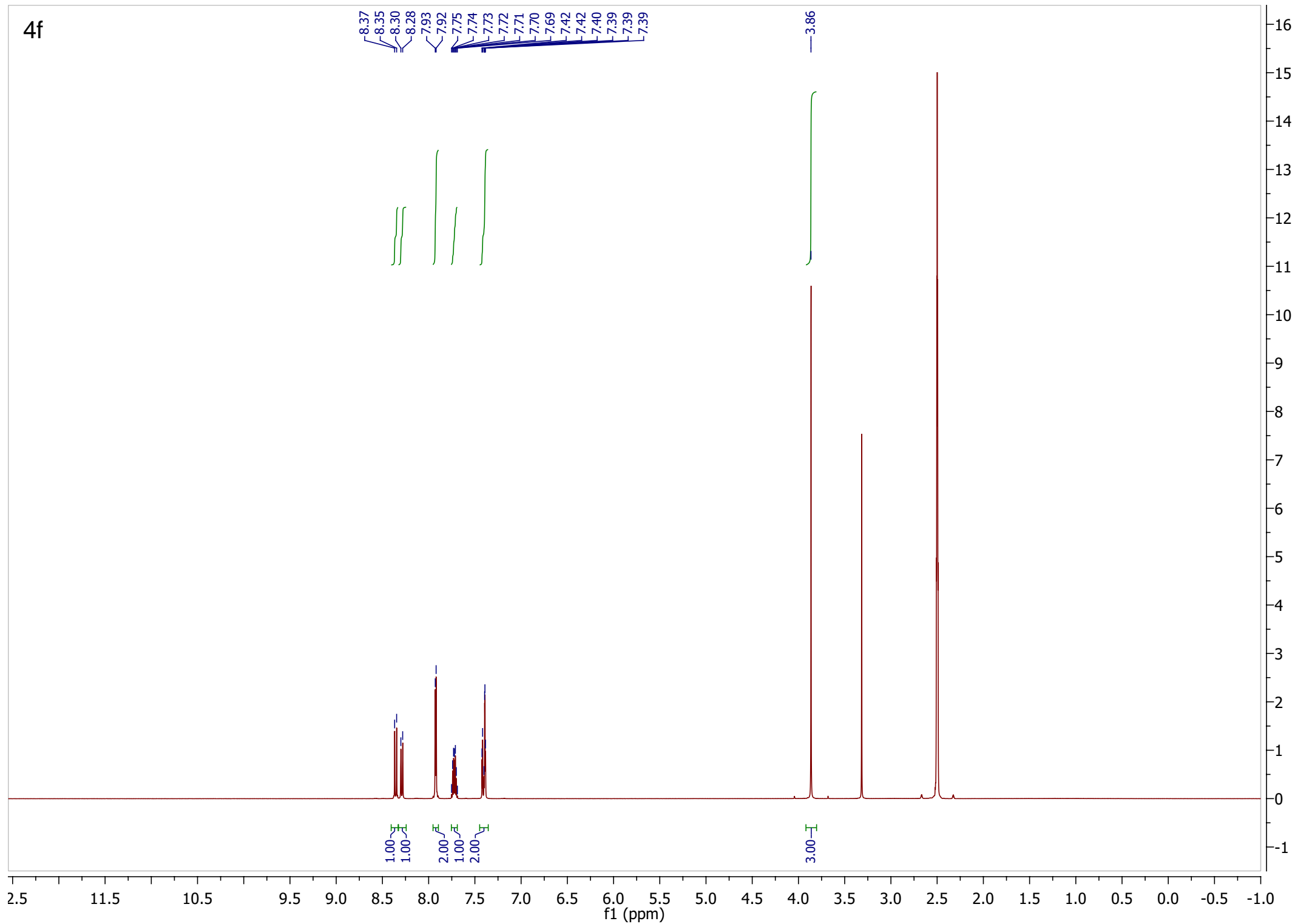

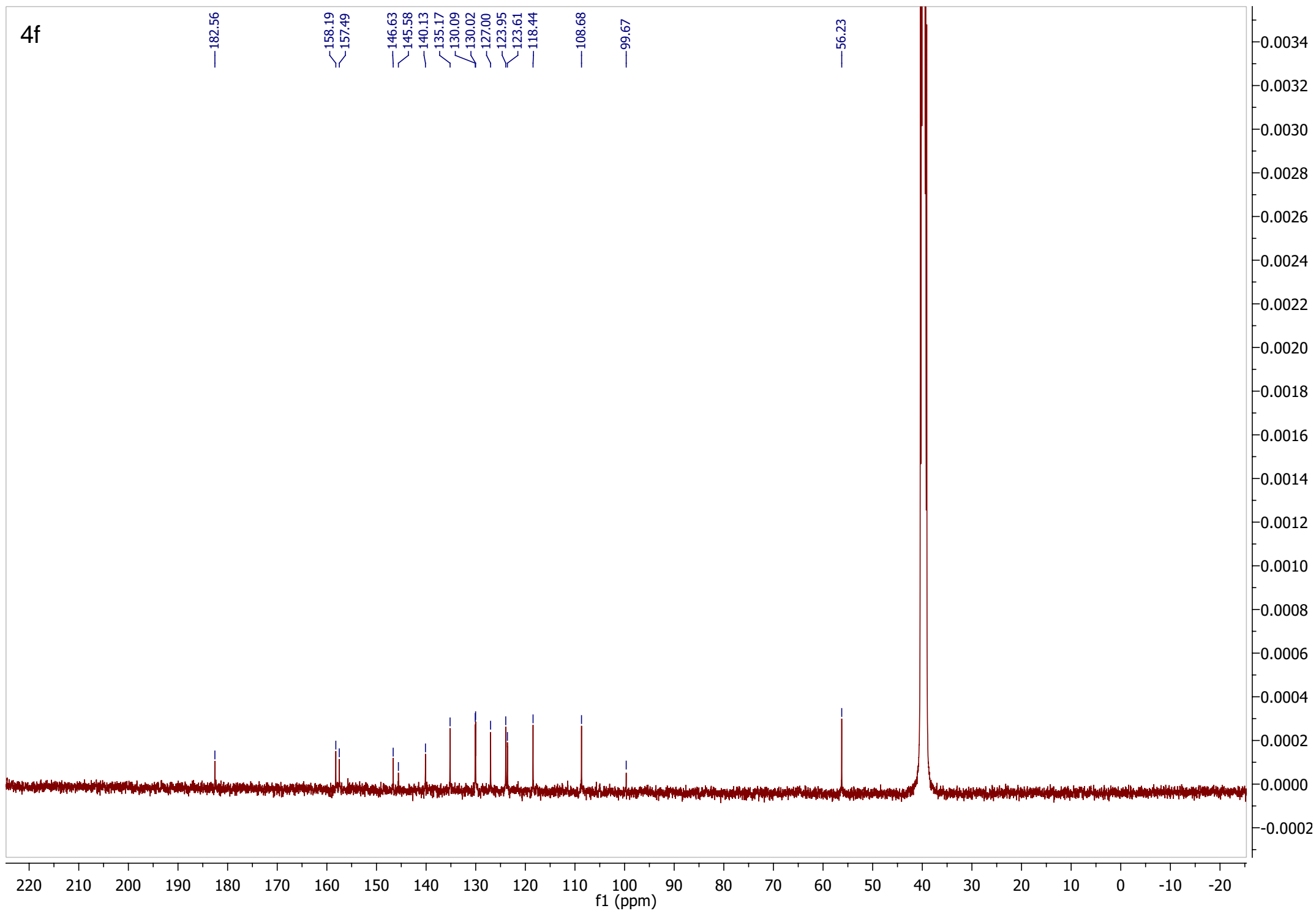

7a

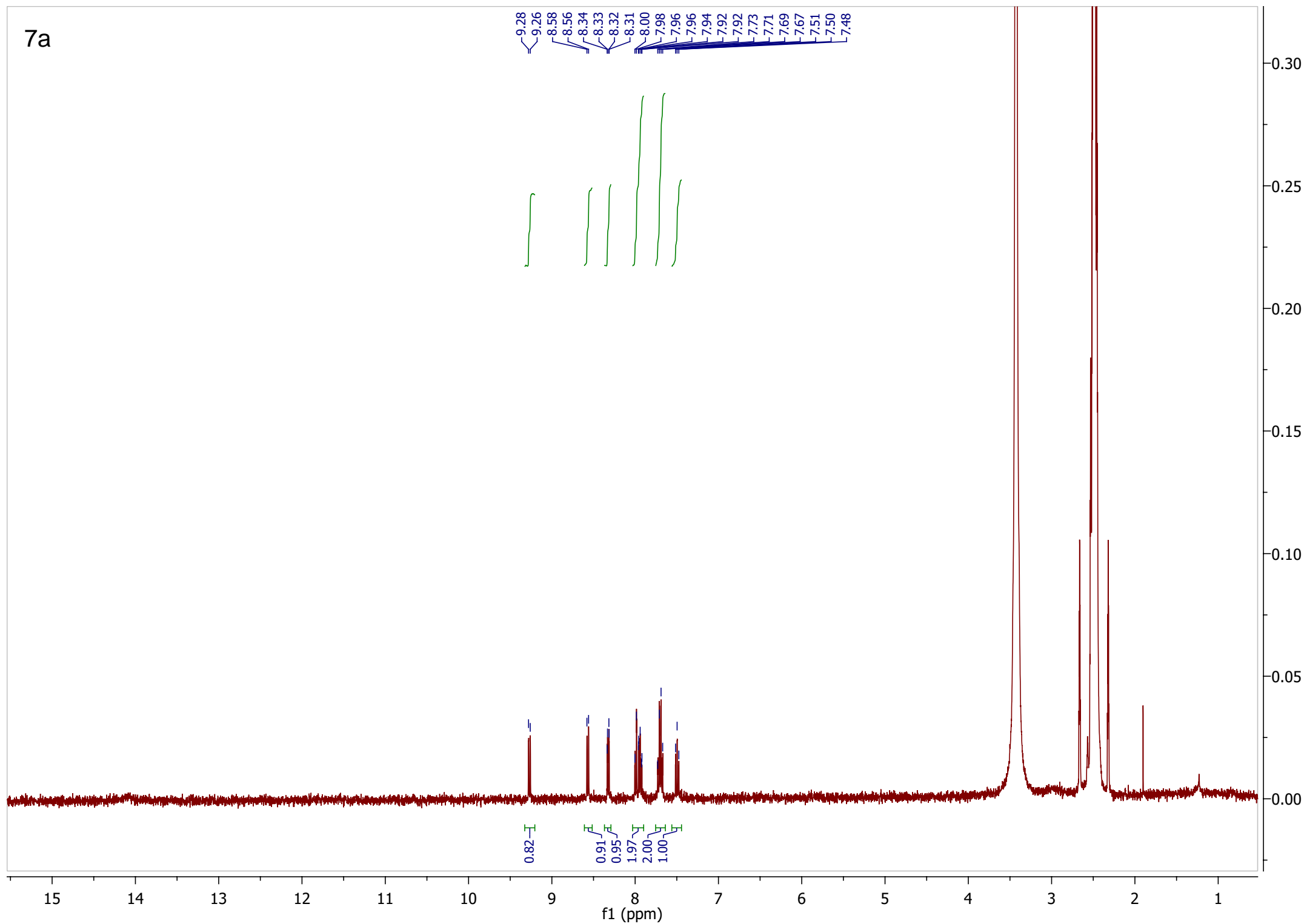

7b

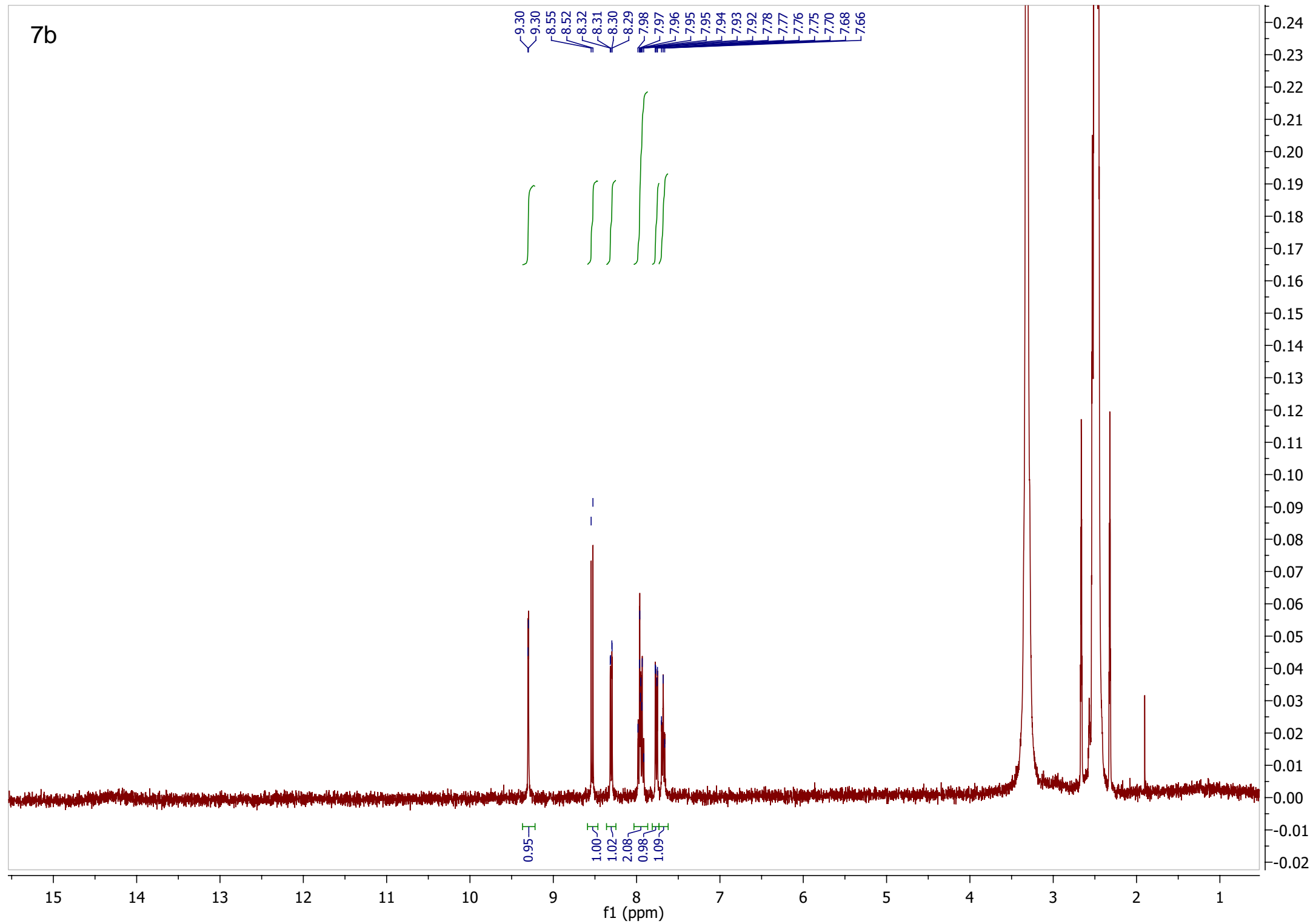

7c

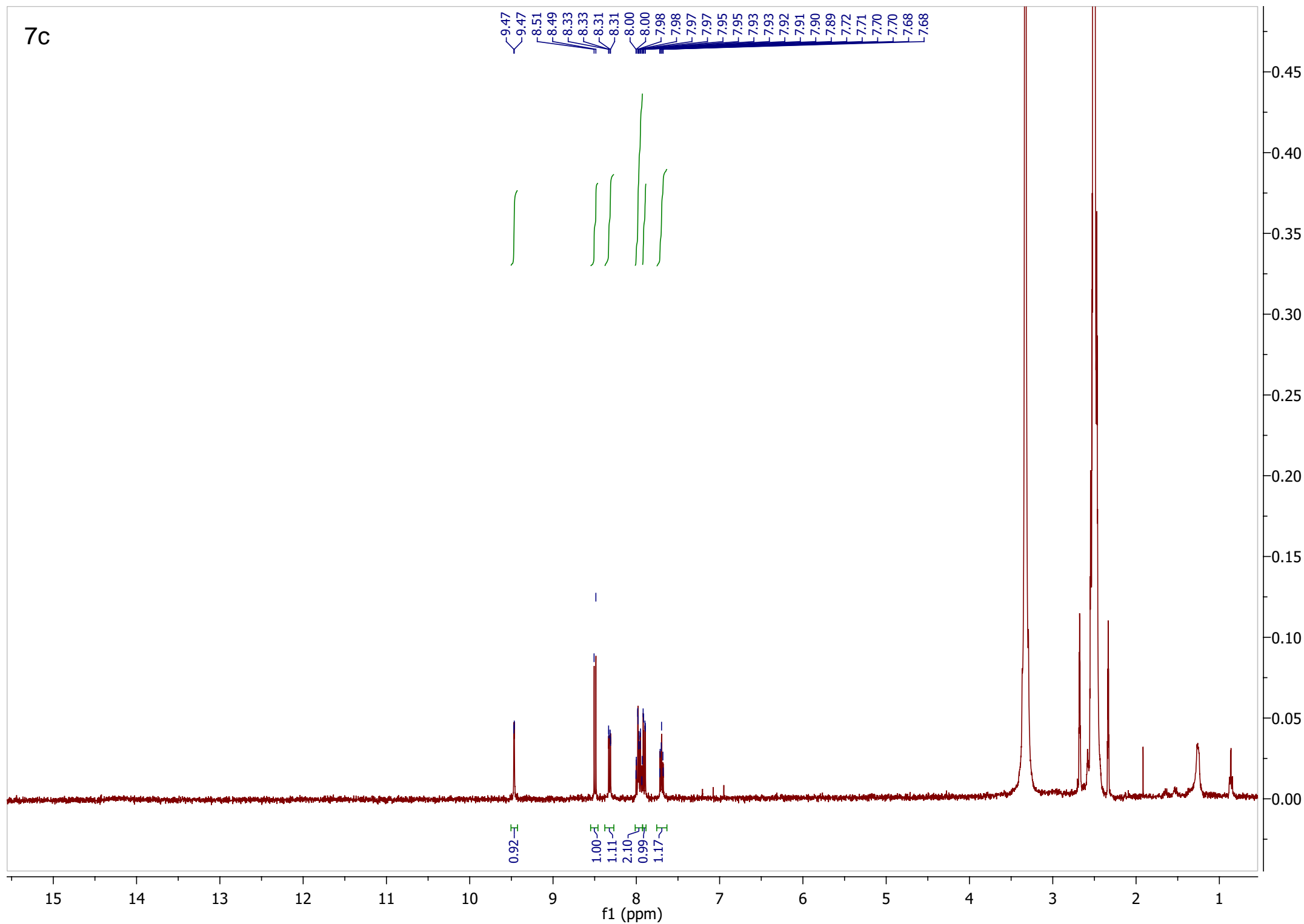

7d

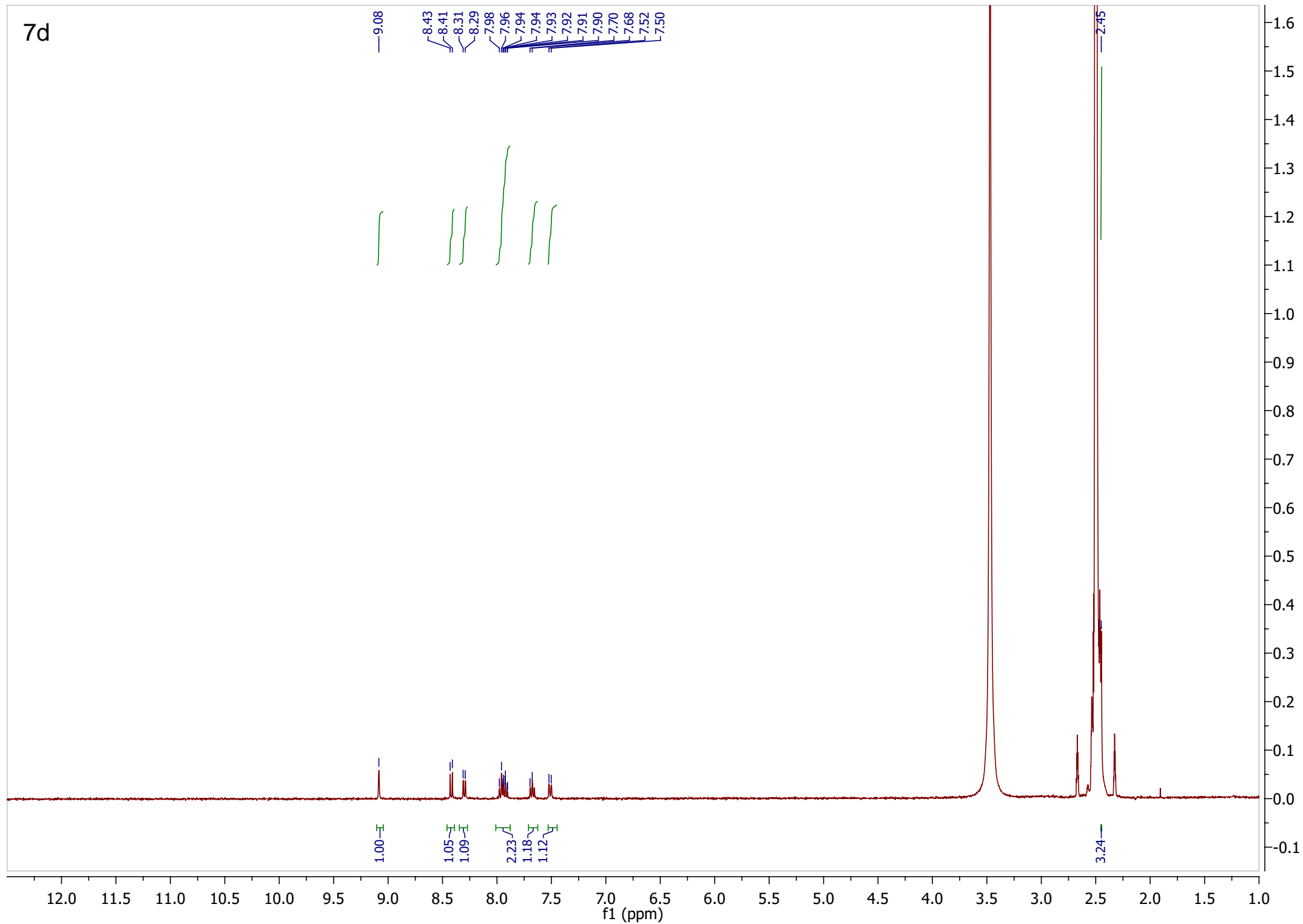

7e

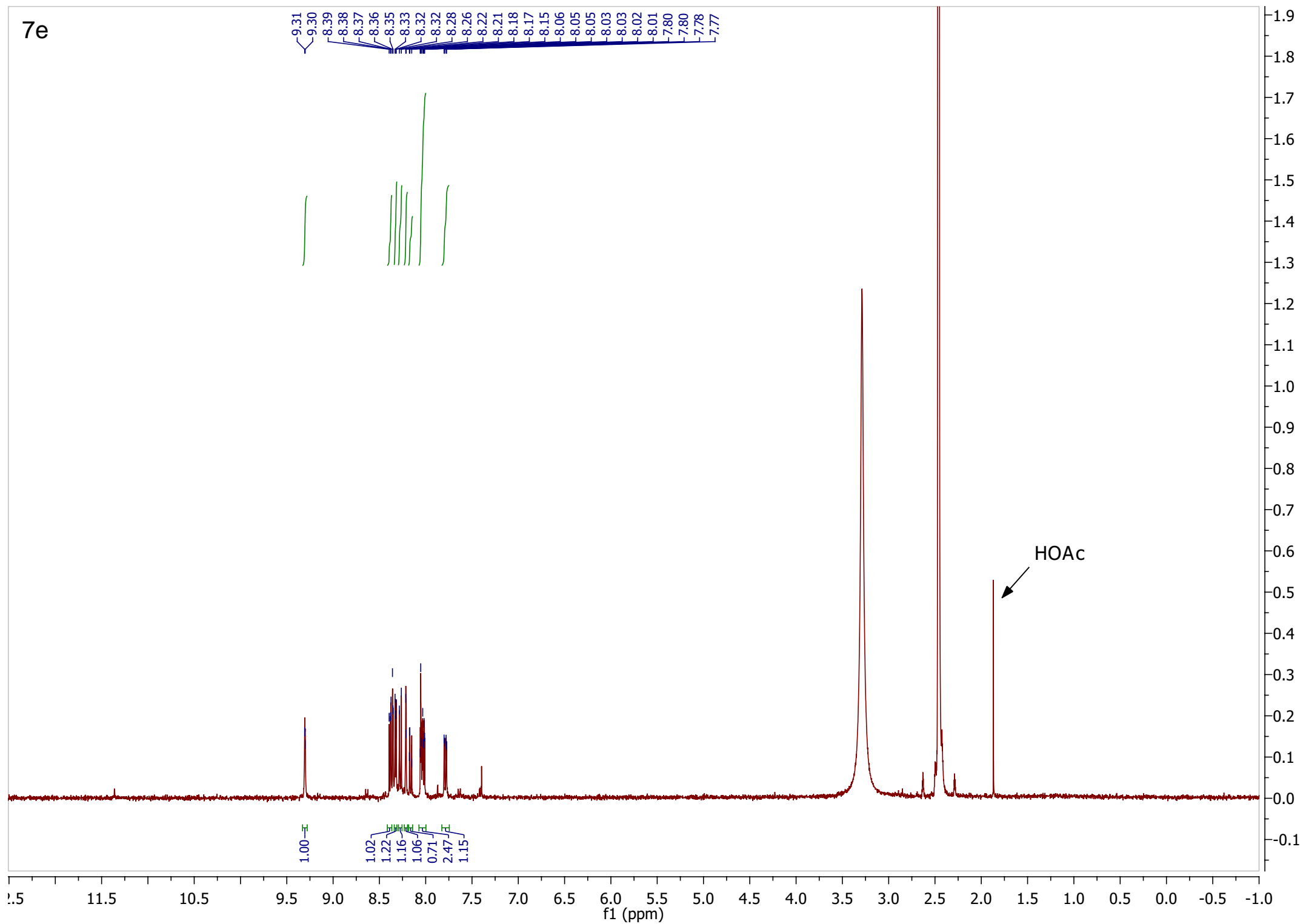

7f

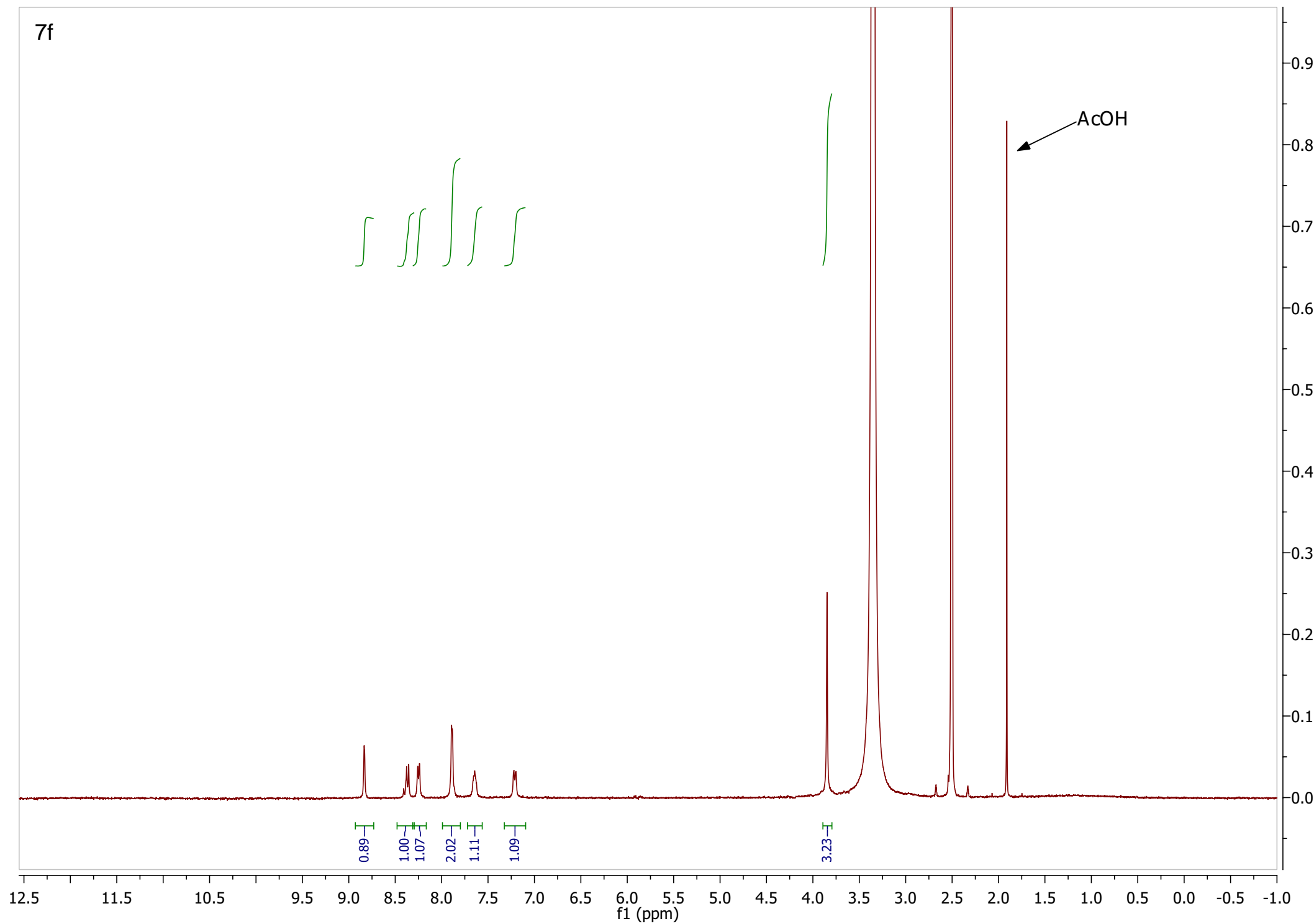

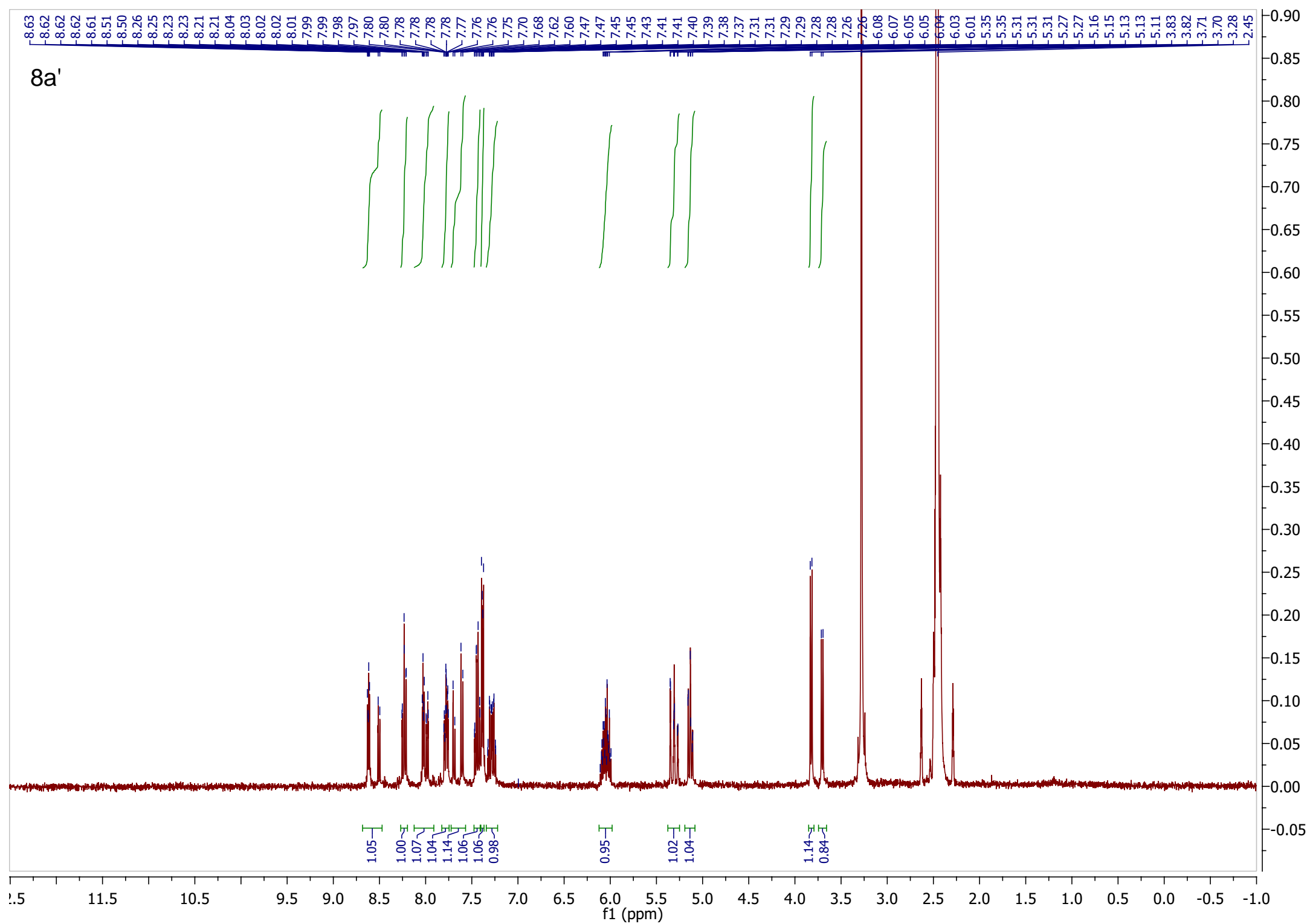

8b

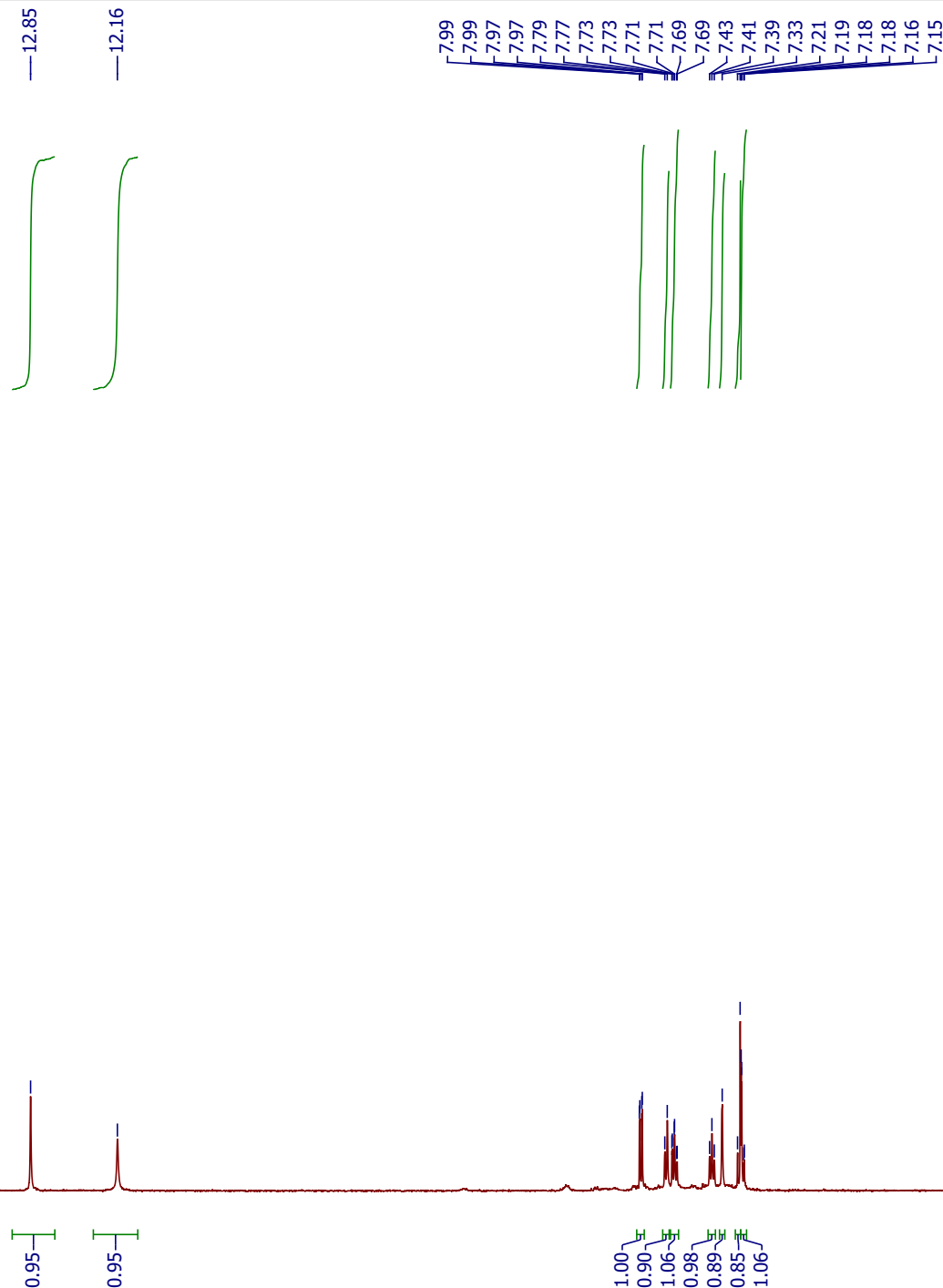

f1 (ppm)

8b

—193.11

—169.88

—167.18

—154.29

136.97

134.43

133.41

131.96

127.34

126.70

125.05

124.76

124.57

124.14

118.37

113.54

111.02

40.72

40.67

40.51

40.46

40.35

40.04

39.83

39.62

39.41

0.0038

0.0036

0.0034

0.0032

0.0030

0.0028

0.0026

0.0024

0.0022

0.0020

0.0018

0.0016

0.0014

0.0012

0.0010

0.0008

0.0006

0.0004

0.0002

0.0000

-0.0002

f1 (ppm)

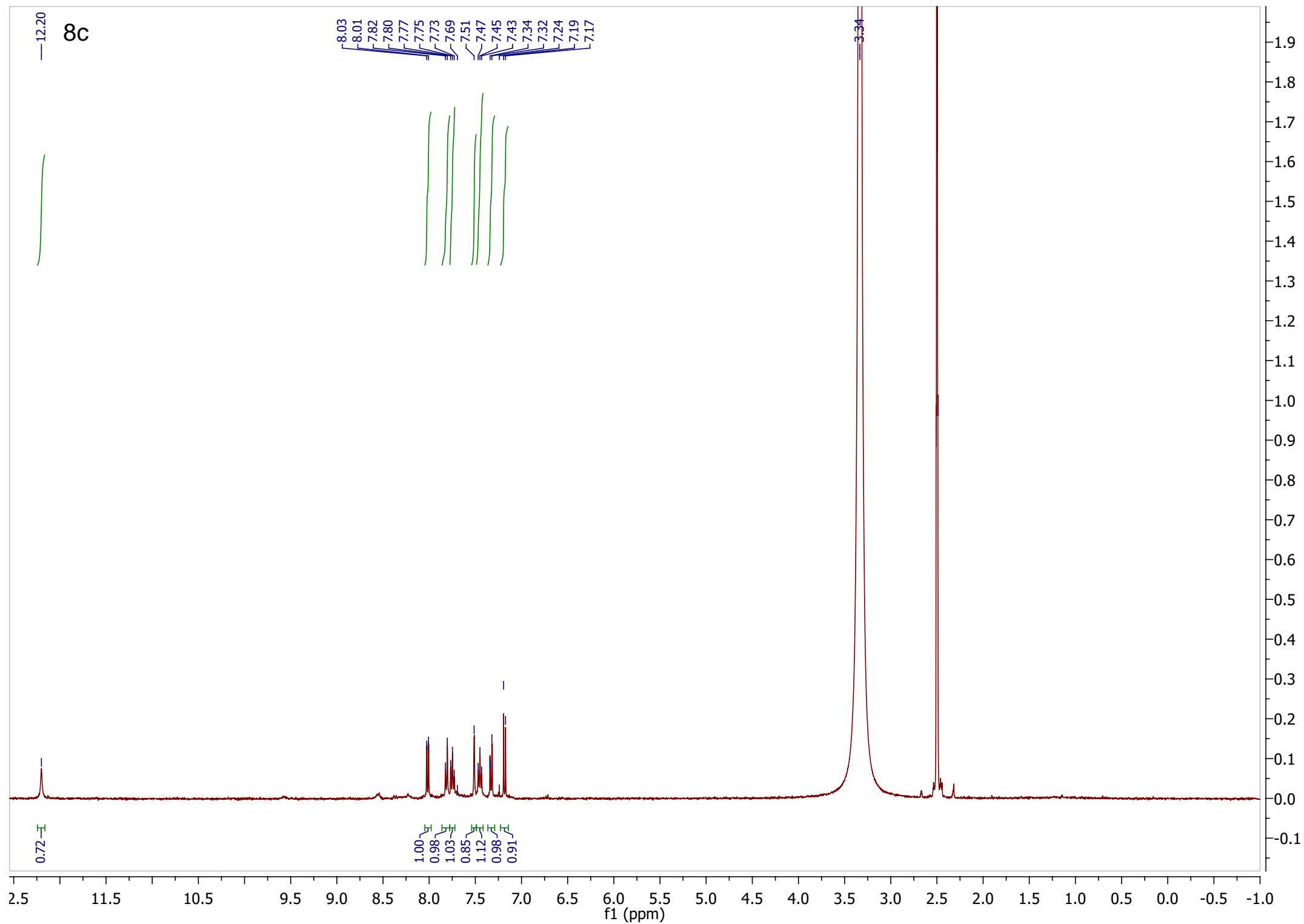

8c

—193.11

—169.89

—167.18

—154.12

136.95

134.43

133.73

131.96

126.88

126.71

125.06

124.58

121.22

118.04

115.32

114.00

110.89

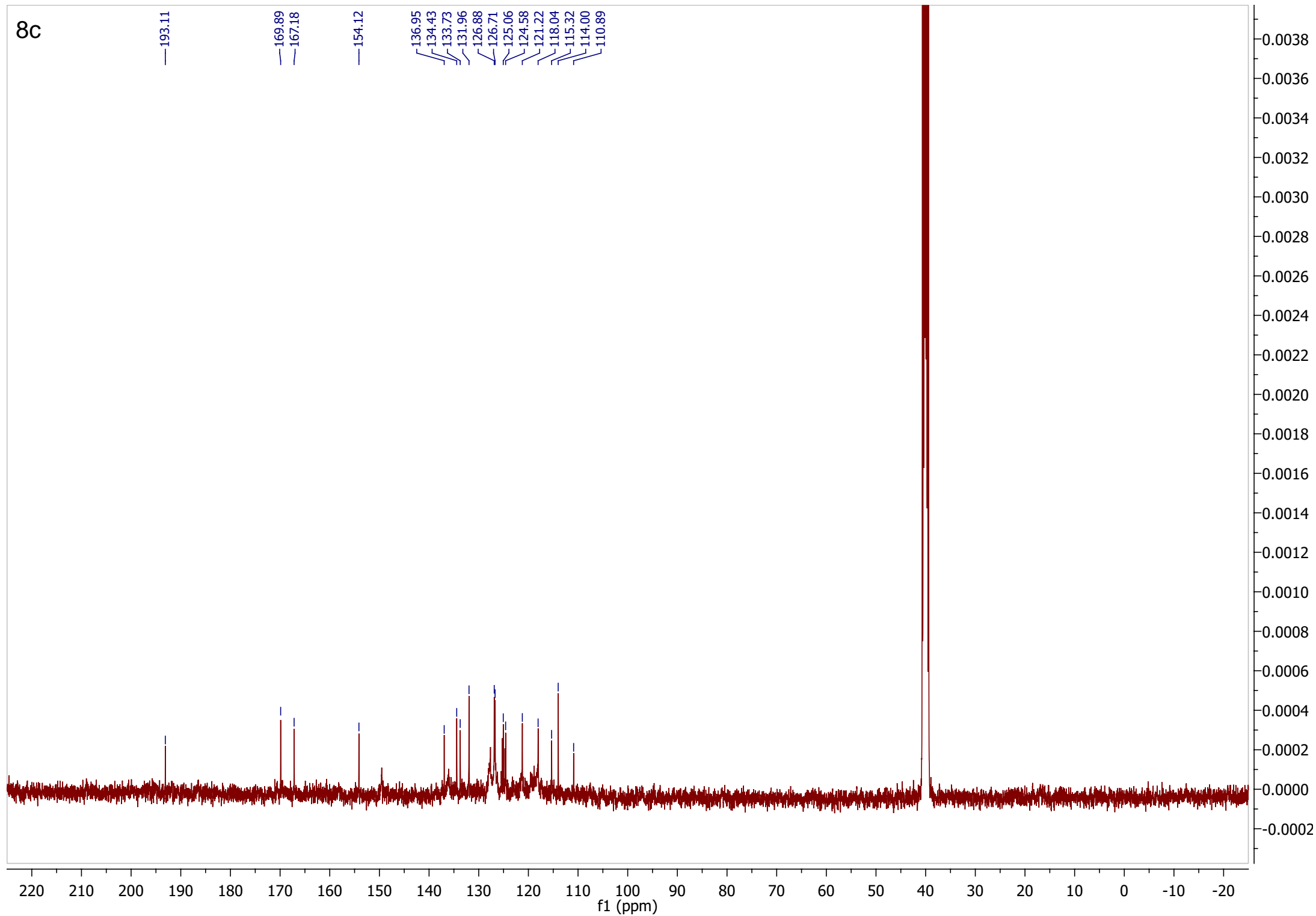

8d'

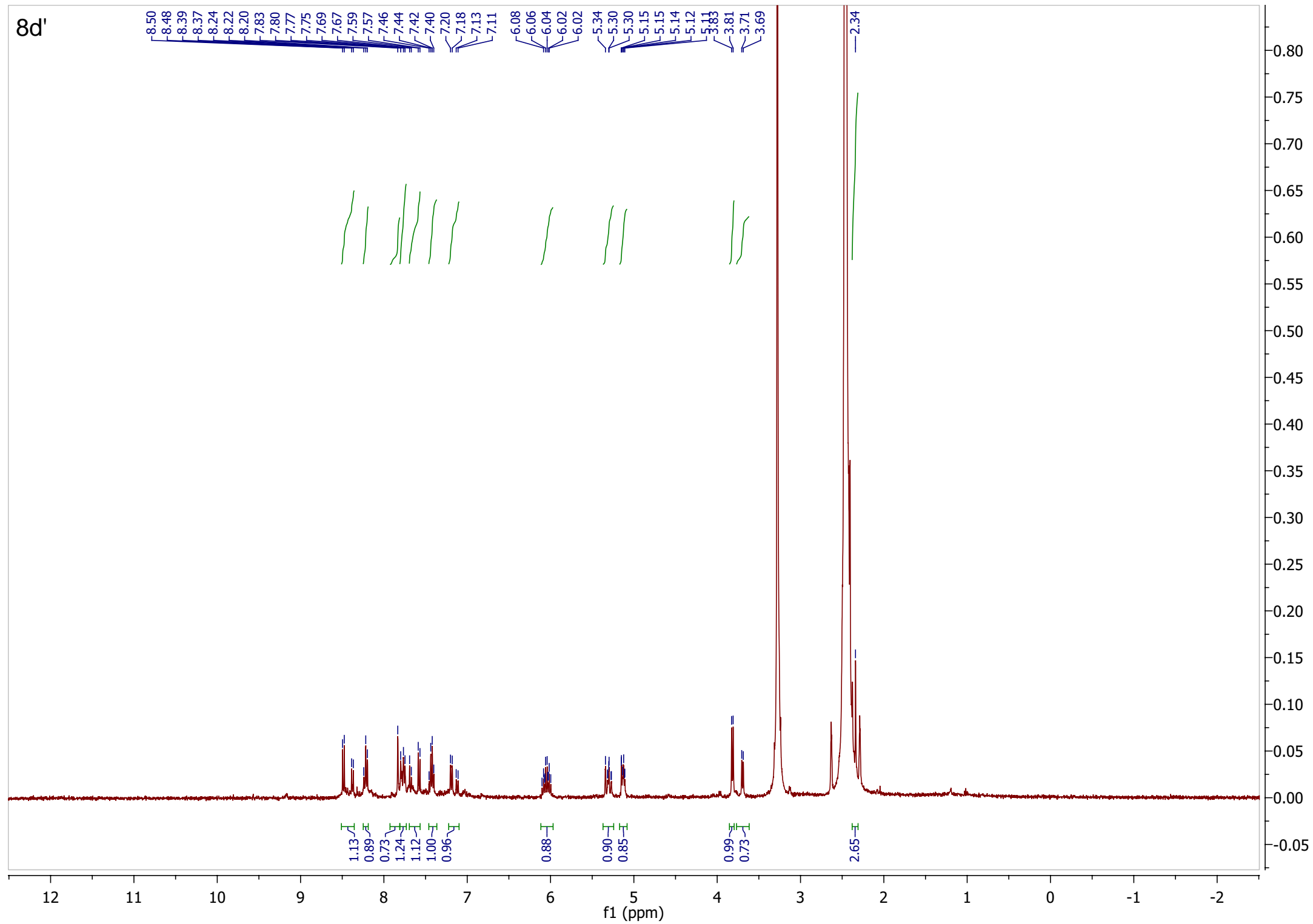

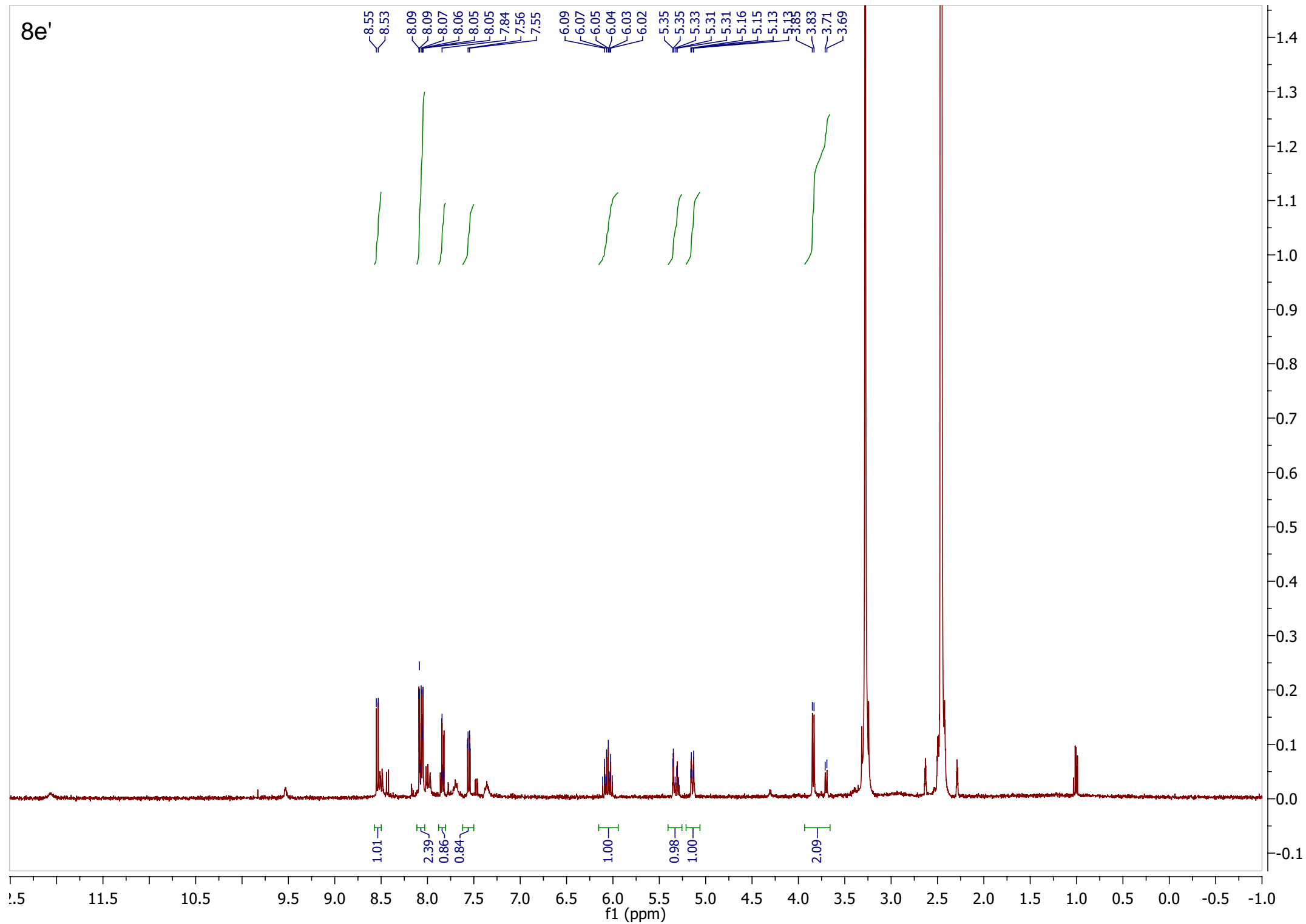

9b

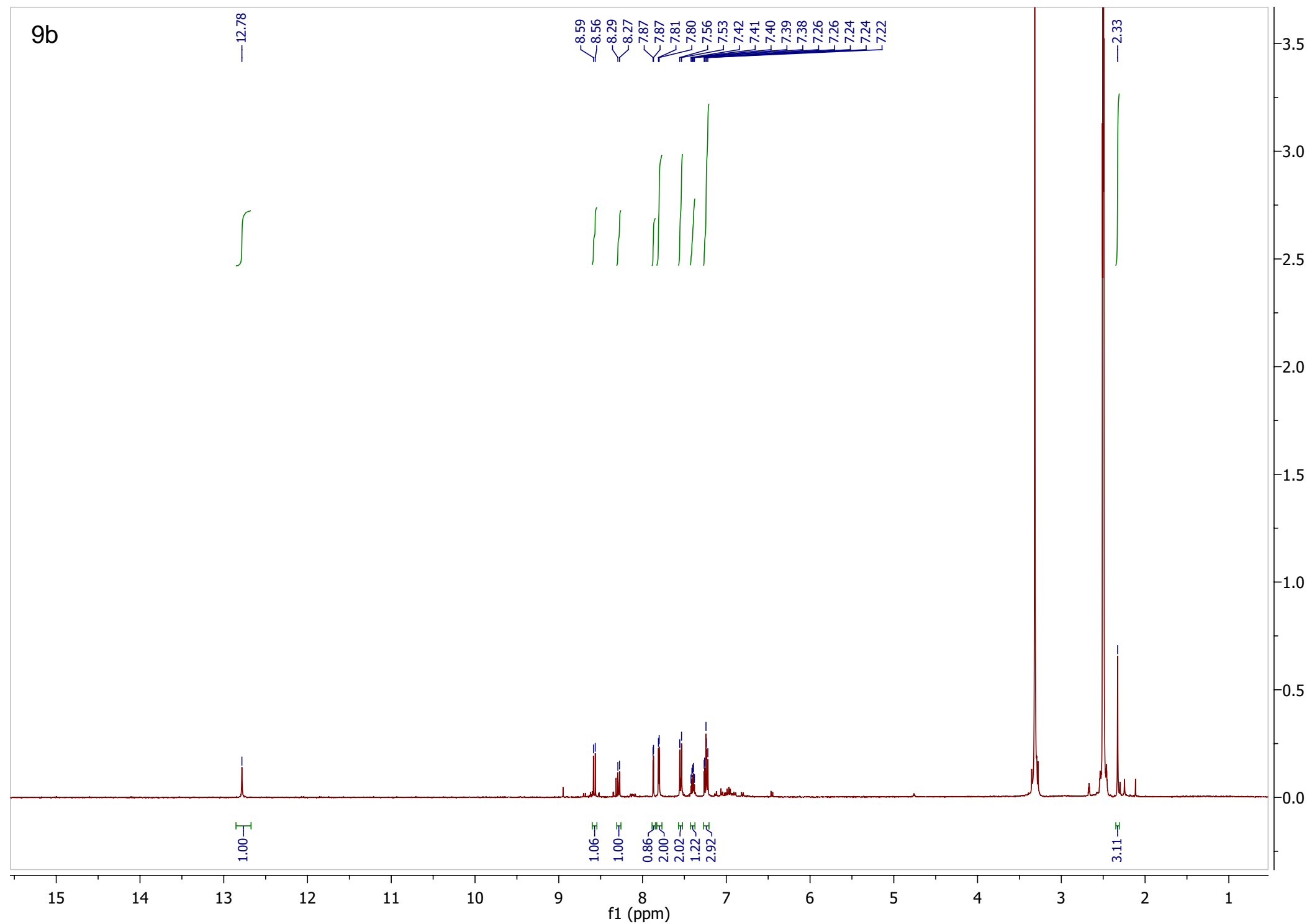

9c

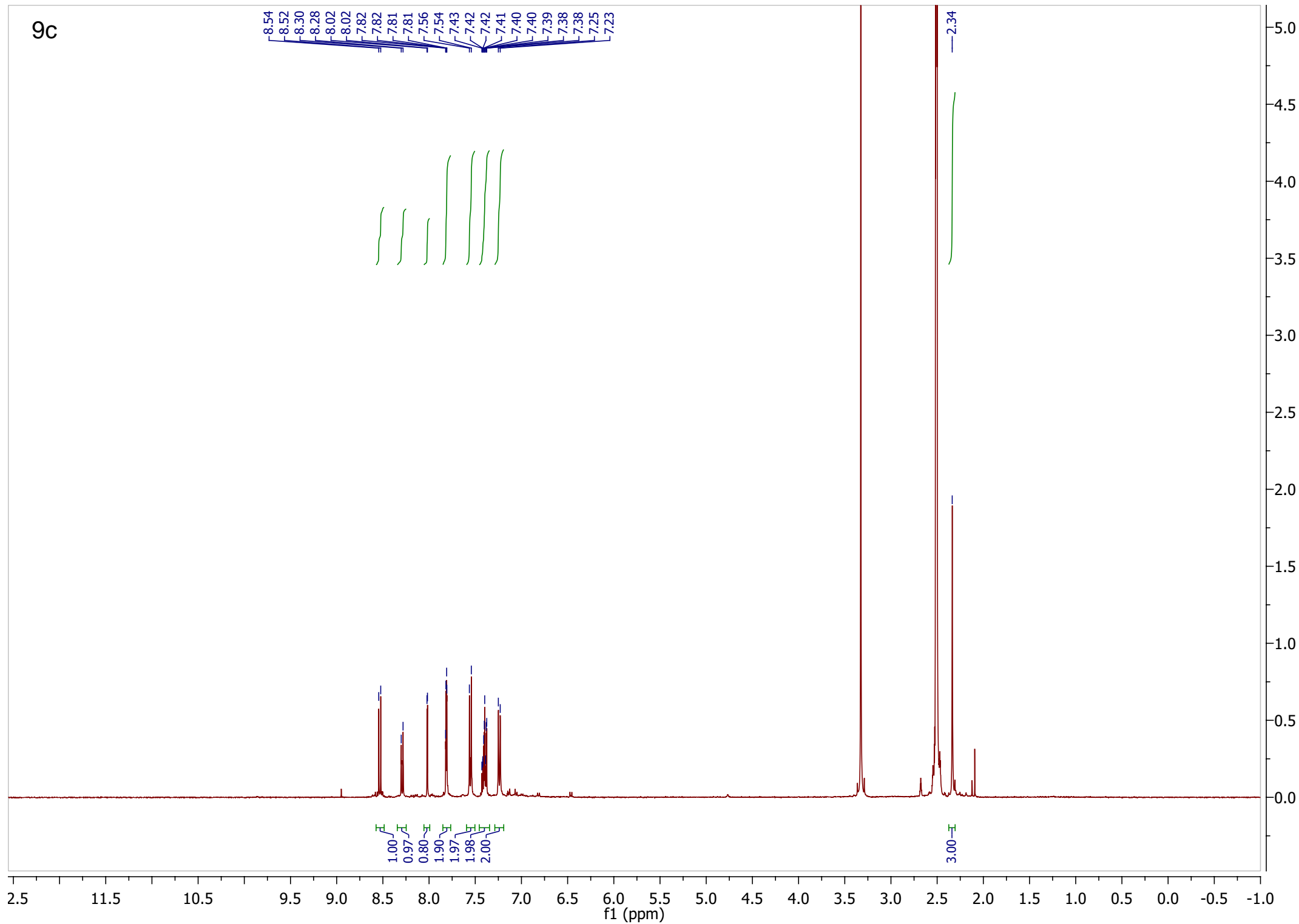

9c

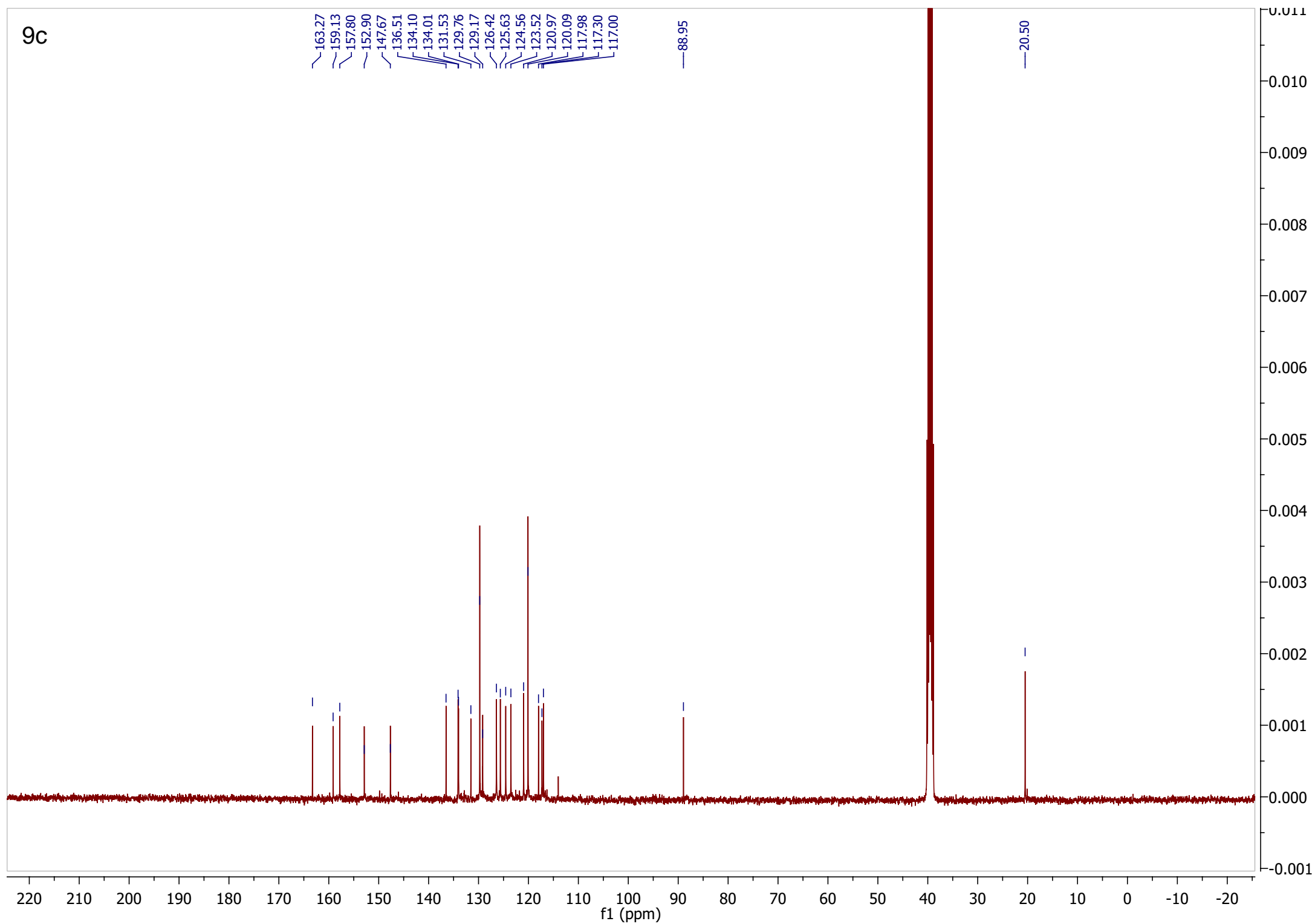

9d

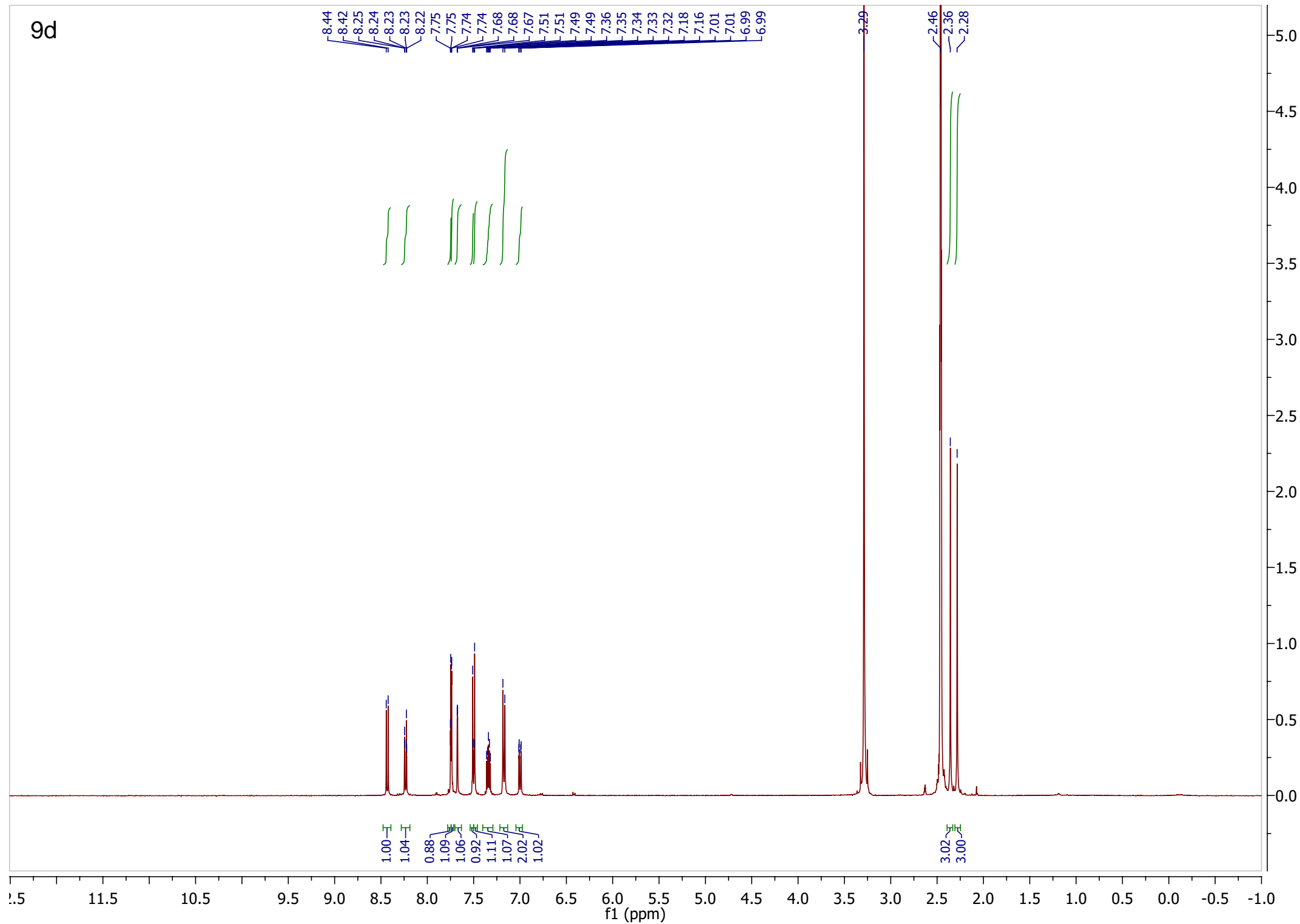

9d

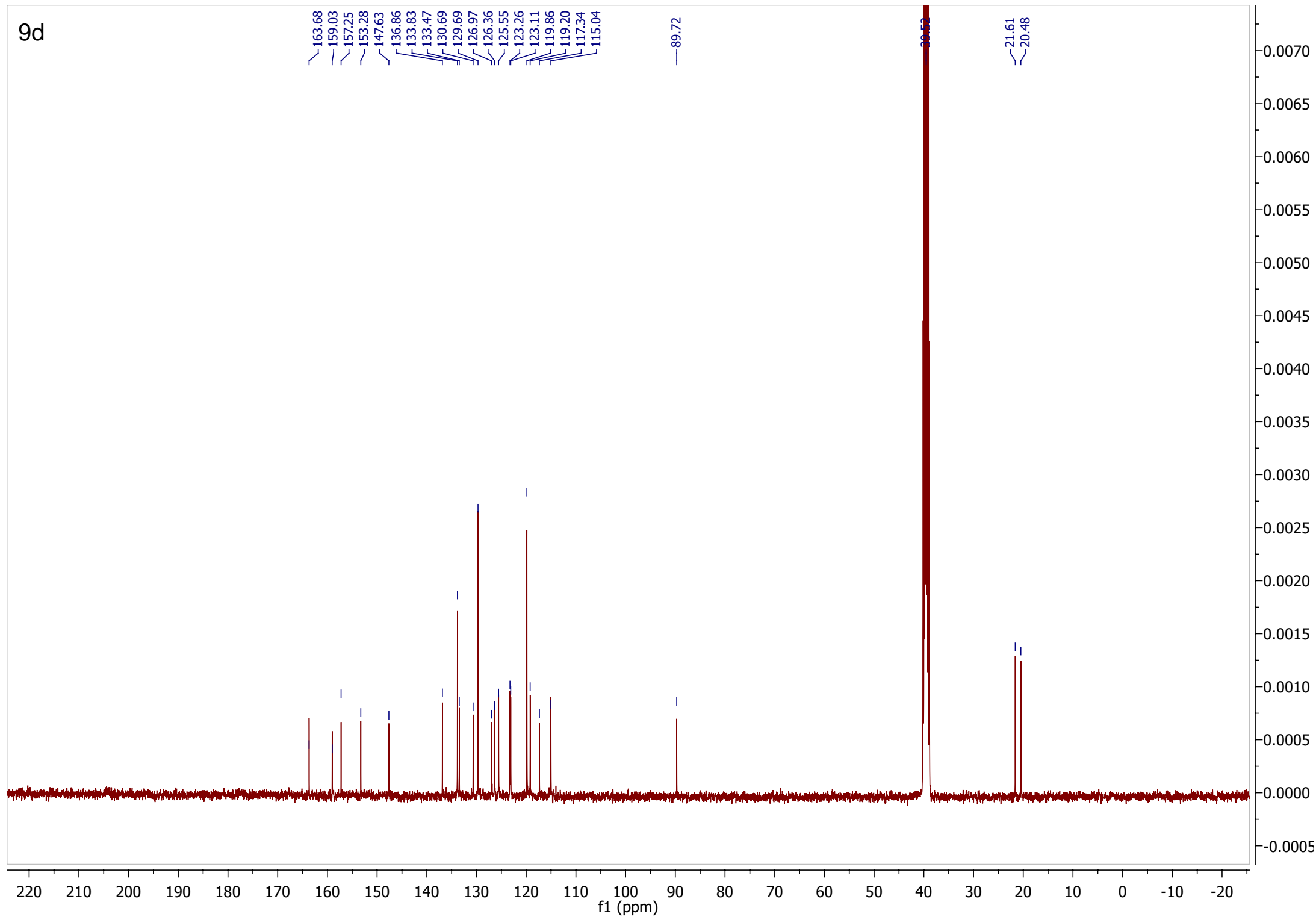

9e

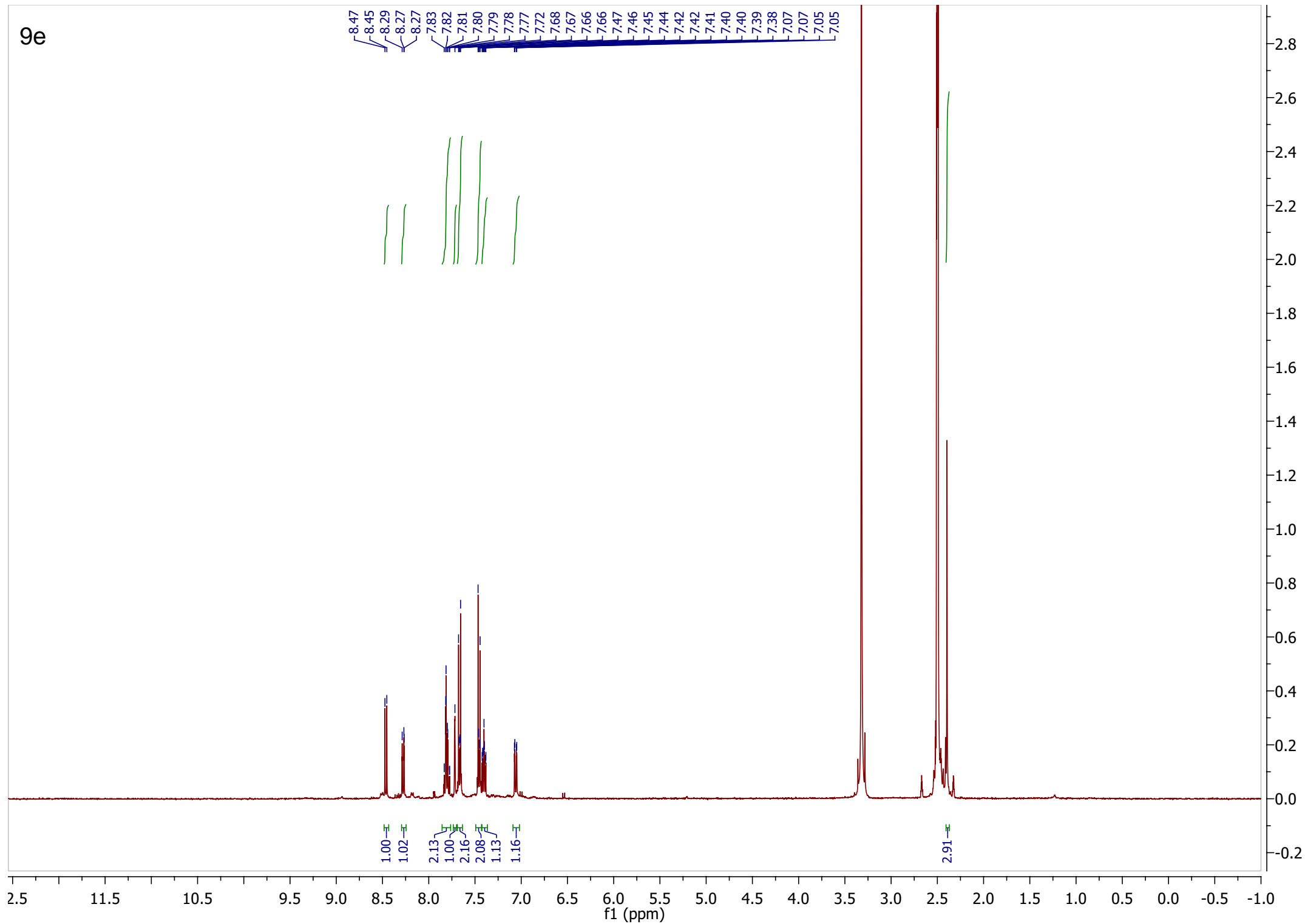

9e

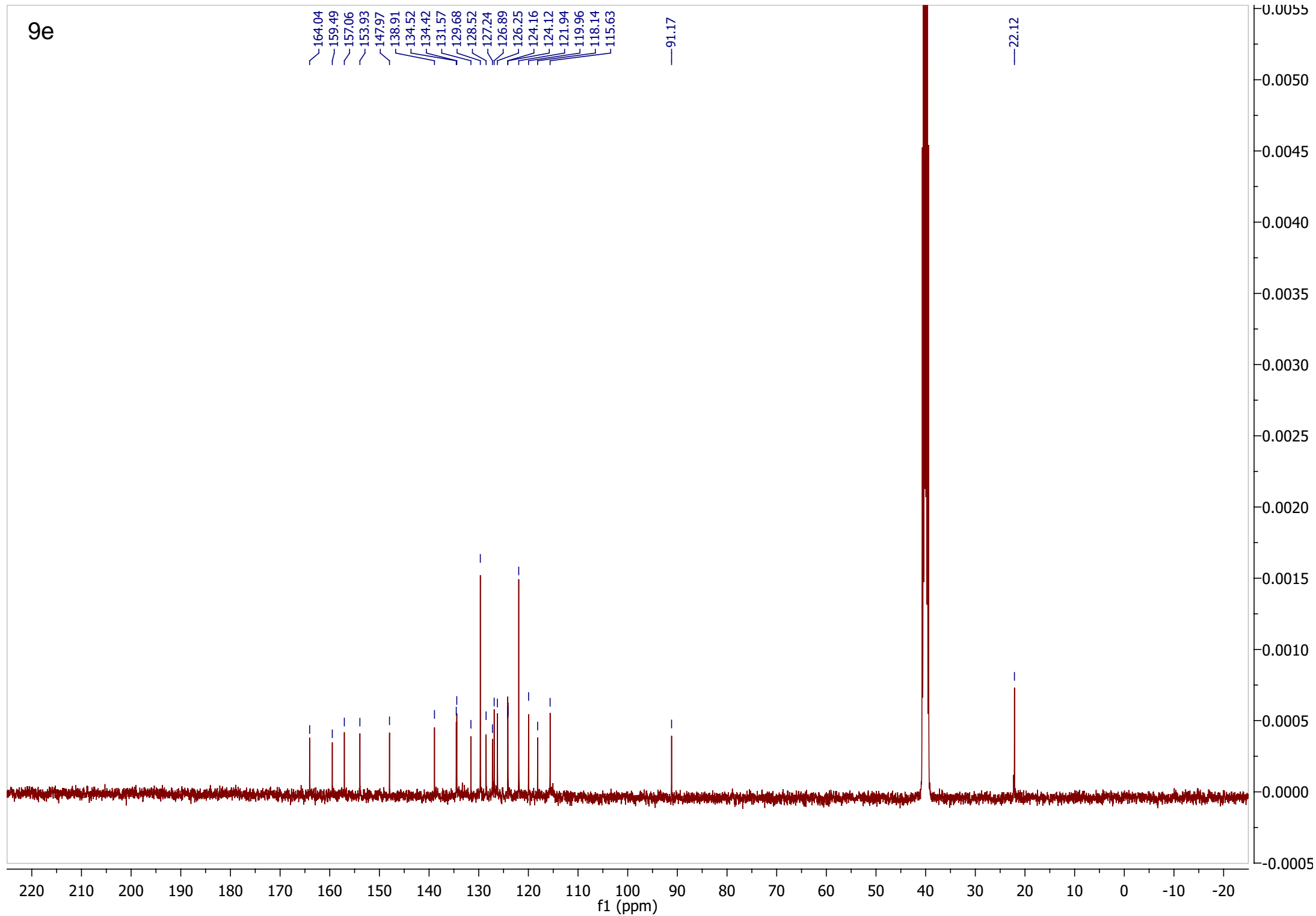

9f

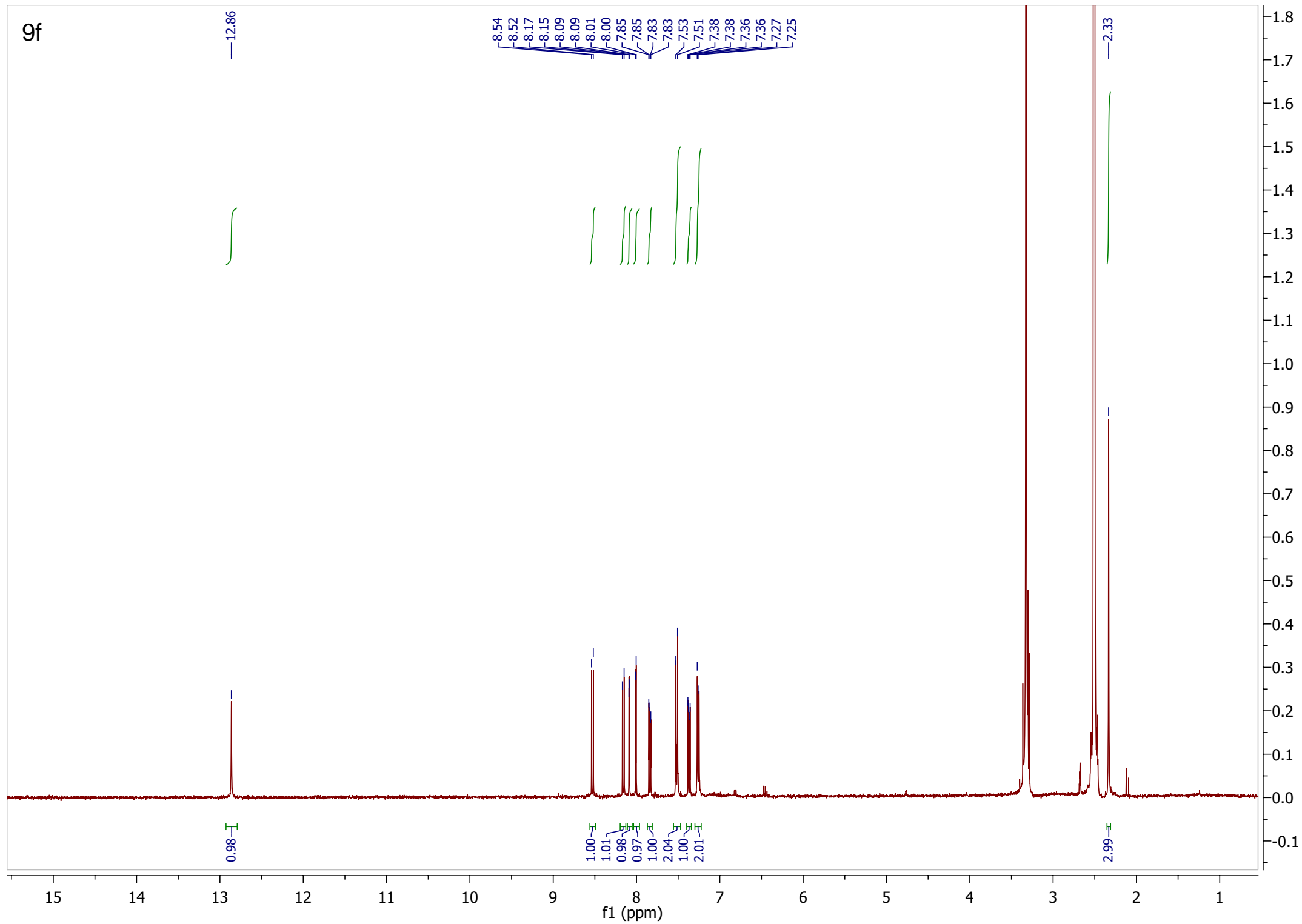

9f

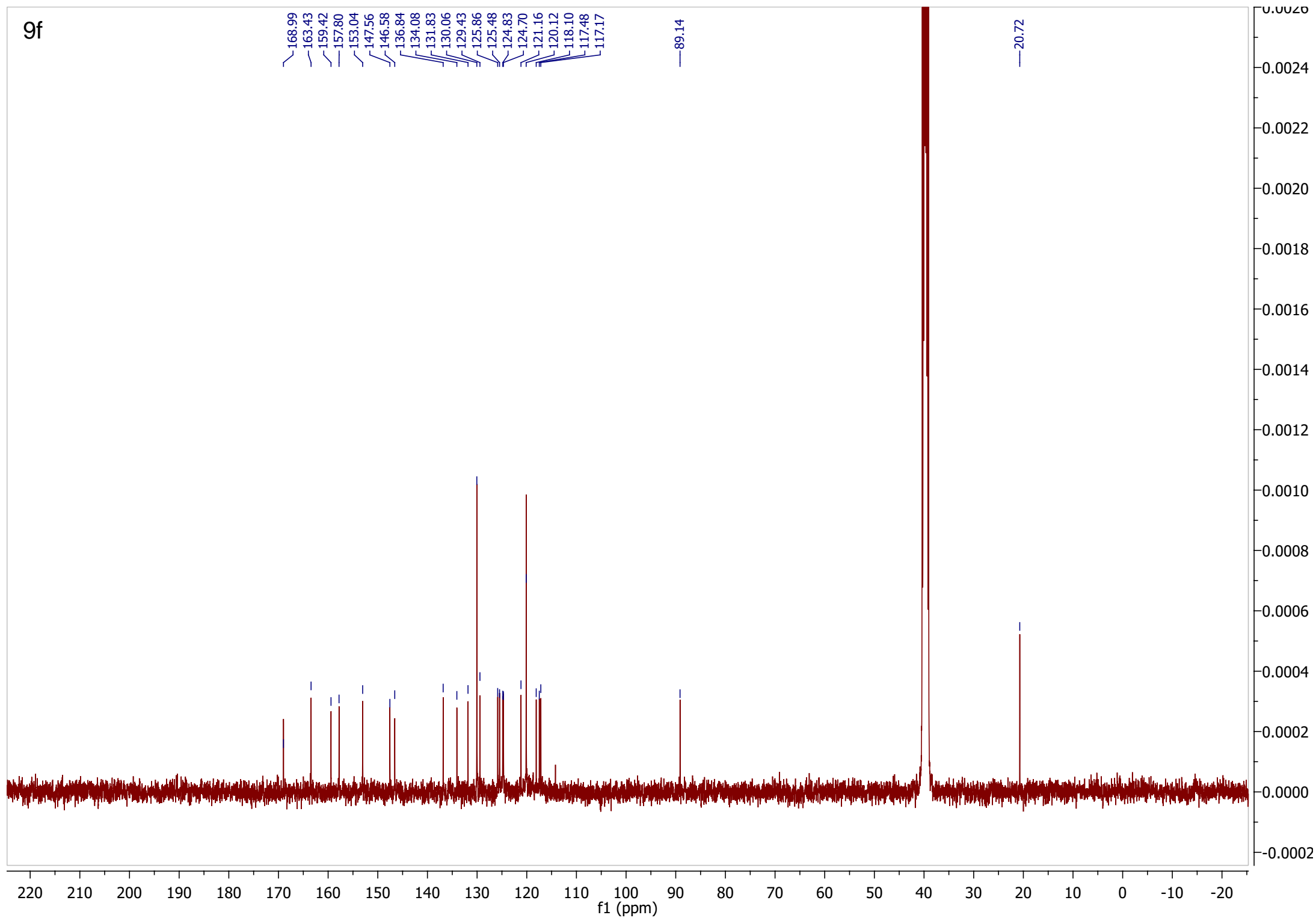

9g

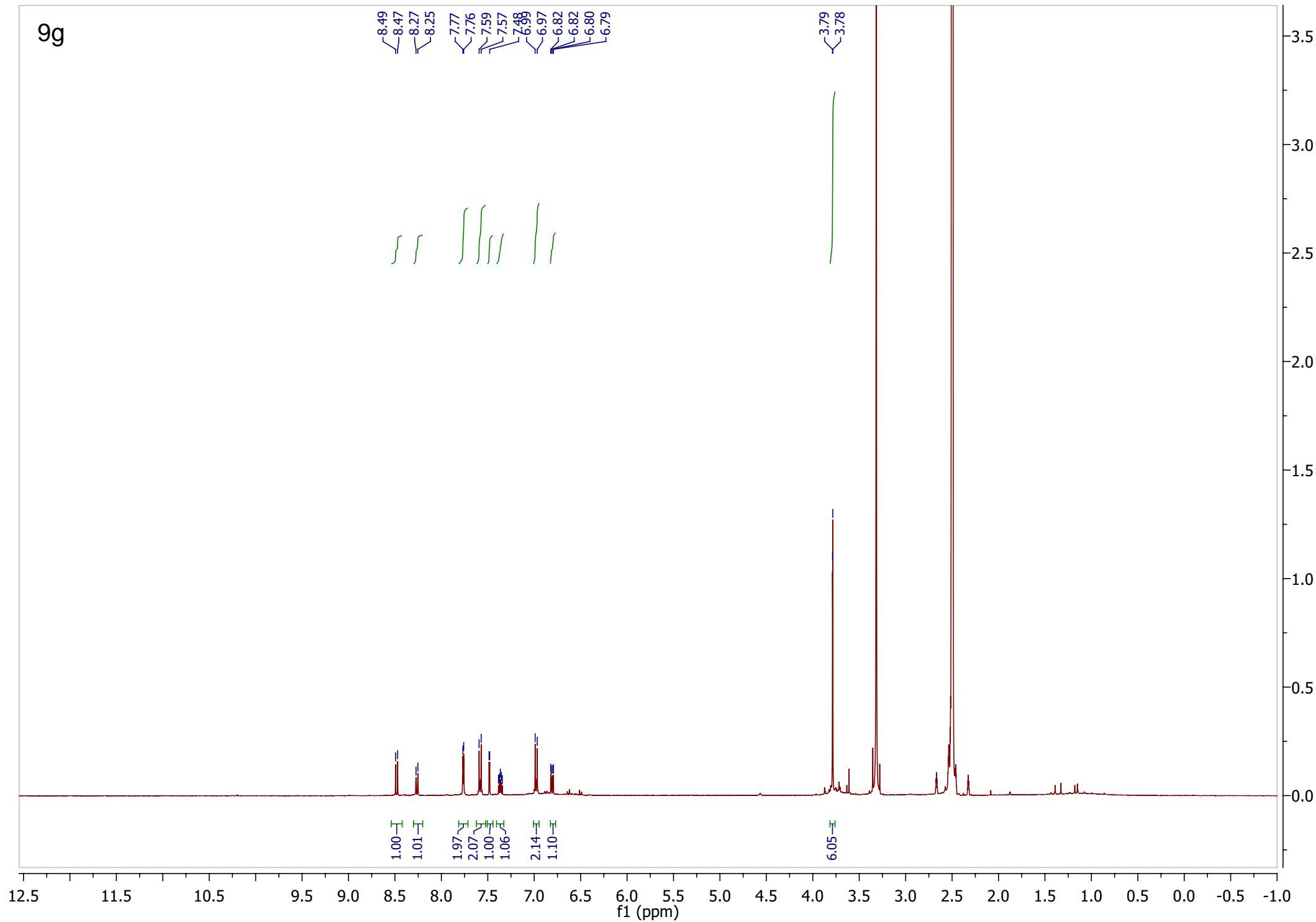

9g

163.80  
159.27  
158.27  
157.58  
156.89  
153.82  
148.08  
134.12  
132.90  
128.91  
127.31  
126.00  
123.55  
122.01  
117.71  
116.33  
114.95  
108.05  
105.06

55.83

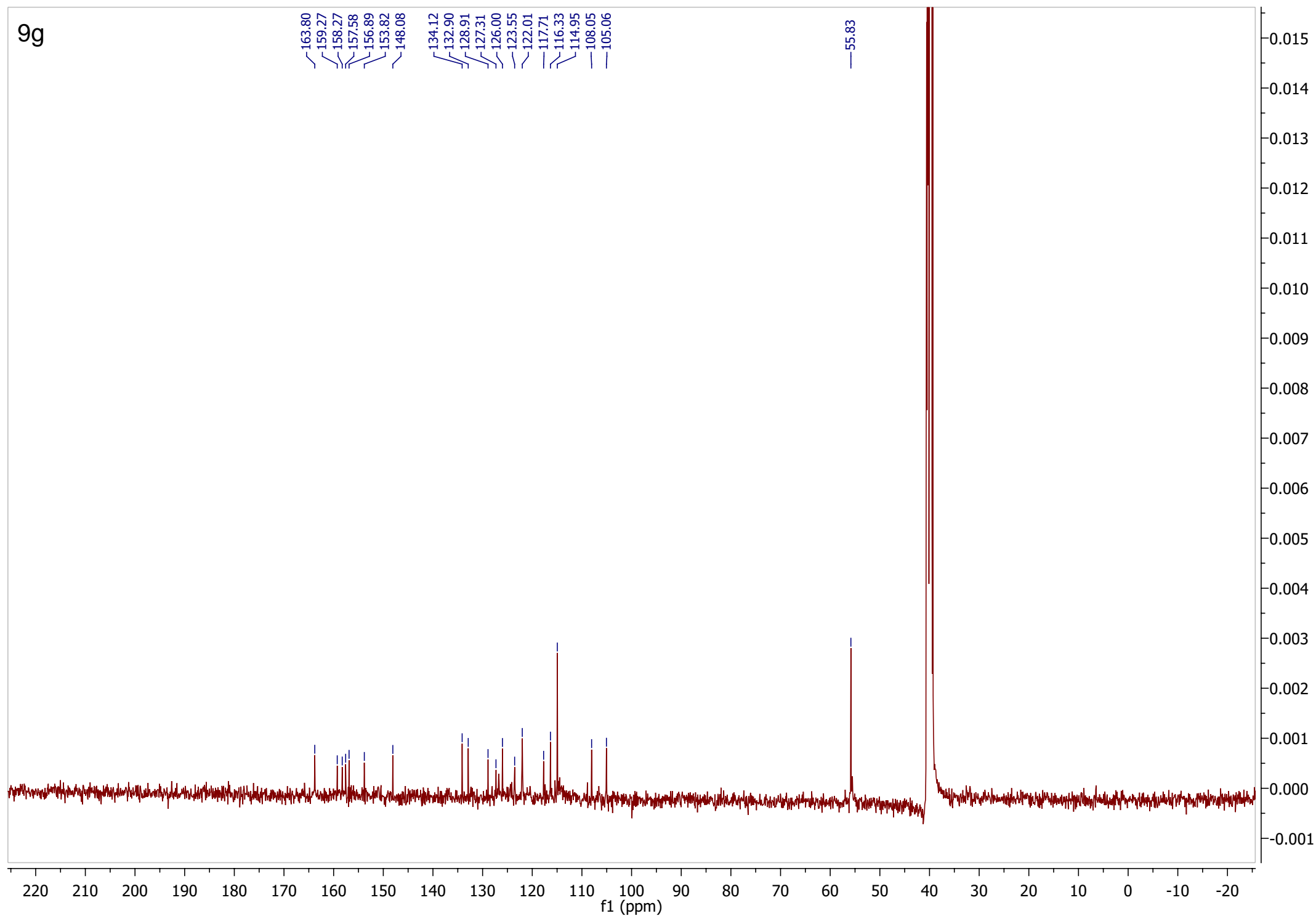

2a

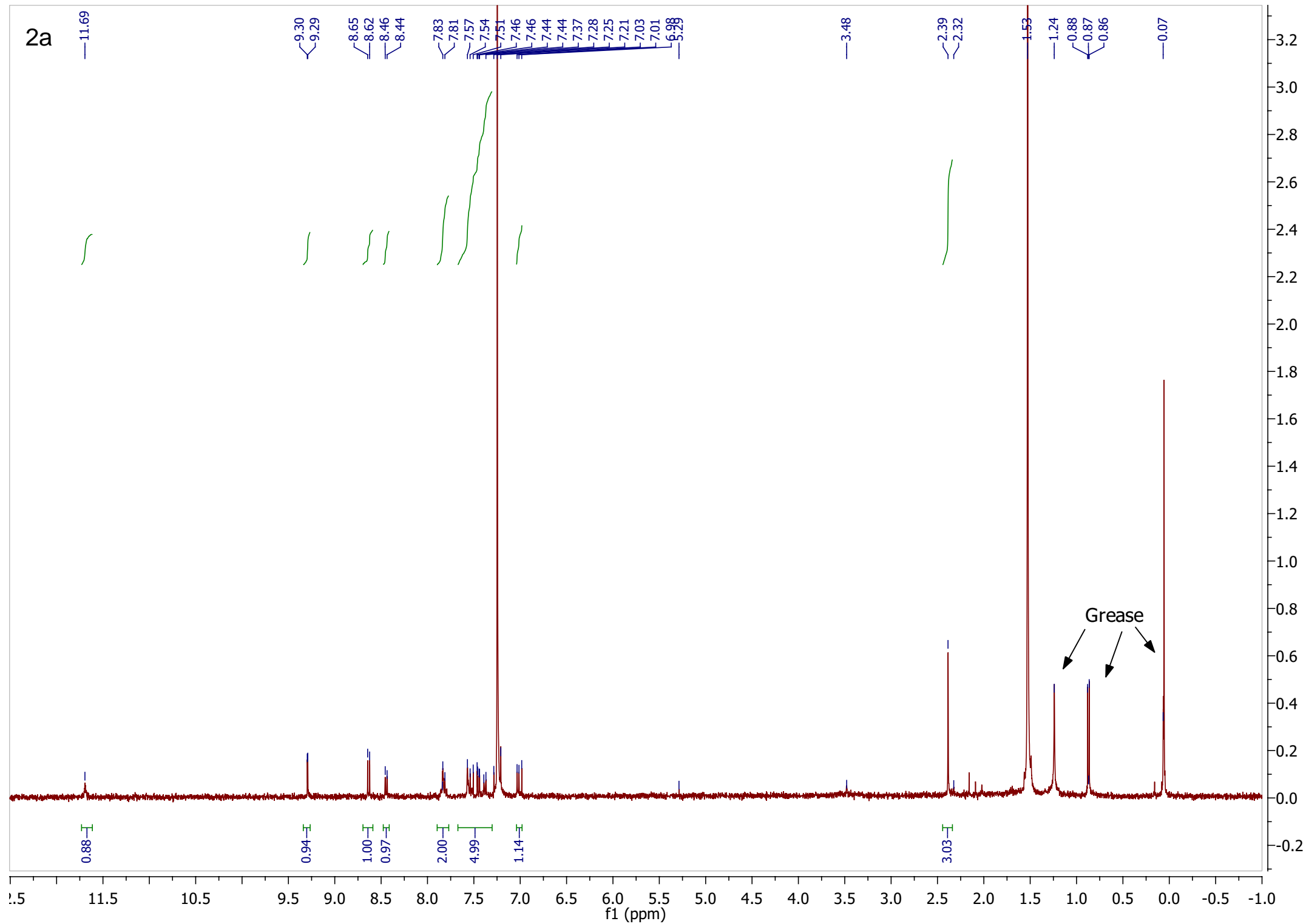

2b

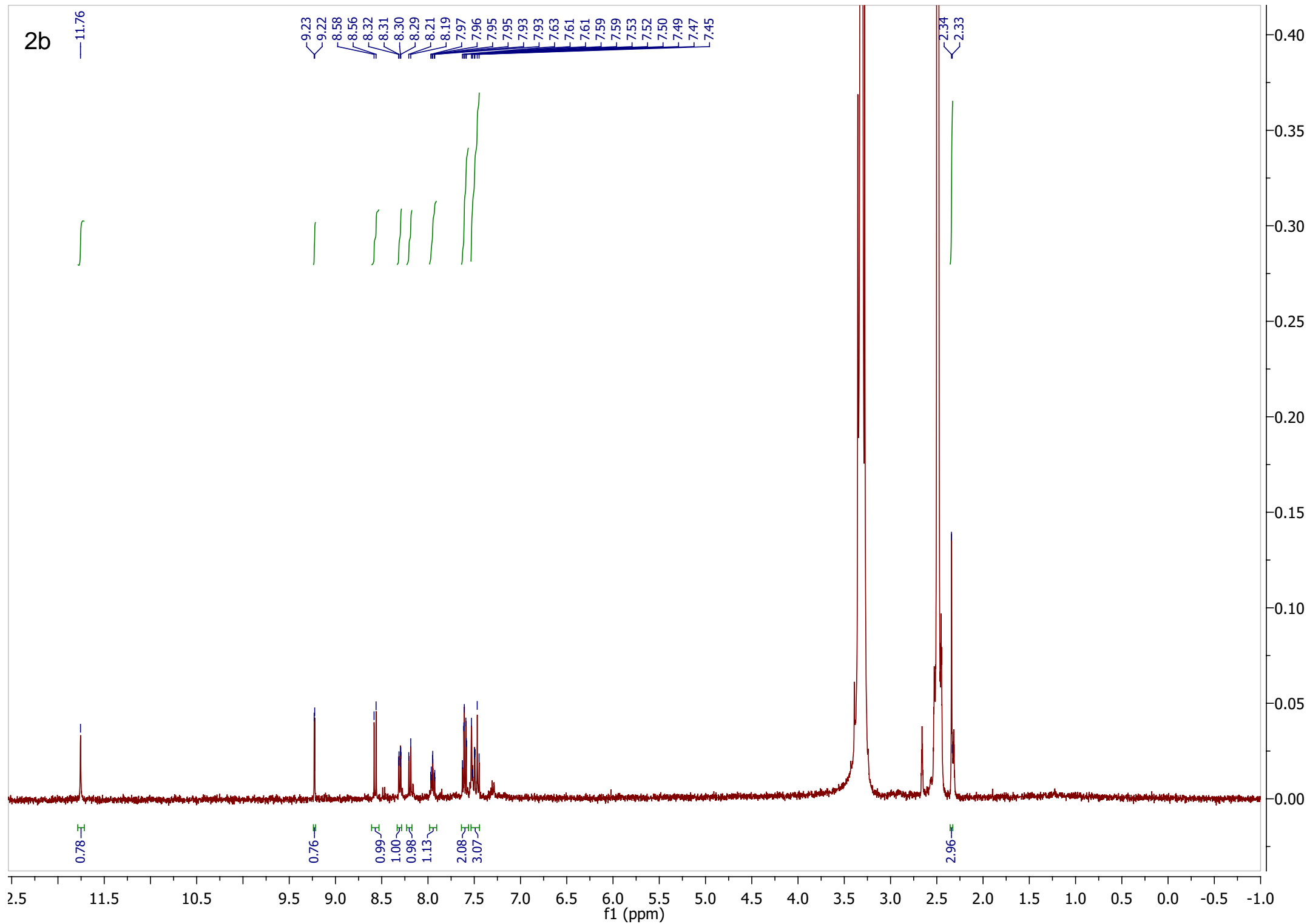

2c

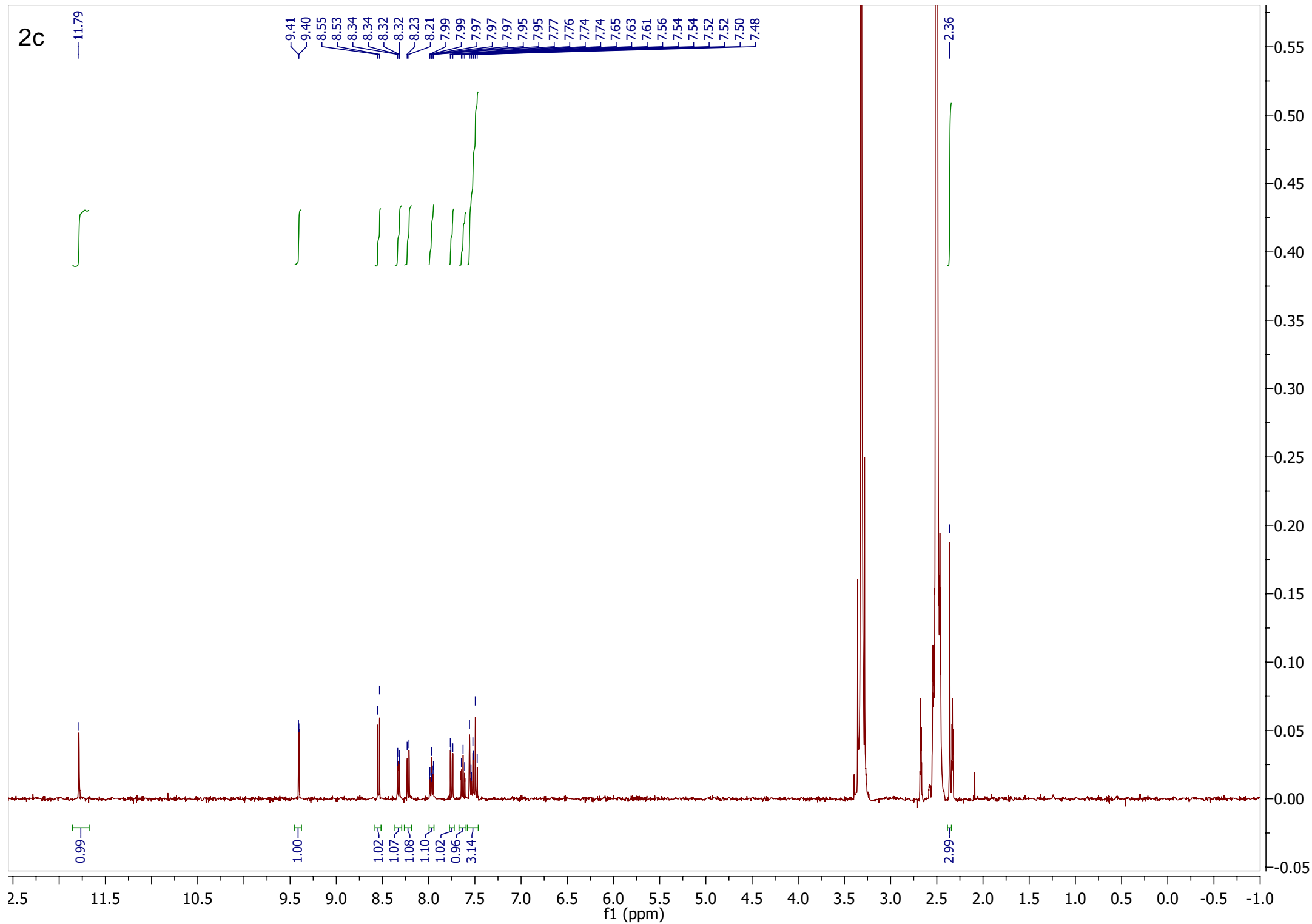

2d

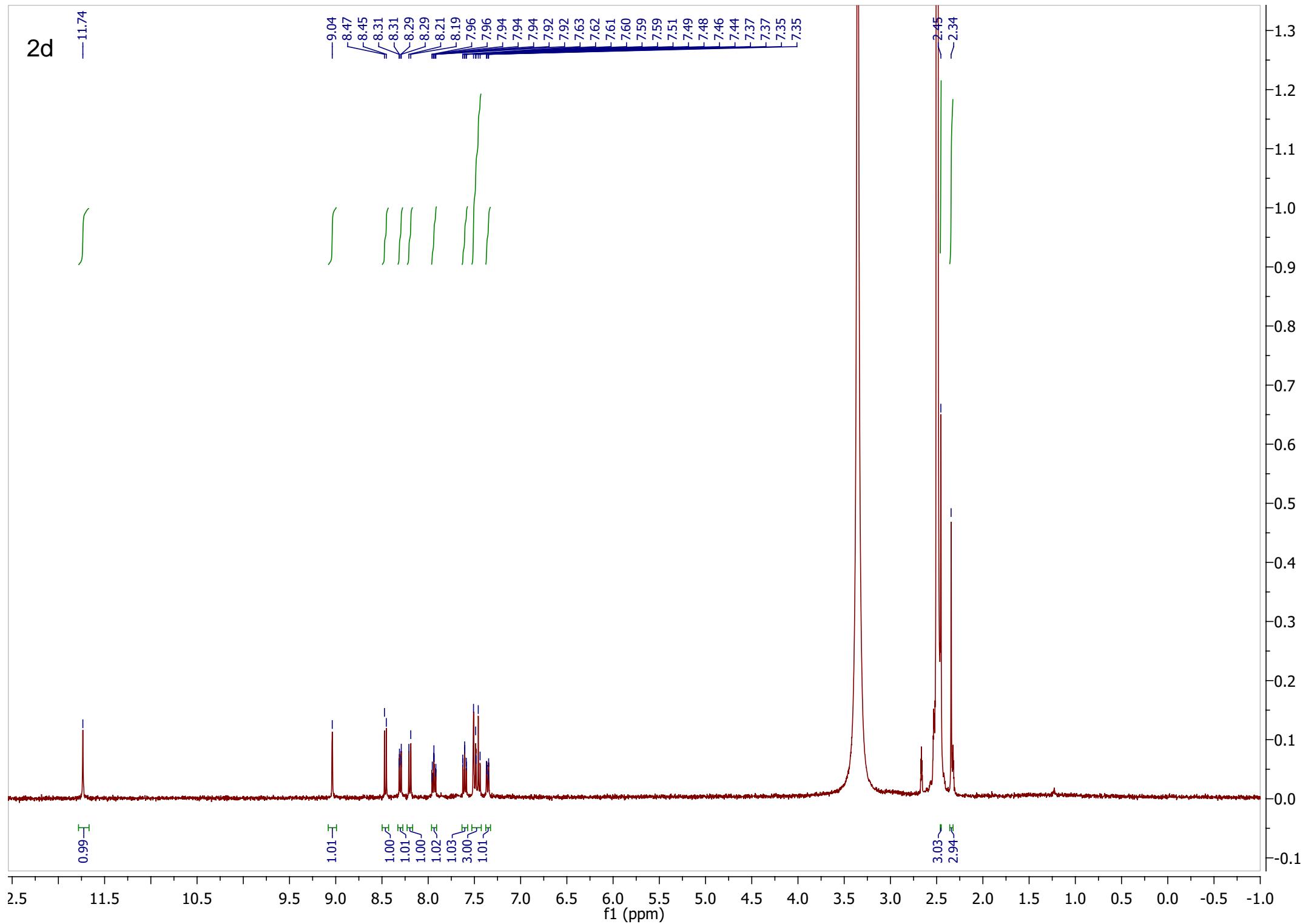

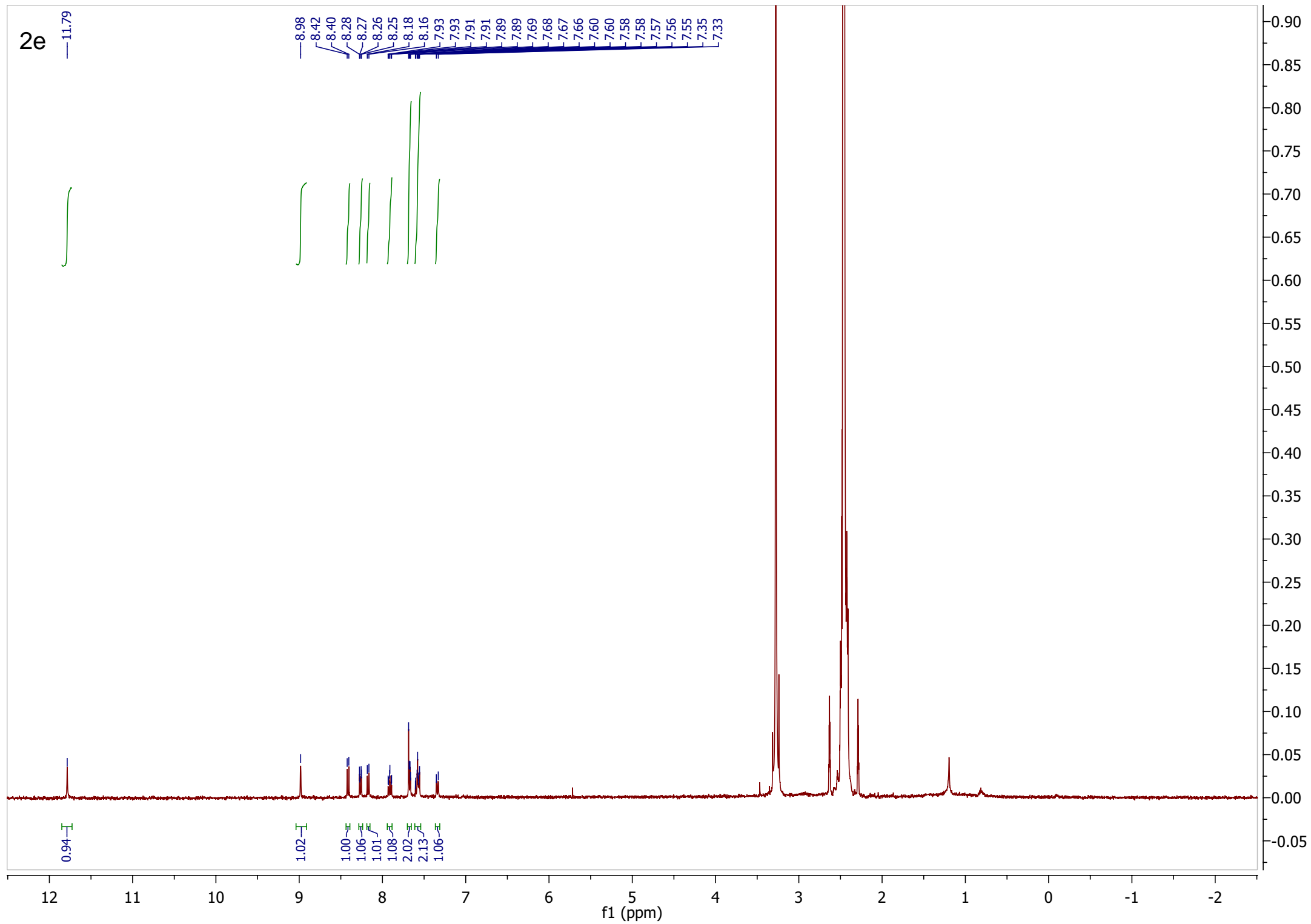

2e

—186.44

—158.64

—153.87

—150.28

—147.12

—137.94

—136.79

—135.67

—134.79

—130.70

—128.42

—127.77

—126.92

—126.40

—124.28

—115.83

—108.45

—21.85

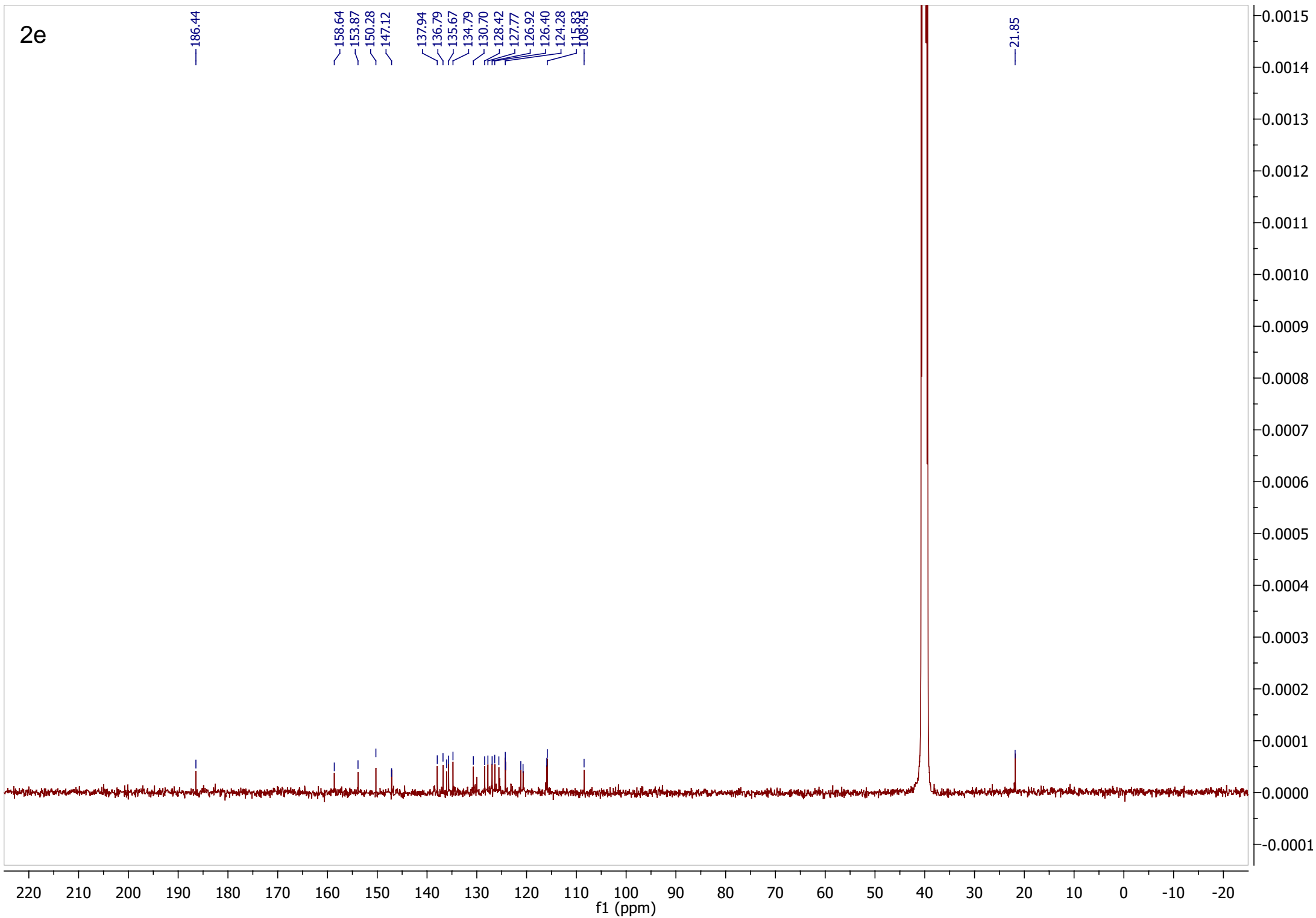

2f

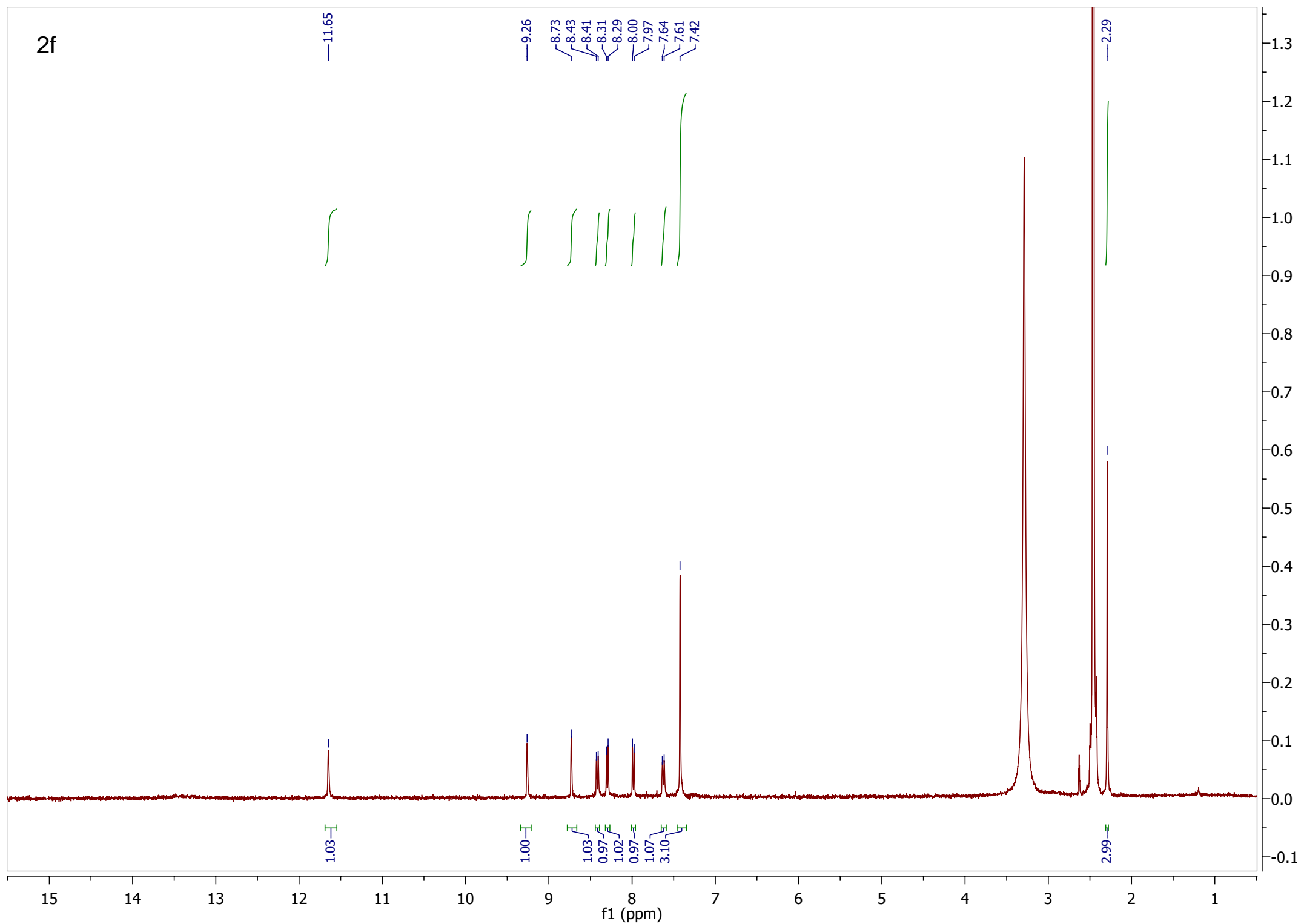

2g

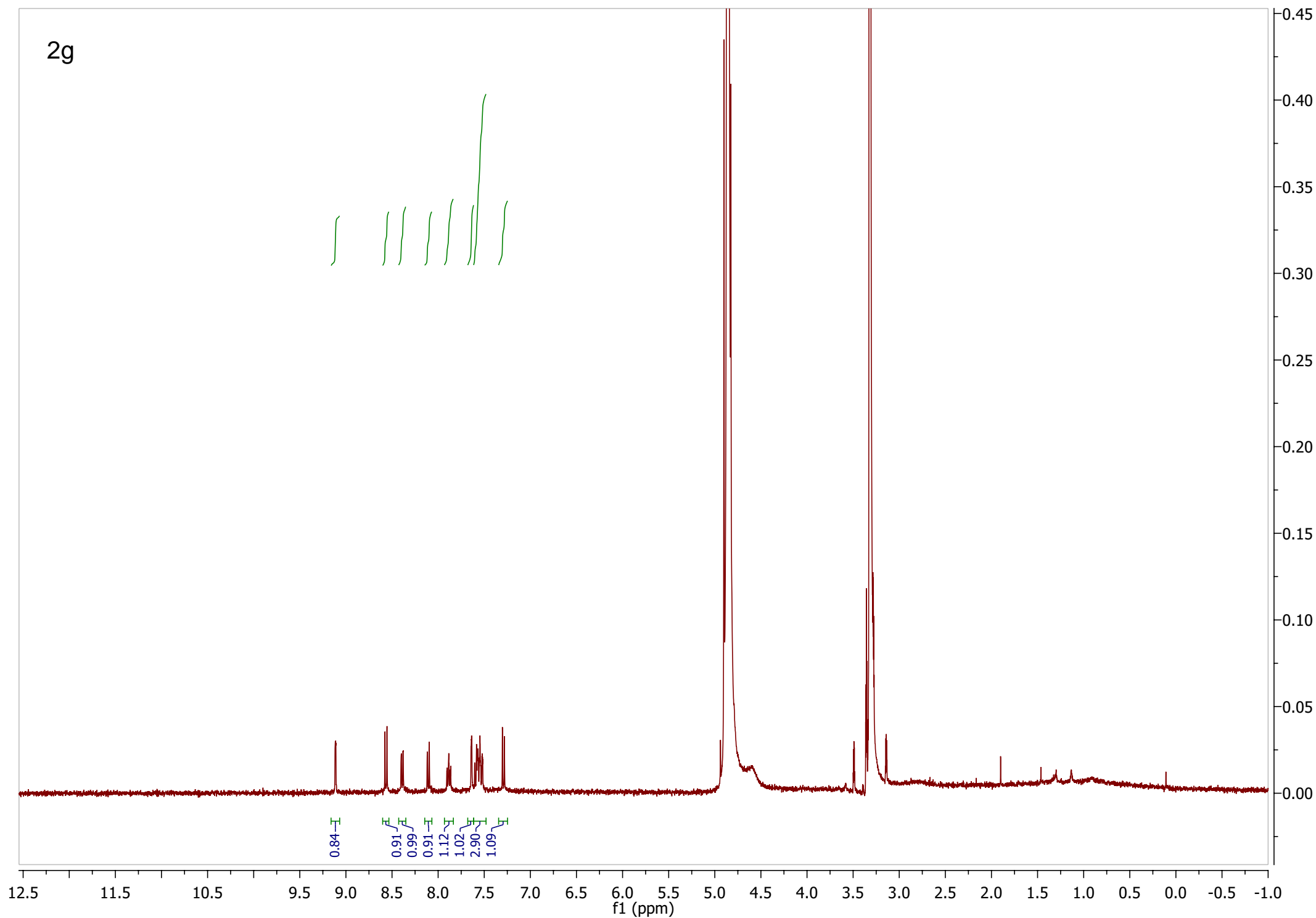

Supplement: Supplementary file 1 [file molecules-27-05038-s001.zip › molecules-1629486-supplementary/Figure S2.pdf]
